# An implementation trial to m**A**nage si**C**kle **CEL**l dis**E**ase through inc**R**eased **A**dop**T**ion of hydroxyur**E**a in Nigeria (**ACCELERATE**): Study protocol

**DOI:** 10.1371/journal.pone.0311900

**Published:** 2025-01-08

**Authors:** Emmanuel Peprah, Joyce Gyamfi, John Patena, Hazal Kayalioglu, Tania Hameed, Gbenga Ogedegbe, Hyungrok Do, Dike Ojji, Deborah Adenikinju, Tayo Ajaye Oba, Maxwell Nwegbu, Hezekiah Isa, Grace Shedul, Alayo Y. Sopekan, Obiageli E. Nnodu

**Affiliations:** 1 Department of Global and Environmental Health, Implementing Sustainable Evidenced-based interventions through Engagement (ISEE Lab), NYU School of Global Public Health, New York, NY, United States of America; 2 Institute for Excellence in Health Equity, NYU Langone Medical Center, New York, NY, United States of America; 3 Department of Biostatistics, NYU Langone Medical Center, New York, NY, United States of America; 4 Cardiovascular Research Unit, Department of Internal Medicine, Faculty of Clinical Sciences, University of Abuja, Gwagwalada, Abuja, Nigeria; 5 Centre of Excellence for Sickle Cell Disease Research and Training (CESRTA), University of Abuja, Abuja, Nigeria; 6 Department of Chemical Pathology, Faculty of Basic Clinical Sciences, College of Health Sciences, University of Abuja, Abuja, Nigeria; 7 Pharmacy Department, University of Abuja Teaching Hospital Gwagwalada FCT, Abuja, Nigeria; 8 Department of Public Health, Non-Communicable Diseases Control Division, Federal Ministry of Health and Social Welfare, Abuja, Nigeria; Public Library of Science, UNITED KINGDOM OF GREAT BRITAIN AND NORTHERN IRELAND

## Abstract

**Background:**

Despite the proven efficacy of evidence-based healthcare interventions in reducing adverse outcomes and mortality associated with Sickle Cell Disease (SCD), a vast majority of affected individuals in Africa remain deprived of such care. Hydroxyurea (HU) utilization among SCD patients in Sub-Saharan Africa (SSA) stands at less than 1%, while in Nigeria, approximately 13% of patients benefit from HU therapy. To enhance HU utilization, targeted implementation strategies addressing provider-level barriers are imperative. Existing evidence underscores the significance of addressing barriers such as inadequate healthcare worker training to improve HU adoption. The ACCELERATE study aims to evaluate the adoption of HU among providers through the Screen, Initiate, and Maintain (SIM) intervention, facilitated by healthcare worker training, clinical reminders, and task-sharing strategies, thereby enhancing patient-level SCD management in Nigeria.

**Methods:**

This study will implement the SIM intervention, encompassing patient screening, initiation of HU treatment, and maintenance of dosage, which will be implemented via the TAsk-Strengthening Strategy for Hemoglobinopathies (TASSH TCP), derived from our team’s TAsk-Strengthening Strategy for Hypertension control (TASSH) trials. Employing a sequential exploratory mixed-methods approach within the Exploration, Preparation, Implementation, and Sustainment (EPIS) framework, this study will assess SIM adoption by providers in Nigeria. The primary outcome is the rate of SIM adoption at clinical sites at 12 months, with secondary outcomes including sustainability/maintenance of SIM intervention and implementation fidelity.

**Discussion:**

This study’s findings will offer crucial insights into effective SCD management strategies, leveraging existing SCD clinical networks and resources in Nigeria to enhance HU adoption among providers in a scalable and sustainable manner. Additionally, the study will inform best practices for implementing HU therapy in resource-constrained settings, benefiting healthcare providers, policymakers, and stakeholders invested in improving SCD care delivery.

**Trial registration:**

NCT06318143.

## Background

Sub-Saharan Africa (SSA) shoulders the heaviest burden of sickle cell disease (SCD) globally, affecting over 300,000 individuals [1, 2]. This hereditary blood disorder results in anemia, severe pain, and various vaso-occlusive complications, including acute chest syndrome (ACS), disproportionate hospitalizations, and premature mortality, thereby imposing substantial financial, social, and psychosocial strains on affected individuals, families, and healthcare systems alike [3–5]. Alarmingly, it is estimated that over 300,000 babies are born with SCD annually in SSA, underlining the urgent need for effective intervention [6]. SCD represents a progressively debilitating, chronic multi-organ affliction, with a staggering 30–50% incidence of disability and unemployment, and tragically stands as the primary cause of stroke in children and adolescents [6]. While comprehensive clinical care initiatives in the United States (US) have markedly reduced childhood mortality attributed to SCD by 70% [7], such evidence-based practices (EBPs) have yet to be widely disseminated across Africa, where a heartbreaking 50–90% of children with SCD succumb before reaching the age of 10 [8, 9].

Nigeria, within SSA, bears the highest prevalence of SCD, with an estimated 4 million cases among its total population of 202 million, the majority of which remain undiagnosed and unlinked to care [10]. Annually, approximately 150,000 babies are born with SCD in Nigeria alone [11], a burden that could be significantly alleviated through early diagnosis and supportive measures such as penicillin prophylaxis and hydroxyurea (HU) treatment [12, 13]. Unfortunately, the full-scale implementation of HU as an evidence-based intervention to enhance health outcomes for SCD remains limited for eligible patients in Nigeria, compounded by low adoption rates among caregivers.

Despite HU therapy’s proven efficacy in mitigating SCD-related complications—including painful episodes, ACS, severe anemia, and hospitalizations—it remains underutilized in Nigeria, with only a fraction of eligible patients receiving this potentially life-saving treatment [14–16]. Challenges such as the need for specialized training in HU administration and monitoring, alongside provider-level barriers, hinder its widespread adoption. A study by Galadanci and colleagues revealed that only a minority of SCD specialist health institutions in Nigeria prescribed HU [17], with even fewer patients maintaining its use, suggesting significant impediments at the provider level [18–21].

Regrettably, the vast majority of individuals with SCD in Africa are deprived of evidence-based healthcare interventions, including newborn screening, health education, infection prophylaxis, optimal nutrition and hydration, blood transfusions, transcranial Doppler screening, and HU therapy—despite their potential to dramatically reduce adverse outcomes and mortality associated with the condition [14–16]. The utilization of HU therapy in SSA stands at less than 1%, primarily due to the significant costs and challenges associated with laboratory monitoring [22].

To address these barriers and enhance HU adoption, a comprehensive understanding of provider-level challenges is imperative [23–26]. Our survey of 87 healthcare providers within the NHLBI-funded Sickle Pan African Research Consortium (SPARCO) network—encompassing Nigeria, Ghana, and Tanzania—revealed key obstacles, including inadequate formal training, absence of clinical reminders, and the scarcity of hematologists [27]. Addressing these challenges through targeted training initiatives for non-hematologist physicians and nurses could substantially improve prescription practices and ensure that eligible SCD patients receive the benefits of HU therapy, thereby advancing the management of this debilitating disease.

To bridge the critical gap between research and practice, we are implementing provider-level education, clinical support, and task-sharing/shifting, all evidence-based strategies for effectively managing SCD. A key challenge in Nigeria and across SSA is the severe shortage of hematologists. As of 2021, there were only 3.9 physicians and 15.6 nursing/midwifery personnel per 10,000 patients in Nigeria (with only about 389 hematologists for a country of 202 million people), a shortfall exacerbated by the migration of healthcare workers to high-income countries for better living standards, salaries, and political stability [28]. This shortage underscores the urgent need to enhance the capacity of non-hematologists in SCD management, making task-sharing a promising solution.

Task-sharing in this case entails the rational distribution of responsibilities among healthcare teams, encompassing hematologists, non-hematologist physicians, and nurses. This approach proves invaluable in resource-limited settings grappling with healthcare workforce crises, as evidenced by our previous successful utilization of task-sharing for chronic diseases like HIV and hypertension in West Africa. Additionally, findings from the NHLBI-funded REACH trial conducted across four African countries—Angola, Congo, Kenya, and Uganda—demonstrate significant reductions in transfusion utilization for children with SCD following treatment with HU [29].

However, despite these promising findings, a large-scale implementation trial assessing HU adoption within service providers in Nigeria has yet to be conducted. To address this gap, the mAnage siCkle CELl disEase through incReased AdopTion of hydroxyurEa in Nigeria (ACCELERATE) study leverages evidence-informed strategies derived from the REACH trials. We have adapted the REACH protocol into a simplified algorithm—Screening, Initiation, and Maintenance (SIM)—to facilitate provider training and streamline SCD management in Nigeria. Our implementation strategy for improving SCD management in Nigeria uses a practical and replicable evidence-based task-sharing strategy, TAsk-Strengthening Strategy for Hemoglobinopathies (TASSH), adopted from our TAsk-Strengthening Strategy for Hypertension control (TASSH) trials in Ghana and Nigeria containing the following essential implementation strategies: i) Training healthcare workers/providers to be more patient-centered in clinical consultations, ii) Clinical reminders, and iii) Practice facilitation (TCP) known as (TASSH TCP) for SCD management. This algorithm, combined with healthcare worker training, clinical reminders, task-sharing, and task-shifting, constitutes the TASSH TCP approach aimed at fostering the adoption and sustainability of HU intervention effects beyond the study period.

The ACCELERATE study is guided by the Exploration, Preparation, Implementation, and Sustainment (EPIS) model, which provides a structured framework for evaluating change processes and long-term impacts [30]. Central to this framework is the Exploration and Preparation phase, where we engage stakeholders to understand local clinical practices, decision-making processes, and perspectives on HU implementation. This phase also entails gathering insights to tailor healthcare worker training to suit the Nigerian healthcare context, ensuring cultural sensitivity and relevance. Insights gleaned from the exploration and preparation phase will inform subsequent implementation and sustainment phases of EPIS, facilitating the translation of research findings into tangible improvements in SCD care delivery and outcomes. Through collaboration with healthcare professionals, patients, caregivers, and community stakeholders, we aim to develop intervention strategies that are evidence-based, culturally appropriate, and responsive to the unique challenges of SCD management in Nigeria.

### Study aims and hypothesis

This comprehensive mixed-methods study will implement a multifaceted educational and clinical support intervention aimed at enhancing provider capacity and characterizing the readiness of 20 clinical sites to adopt the SIM approach for improved SCD management. Subsequently, we will conduct a cluster Randomized Controlled Trial (RCT) to assess the impact of our implementation strategy, termed TASSH TCP, which includes educational interventions (healthcare worker training), clinical reminders, and practice facilitation, against the receipt of educational information only (control arm) on TASSH TCP across the 20 clinical sites over 12 months.

Our primary outcome is the adoption of the SIM intervention, measured by an increase in HU prescription rates for eligible SCD patients. We hypothesize that clinical sites randomized to the experimental arm of the cluster RCT will exhibit higher levels of SIM adoption compared to those in the control arm. Additionally, our secondary aims include evaluating the sustainability/maintenance of the intervention across clinical sites at 24 months. We anticipate that factors related to the inner organizational context, outer context, and implementation process will influence both the adoption and sustainability of the SIM intervention at clinical sites.

## Methods

### Ethical approval and trial registration

Ethics approval to conduct the study was obtained from the National Health Research and Ethics Committee (approval number: NHREC/01/01/2007-19/12/2023) and the University of Abuja (approval number: UATH/HREC/PR/2024/02/145) in Nigeria, and New York University (study number: i24-00437). The study was registered with ClinicalTrials.gov (registration number: NCT06318143).

### Study design

**Fig 1:**
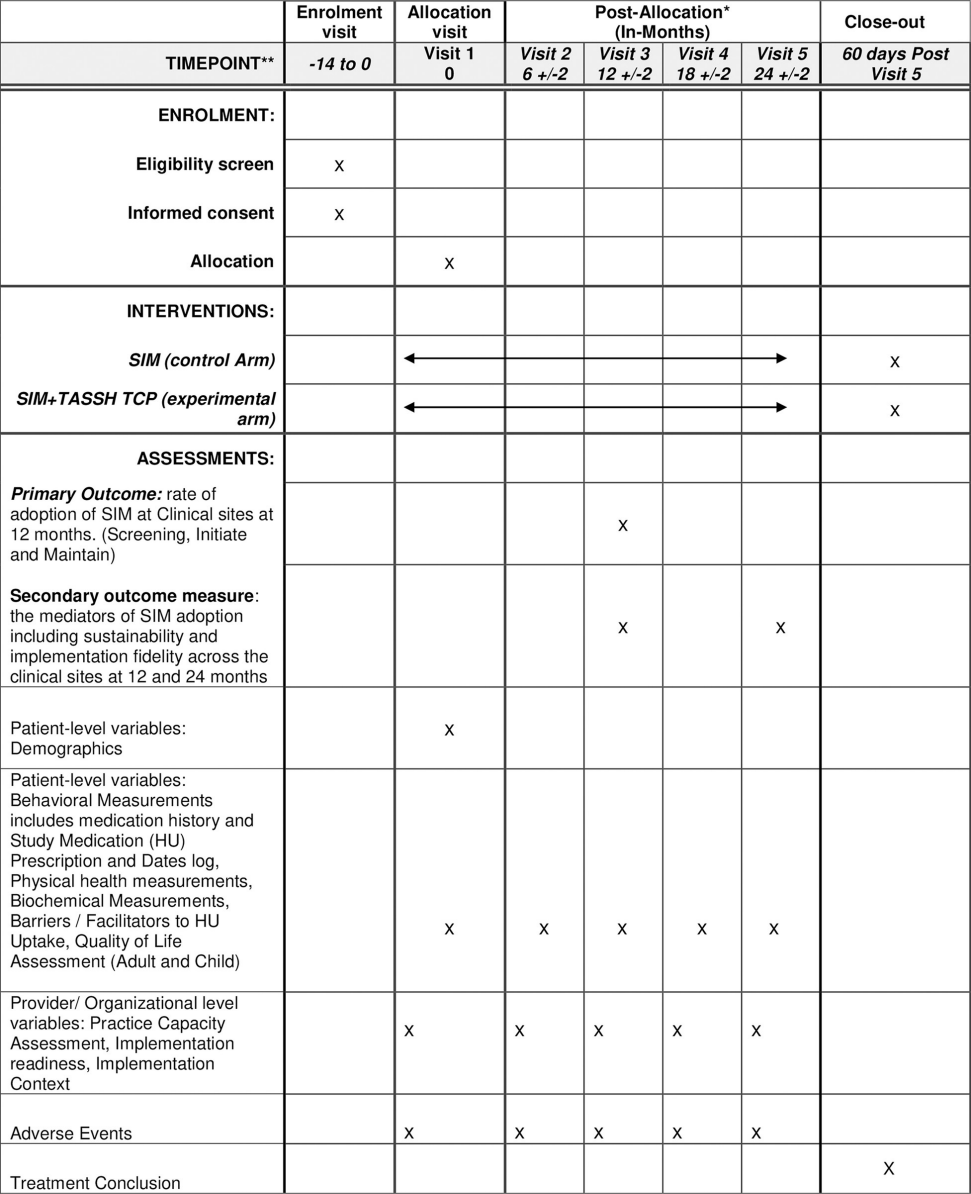
ACCELERATE SPIRIT schedule. *Note: Visit timepoints can range from monthly to every three months depending on patient needs. The above visit timepoints reflect the period of outcome data collection.

**Fig 2:**
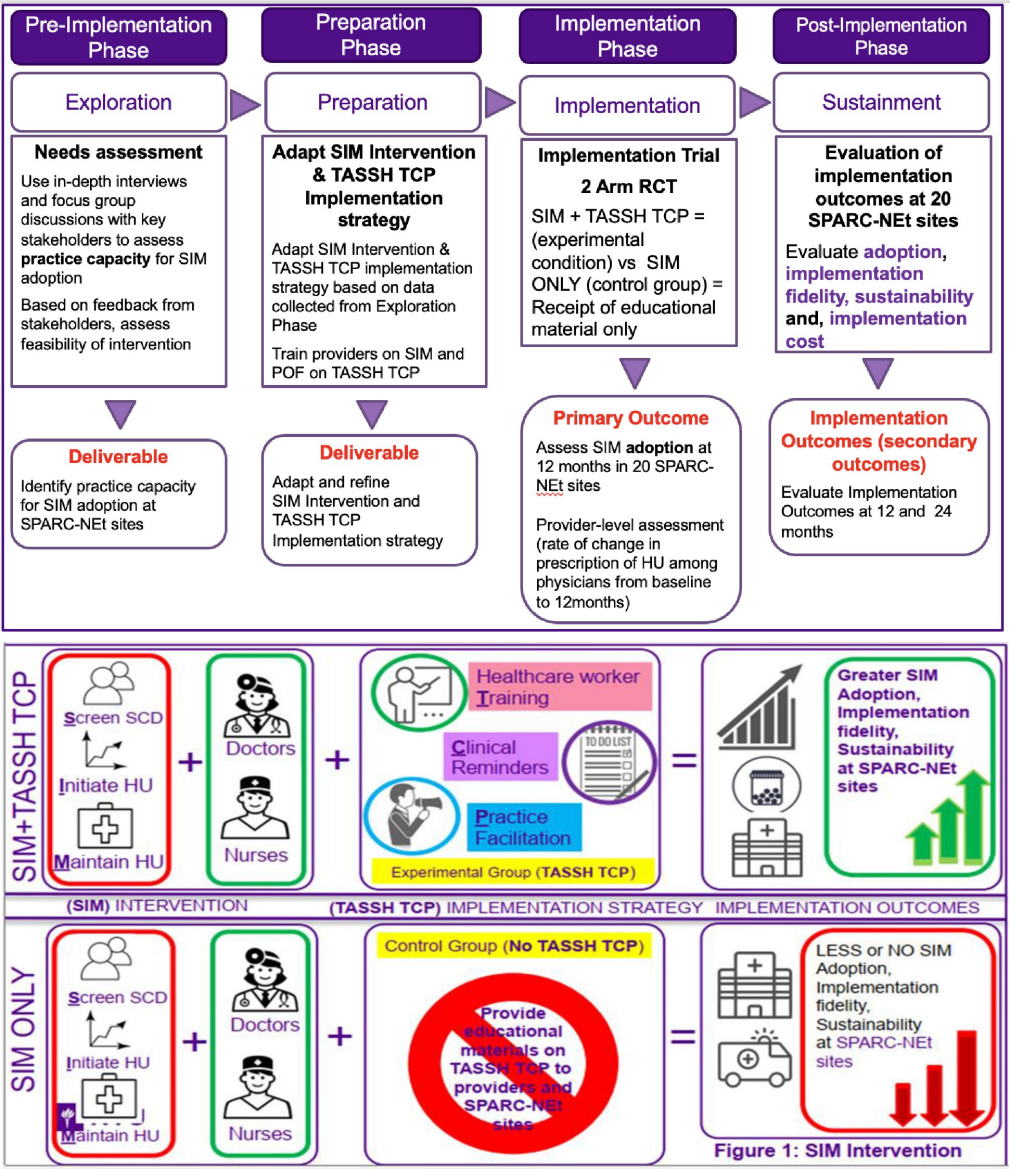
**A.** TASSH TCP Study designed aligned with EPIS framework. **B.** TASSH TCP intervention components and overall structure of implementation trial.

The overall schedule depicting study components and patient time commitments is outlined in accordance with the SPIRIT guidelines in Fig 1. Utilizing a robust mixed-methods study design, our investigation unfolds across four distinct phases of the EPIS framework (see Fig 2A), meticulously examining the multifaceted landscape of HU utilization at the provider level. Simultaneously, we will assess the capacity of Nigerian clinical sites to embrace the SIM approach. Our inquiry extends to scrutinizing the inner organizational context, meticulously examining factors such as the existing SCD management protocols, staffing profiles (comprising nurses, hematologists, and non-hematologist physicians), and prevailing prescription practices among providers.

In the initial Exploration Phase of EPIS, our objective is to delve into the facilitators and barriers influencing the prescription of HU. Through qualitative stakeholder interviews conducted at the provider level, we aim to unravel the nuanced attitudes and perceptions that shape providers’ decisions regarding HU prescriptions for SCD patients. The outcomes of these interviews will illuminate potential barriers hindering HU prescription. For instance, if concerns regarding medication costs emerge prominently during these qualitative sessions, we will actively address them by disseminating information that underscores the coverage of HU under the National Health Insurance Authority Act of 2022 [31]. We will explore the inner organizational context to adapt a tailored intervention, including examining the SCD management standard protocol at clinical sites, staffing data (i.e., numbers of nurses, hematologists, and non-hematologist physicians available), and provider prescription practices. This phase of exploration promises to enrich our understanding of the specific contextual factors surrounding the initiation of provider training for HU in Nigeria. Providers and patients from diverse clinical sites will be recruited to participate, ensuring a comprehensive representation of perspectives.

Upon the completion of our qualitative exploration, we will transition to the Preparation Phase of EPIS. Drawing upon insights garnered from the Exploration Phase, we will curate evidence-based materials on HU, leveraging resources such as the American Society of Hematology (ASH) pocket guides for SCD management [32]. In the Preparation Phase, we will adapt the provider-level task-sharing implementation strategy. Our adaptive process ensures that the tailored TASSH TCP strategy aligns seamlessly with the identified context-specific needs and challenges. Through this iterative refinement, we can fine-tune our approach to maximize effectiveness and relevance within the unique landscape of Nigerian clinical sites. During this phase, we will meticulously chart out the training cycles for non-hematologist physicians and nurses across the 20 clinical sites. These sites encompass a spectrum of healthcare facilities, ranging from tertiary-level institutions like the University of Abuja Teaching Hospital to primary care facilities including Primary Healthcare Centers (PHC). We will conscientiously identify convenient timings for providers to undergo training, ensuring optimal participation, and subsequently develop a structured follow-up training schedule to sustain engagement and knowledge retention among participating providers.

During the Implementation Phase of EPIS, we will deploy our comprehensive training program to providers across 20 clinical sites participating in a cluster RCT. Within this phase, providers will be randomly assigned to one of two arms within the intervention trial. The first arm will comprise physicians and nurses who will undergo specialized training in the administration of HU. Additionally, these physicians and nurses will receive ASH pocket guides and will be supported by highly skilled nurses, also known as practice outreach facilitators (POFs), with extensive experience in both HU administration and SCD management at their local clinical sites. Furthermore, this intervention arm will benefit from periodic refresher courses on prescribing HU for SCD patients. This arm will encompass 7 sites, including PHCs and Teaching Hospitals. The second arm, designated as the Control Arm, will consist of physicians and nurses randomly allocated across 7 sites. In this arm, healthcare providers will receive initial training on HU administration and relevant information, such as ASH pocket guides. However, unlike the Intervention Arm, they will not receive ongoing support in the form of trained POFs. Throughout the study, investigators will evaluate the prescription behavior of healthcare workers in both arms at 0 and 12 months across all 14 clinical sites. This analysis aims to discern whether the prescription of HU has increased within either or both arms of the cluster RCT.

Moving to the Sustainment Phase, following 12 months of providing support to the intervention arm, we will withdraw clinical support and highly trained nurses/POFs from this group. Subsequently, we will assess the prescription of HU at the 24-month mark and assess utilization via patient-reported outcomes. Sustainment or maintenance will be determined by comparing the prescription rates of HU between the periods of 12–24 months and the initial baseline of 0–12 months across all 20 clinical sites.

This cluster RCT will provide insights into the efficacy of our educational intervention aimed at enhancing HU prescriptions for SCD patients. Success in improving provider-level adoption of HU is anticipated to facilitate better management of SCD, thus potentially improving patient outcomes.

### Study medication

HU is an international scientifically proven treatment for people living with SCD. HU is approved for the treatment of both adults and children with SCD. Hydroxyurea is also used, in larger doses, to treat cancer. HU has been shown to reduce vaso-occlusive crises, blood transfusions, hospitalizations, incidents of acute chest syndrome, improve organ function, and overall survival [33]. In Nigeria, HU is available in the following forms: Tablet Oxyurea® from Bond Chemical Industries and as a Capsule.

### Study setting and participants

Participants will be recruited from all eligible clinical sites from the Primary Health Care Centres that are part of the Consortium for Newborn Screening in Africa (CONSA) [34] in the Federal Capital Territory as well as tertiary hospitals that are part of the Sickle Pan African Research Consortium NigEria NEtwork (SPARC-NEt) in Nigeria. Participants will be recruited at their regularly scheduled clinical appointment or via telephone after a SPARC-NEt database search. The Nigerian healthcare system is a three-tiered pyramidal health structure in which the primary-level healthcare centers/facilities occupy the bottom tier, the district hospitals at the secondary level or middle tier, and the teaching hospitals at the tertiary level or top tier. We have targeted the district-level hospitals because it is where most SCD patients in Nigeria seek primary care. Additionally, sites that form the SPARC-NEt Nigeria network consisting of medical centers, teaching hospitals, and general hospitals/facilities will participate in this study. This study is approved by the Institutional Review Board of New York University, the Nigerian National Health Research and Ethics Committee, and the University of Abuja Ethics Committee, Abuja, Nigeria.

### Clinical site recruitment and randomization

We will recruit 20 clinical sites. Eligible clinical sites must have at least 2 Nurses employed and are able to participate in the SIM intervention (see Fig 2B). Participating sites will include newborn screening sites in the Federal Capital Territory under the Consortium for Newborn Screening (CONSA NBS) program as well as tertiary health care hospitals, which are part of SPARC-NEt with pre-consented patients enrolled in an electronic database. These sites have a demonstrated capacity to recruit >100 patients per site. The CONSA NBS program requires staff training to provide HU treatment as an important intervention. In the cluster randomized clinical trial, 20 sites will be randomized to the intervention arm or control arm. Randomization will occur at the clinical site level, with each facility randomized to the intervention arm (SIM+TASSH TCP) or control (educational information only on TASSH TCP). The cluster randomization sequence will be generated in accordance with CONSORT guidelines and overseen by our study statistician. Sites will be informed of their randomization group by email. Because of the nature of the intervention, it is impossible to blind the patients, healthcare providers, and the study coordinators to the group assignment.

For each clinical site receiving the SIM intervention (n = 10), a task force will be formed to support program implementation and it will include 1) leadership support (e.g., executive/clinical director), 2) performance feedback (provide feedback to nurses and physicians on using SIM, and 3) TCP strategy to support nurses and physicians in the delivery of care for SCD patients. All study sites are part of the CONSA and SPARC-NEt which is led by a hematologist and renowned SCD specialist. Having both PHC as well as tertiary hospitals in this study is strategic. This will build the capacity of healthcare workers in PHC to provide essential health services such as screening and treatment for non-communicable diseases, including SCD, in the communities close to where patients live in order to achieve the health-related SDG goals 3. Medical specialists such as cardiologists, nephrologists, orthopedic surgeons, and ophthalmologists are available in most centers forming part of the multidisciplinary team. Additionally, we will also leverage the expertise of specialists within SPARC-NEt in the management of SCD especially when end-organ complications occur. Other health workers such as pharmacists, medical records officers, counsellors, social workers, physiotherapists, nutritionists, data entry staff, and laboratory scientists are also involved in the management of the SCD and are members of SPARC-NEt.

Pharmacies at each clinical site will be provided with monthly questionnaires to assess HU utilization among patients and physician prescriptions during the study. SPARC-NEt has developed common protocols to leverage the expertise of various specialists within the consortium to support SCD management [35].

### Training and informed consent

The TASSH TCP (experimental arm) will receive training at baseline with booster training every 3 months for 1 year (12 months). The educational information only on TASSH TCP (control arm) will receive information only on task-sharing but not TASSH TCP training/facilitation. To eliminate cost as a potential confounder to HU access, all patients will be referred to a SPARC-NEt provider for HU medication and receive regular laboratory monitoring at no cost.

### Training for health care providers (SIM+TASSH TCP group)

We will conduct training for non-hematologist physicians and nurses on the SIM protocol for clinical sites randomized to the TASSH TCP arm. The workshops will assist trainees to achieve competencies in the study protocol, increase their knowledge of SCD and risk factors for SCD, laboratory monitoring, side effects of HU use, consensus-building, and goal setting for patients, problem-solving, performance assessment and feedback, and promoting teamwork. Only the sites randomized to the intervention arm will receive TASSH TCP from POFs who will provide in-person TASSH TCP to nurses and physicians of clinical sites randomized to this group at baseline, at periodic intervals, and monthly telephone support. Training effectiveness will be assessed via pre/post questionnaires before and after training to evaluate SCD management knowledge, the components of the SIM protocol, and its application at each bi-annual training.

### Training for health care providers (SIM only—No TASSH TCP group)

Baseline procedures and training of healthcare providers at participating clinical sites in the control group will be similar to those described above except this group will not receive TASSH TCP training or clinical support (i.e., POFs). Study nurses will receive training in proper procedures for obtaining informed consent from all study participants and study data collection. The study nurse coordinators are responsible for screening and assisting with data collection. All study staff involved in data collection and or data analyses will be trained in human subject protection before recruitment and data collection begin.

### Participant recruitment

We plan to recruit 900 participants across 20 clinical sites. Potential study participants will be approached by a nurse from each of the clinical sites during routine primary care visits with families. To account for attrition, each of the 20 sites will recruit 45–55 eligible patients for a total sample size of 900 who meet the following eligibility criteria over a 2-year period. Eligible study participants/parents, and caregivers will be consented and will complete study-related surveys at months 0, 6, 12, 18, 24.

### Inclusion criteria (Patients)

SCD patients 18 years older who have provided consent; Pediatric SCD patients aged 12 months to 17 years with an accompanying guardian and have provided informed consent or assent; registration in the electronic medical records (EMR) database with clinical charts and received care at the local clinical sites or health facilities and not on HU therapy; Hb Genotype: SCD-SS, SCD-Sβ° thal, SCD-SO_Arab_ (On a case by case basis, a severely affected person with SCD-SC may be offered HU therapy under a modified treatment protocol). The adult patient, parent, or legal guardian of the child patient must have demonstrated a high level of responsibility and must be regarded as capable of understanding the concepts of the therapy, following the treatment guidelines, and being willing to comply with the required visits, laboratory evaluations, and schedule of medications.

### Exclusion criteria (Patients)

Any SCD patient not registered in the EMR database; registered patients without informed consent or assent; physically unable to participate in study activities; an SCD patient on HU; or ongoing chronic transfusion therapy, chronic utilization of medications that may enhance toxicities of HU, concomitant chronic illness that has the potential to increase the toxicities of HU.

### Description of the intervention

Healthcare providers, including physicians and nurses, will undergo comprehensive training to acquire knowledge about HU and SCD. The training program is designed to be thorough yet efficient, with an initial didactic session expected to last for two hours. Subsequently, follow-up sessions will be conducted every three months throughout the first year of training. For physicians, a more in-depth portion of the training is anticipated, requiring approximately four hours to cover essential aspects comprehensively. This extended duration ensures that physicians receive detailed instruction and sufficient time for discussion and clarification on complex topics related to HU and SCD management. By investing in rigorous training sessions tailored to the specific needs of healthcare providers, we aim to equip them with the necessary knowledge and skills to effectively prescribe and manage HU therapy for patients with SCD, thereby enhancing overall patient care and outcomes.

For patients, no formal training will be necessary. Their interaction with healthcare providers will proceed as usual, with the added step of obtaining HU as prescribed. During each clinical visit, patients will engage in a brief question-and-answer session aimed at assessing their satisfaction with the provided service and their overall experience with HU therapy. This interactive session, designed to gather valuable feedback and ensure patient-centered care, is anticipated to last approximately one hour per visit.

### Provision of HU to patients in both study arms

Patients in both arms of the study will receive HU medications for the duration of the study at no cost to the patients, as well as laboratory monitoring (see *Laboratory monitoring after HU prescription section below)*. HU is on the list of essential medicines for Nigeria, and it is locally accessible. Providing HU to all patients will ensure that we address the medication access and affordability barrier for SCD management.

### SIM+TASSH TCP group (experimental arm)

All patients randomized to this arm will be provided HU medication for the length of the study at no cost to the patient; after the study ends, patients will be encouraged to keep their national health insurance to maintain HU access and to improve usage. The providers in this group will receive rigorous training as part of the TASSH TCP training, clinical support (i.e. POFs), and printed ASH HU pocket guides as clinical reminders tailored for the local context to facilitate SCD management [33]. Patients will receive HU during the regularly scheduled clinical visits with detailed instructions on how to take the medication, and advised on potential complications for which they should follow-up with the study provider. They will also receive SCD management educational materials. All assessments will occur during regularly scheduled clinical visits without the need for additional visits, thus reducing patient burden.

### SIM only—No TASSH TCP group (control arm)

Patients in this group will also receive HU medication and laboratory monitoring for the length of the study at no cost; after the study ends, patients will be encouraged to keep their national health insurance to maintain HU access and to improve usage. After the initial introduction to the program with initial training in the prescription of HU and the importance of laboratory monitoring at baseline, the healthcare providers in the control arm will not be exposed to implementation strategy [TASSH TCP] training during the intervention period but will be provided with all training materials. Furthermore, healthcare providers will not receive additional clinical support in the form of practice facilitation. Patients in this group will continue receiving usual care at their facilities and provided SCD management educational materials.

### Laboratory monitoring after HU prescription

Consensus treatment protocols for the implementation of HU therapy have been adapted [22, 36] and extensive laboratory monitoring protocols are available from the Sickle in Africa consortium, and Nigeria’s Federal Ministry of Health’s National Guideline for the Control and Management of Sickle Cell Disease [37], which is aligned with ASH guidelines. Patients on HU receive hematological tests for HbF induction and full blood count parameters to monitor drug effectiveness and potential side effects- this capacity currently exists within the SPARC-NEt Nigeria network. Laboratory monitoring will be provided to clinical sites as part of SPARC-NEt Nigeria at no cost to patients. Monitoring will be continued with patients enrolled in the National Health Insurance to allow for intervention sustainability after the end of funding which will eliminate a significant barrier to SCD management. Laboratory monitoring for HU will follow ASH protocols [32]. Patients will be encouraged to report medication side effects immediately. Health care providers will also assess patients for adverse events at each study visit. An Adverse Event is defined as any unwanted reaction, side effect, or other clinical event in which continued HU therapy might contribute to drug toxicity, danger to the patient, or the occurrence of any of the conditions listed in the exclusion criteria. Serious Adverse Event is defined as any life-threatening toxicity or medical event occurring during HU therapy whether related or not related to HU. Along with its needed effects, a medication may cause some unwanted effects that may require medical attention.

### Implementation outcome measures

The primary implementation outcome measure is the adoption rate of SIM at clinical sites at 12 months. Secondary implementation outcome is sustainability/maintenance of the SIM + TASSH TCP strategy from 12 to 24 months (maintenance of the adoption of SIM after the end of the implementation trial), and tertiary implementation outcome is implementation fidelity (the degree to which the program is delivered as intended which serves as an indicator of successful SIM adoption. Implementation Outcomes will be measured using an adoption, fidelity, and sustainability survey. We will attempt to collect implementation fidelity from all sites; however, adoption and sustainability/maintenance are the primary and secondary implementation outcomes of interest.

### Data management

An electronic data collection system is established in accordance with rigorous data management protocols of New York University and the University of Abuja. Electronic data collection forms are developed in a secured web-based electronic database capture system called Research Electronic Data Capture (REDCap) [38]. The forms have built-in data validation and consistency checks. Study data will be entered in REDCap; maintained and managed by the data management and analysis team at the University of Abuja, with the support of the Sickle Africa Data Coordinating Centre (SADaCC) in consultation with NYU. The data will be reviewed on a bi-monthly basis for inconsistencies and outliers. Issues will be communicated to the project coordinator to be addressed during the coordinators’ periodic visits to the data collection sites to ensure data quality.

### Sample size and power analysis

The unit of randomization for the intervention is the hospital or clinic; ten in the intervention arm and ten in the control arm (n = 20). Based on previous literature, implementing SIM to address SCD could have approximately 19% difference in the rates of composite measure of adoption between the treatment arms. Thus, we estimate sample sizes for a range of small differences (minimum detectable difference (MDD) between 7.5% to 10.5%, assuming standard deviation of 30% or Cohen’s d between 0.25 and 0.35) for this intervention, assuming an average class size (*m*) of about 45 participants/clinic, and intra-class coefficients (ICC)s between .01 and .06. This yields a total sample size of 20 x 45 = 900 participants, providing more than 80% power to detect a small difference of 9% with a conservative assumption of ICC = 0.02. Note that this includes adjustment for up to 30% attrition of the study sample; this estimate is conservative.

### Statistical methods

#### Analysis of primary implementation outcomes

The primary outcome measure is the rate of adoption of SIM at clinical sites at 12 months. This is a composite measure of adoption ratings to assess the degree to which the three essential elements of the SIM protocol are implemented at clinical sites. Thus, the primary outcome will be assessed at 12 months by the following measures: 1) the number of patients taking HU identified through screening, 2) the proportion of patients on HU, and 3) the proportion of patients who maintained dosage. We hypothesize that the level of SIM adoption will be higher in the clinical sites randomized to the experimental condition than those in the control arm.

Ideally, the randomization of participants to treatment groups will obviate the need for any covariates in the analysis. However, in the event that baseline differences between the patients in each group on the outcomes, demographic, or secondary measures are found, those variables will be included as covariates in the MANOVA (including their interactions with time).

#### Analysis of secondary implementation outcomes

Secondary outcome measure is the sustainability/maintenance of the intervention across clinical sites from 12 to 24 months. Sustainability is based on the constructs of the EPIS, including inner context characteristics of the clinics, intervention characteristics, and implementation process measures. In brief, the hypothesis is that the sustainability of SIM will be higher in the sites randomized to SIM+TASSH TCP (experimental arm) compared to those in control condition (no TASSH TCP). We will evaluate the factors that mediate the effect of TASSH TCP implementation strategy on sustainability and assess the extent to which inner setting variables (e.g., implementation leadership, implementation climate, and organizational culture) affect the degree of adoption of SIM and its sustainability at 24 months. Sustainability can be measured as the number of prescriptions for HU from months 0–12 compared to 12-24months and will include the other areas of the SIM intervention. Furthermore, we will pay particular attention to the pathways via which these variables influence the association of intervention and adoption and sustainability outcomes. We will estimate a just-identified path model using the robust weighted least squares estimator to investigate relationships among the theoretical mediators of implementation climate, implementation leadership, organizational culture, organizational readiness to change, and external change agent support. Based on the conceptual model, we will test the direct effects from the theoretical constructs on the adoption components (individually). In addition to the direct effects, the indirect effects from each variable to adoption via inner setting variables will be estimated as the product of component direct effects and tested using bootstrapped 95% confidence intervals. Finally, we will estimate the direct effects of the predicted model of adoption on SCD outcomes. Predicted probabilities of the adoption and sustainability outcomes will be calculated from path model coefficients to elucidate the magnitudes of direct and indirect effects.

#### Analysis of tertiary implementation outcomes

The tertiary implementation outcome is implementation fidelity (degree to which the program is delivered as intended), which serves as an indicator of successful SIM adoption. Implementation fidelity will be measured using a fidelity checklist which captures adherence to study intervention components and level of implementation. This will occur following TASSH TCP training and patient encounters.

### Trial status

The study was funded in August 2023, and we are preparing to embark on the Exploration and Preparation (pre-implementation) phases. All key stakeholders have been identified and ethical approvals have been obtained from the National Health Research and Ethics committee, the University of Abuja in Nigeria, and New York University.

## Discussion

Training non-hematologist physicians and nurses in the appropriate use and management of HU for SCD can significantly enhance prescription practices and ensure fidelity in SCD management at healthcare facilities. This is achieved through evidence-based educational training supplemented with clinical support via practice facilitation. Given the limited number of hematologists in Nigeria, it is imperative to implement educational interventions alongside various implementation strategies to increase HU adoption at clinical sites. There is an urgent need and an opportunity to build local capacity to implement these strategies, thereby improving SCD outcomes.

The ACCELERATE study represents a groundbreaking initiative set to revolutionize SCD management in Abuja, Nigeria and the surrounding areas. By focusing on EBP, this endeavor aims to transcend traditional clinical approaches by introducing innovative strategies to optimize patient care and treatment outcomes. At the heart of the SIM+TASSH TCP intervention lies a meticulously crafted practice centered on training healthcare providers in HU administration for SCD management. This rigorously evaluated intervention serves as a cornerstone of the study’s holistic approach to addressing the complex challenges of SCD management in Nigeria. By equipping healthcare professionals with the necessary knowledge and skills to deliver HU-based treatment regimens effectively, we aim to empower them to provide enhanced care and support, thereby improving health outcomes and quality of life for individuals with SCD.

Furthermore, ACCELERATE offers a unique opportunity to bridge the gap between research and practice by evaluating the effectiveness of our real-world implementation strategy for HU, drawing on clinical evidence from previously NHLBI-funded clinical trials. This evaluation will address the current lack of adoption of evidence-based interventions for SCD management and assess its impact across clinical sites in Nigeria, serving over 9,000 SCD patients. While TASSH implementation strategies have demonstrated sustainability and effectiveness in high-income countries and LMICs, our specific SCD-focused TASSH TCP approach is yet to be widely applied in LMICs, especially for SCD management. The proposed evidence-based implementation strategy aims to effectively overcome these barriers. The Practice Facilitation strategy embedded in our TASSH-TCP approach provides external practice redesign expertise and a tailored approach to implementing guideline-concordant care, further enhancing the effectiveness of our intervention.

## Conclusions

Nigeria and other SSA countries bear a heavy burden of SCD, yet the adoption of EBI/P for its management remains alarmingly low. Patient-level factors, such as the financial strain and the necessity for regular laboratory monitoring, pose significant obstacles to the widespread use of HU as an EBI to combat SCD in Africa. At the provider level, barriers to HU prescription persist, including insufficient formal training for healthcare providers, a lack of clinical reminders, and the absence of performance feedback mechanisms to enhance SCD management in Nigeria. These challenges are compounded by the scarcity of hematologists in the region. Furthermore, critical aspects such as HU acceptability, fidelity, delivery context/platform, and adoption have not undergone rigorous assessment.

In response to these pressing concerns, our study will employ the SIM intervention alongside a task-sharing strategy (TASSH-TCP) to bolster the capacity of SCD providers for HU adoption within the Nigerian context. By addressing both patient and provider-level barriers, we aim to facilitate the widespread acceptance and implementation of HU therapy, thus significantly enhancing SCD management practices.

The outcomes of this study will furnish healthcare providers, policymakers, and stakeholders with invaluable insights into effective strategies for SCD management, leveraging the existing infrastructure of SCD clinical networks and resources in Nigeria. Through a scalable and sustainable approach, we endeavor to improve the adoption of HU among providers across the nation. Moreover, this research will contribute to the establishment of best practices for implementing HU therapy in resource-constrained settings, thereby optimizing patient outcomes and advancing the global fight against SCD.

## Supporting information

S1 Checklist ACCELERATE SPIRIT checklist. (PDF)

S1 File ACCELERATE award letter. (PDF)

S2 File IRB-NYU. (PDF)

S3 File IRB-Nigeria national. (PDF)

S4 File IRB-UATH. (PDF)

S5 File ACCELERATE protocol. (PDF)

## Abbreviations

HU: Hydroxyurea
CESRTA: The Centre of Excellence for Sickle Cell Disease Research and Training, University of Abuja
SCD: Sickle Cell Disease
Hb: hemoglobin
HbF: Fetal hemoglobin
REACH: Realizing Effectiveness Across Continents with Hydroxyurea
TASSH: TAsk-Strengthening Strategy for Hemoglobinopathies
TCP: Training healthcare workers and providers to be more patient-centered in clinical consultations + ii) Clinical reminders + iii) Practice facilitation
EPIS: Exploration, Preparation, Implementation, and Sustainment
SSA: Sub-Saharan Africa
ACS: Acute Chest Syndrome
EBP: evidence-based practice
SPARCO: Sickle Pan African Research Consortium
SCSSN: Sickle Cell Support Society of Nigeria
SPARC-NEt: Sickle Pan African Research Consortium—Nigeria
SIM: Screening, Initiation, and Maintenance
SADaCC: Sickle Africa Data Coordinating Centre
LMIC: low- and middle-income country
MDD: minimum detectable difference
ICC: intra-class coefficients
CONSA: Consortium for Newborn Screening in Africa
SDG: Sustainable Development Goal
EMR: electronic medical records
ARC: absolute reticulocyte count
ANC: absolute neutrophil count
CBC: Complete Blood Count
CML: Chronic Myeloid Leukemia
OSMB: Observational Safety and Monitoring Board
REDCap: Research Electronic Data Capture
FIML: full information maximum likelihood
