# Supplementary material for: An implementation trial to mAnage siCkle CELl disEase through incReased AdopTion of hydroxyurEa in Nigeria (ACCELERATE): Study protocol
Source: PLoS One. 2025 Jan 8;20(1):e0311900. doi: 10.1371/journal.pone.0311900 (PMC11709263; doi:10.1371/journal.pone.0311900)
Supplement: S1 File — (PDF) [file pone.0311900.s002.pdf]

Notice of Award

RESEARCH PROJECT COOPERATIVE AGREEMENT
Department of Health and Human Services
National Institutes of Health

NATIONAL HEART, LUNG, AND BLOOD INSTITUTE

SECTION I – AWARD DATA – 1U01HL168084-01

Principal Investigator(s):
Obiageli Eunice Nnodu, MS
EMMANUEL K PEPRAH (contact), PHD

Award e-mailed to: osp.agency@nyu.edu

Dear Authorized Official:

The National Institutes of Health hereby awards a grant in the amount of $708,422 (see "Award Calculation" in Section I and "Terms and Conditions" in Section III) to NEW YORK UNIVERSITY in support of the above referenced project. This award is pursuant to the authority of 42 USC 241 31 USC 6305 42 CFR 52 and is subject to the requirements of this statute and regulation and of other referenced, incorporated or attached terms and conditions.

Acceptance of this award, including the "Terms and Conditions," is acknowledged by the recipient when funds are drawn down or otherwise requested from the grant payment system.

Each publication, press release, or other document about research supported by an NIH award must include an acknowledgment of NIH award support and a disclaimer such as "Research reported in this publication was supported by the National Heart, Lung, And Blood Institute of the National Institutes of Health under Award Number U01HL168084. The content is solely the responsibility of the authors and does not necessarily represent the official views of the National Institutes of Health." Prior to issuing a press release concerning the outcome of this research, please notify the NIH awarding IC in advance to allow for coordination.

Award recipients must promote objectivity in research by establishing standards that provide a reasonable expectation that the design, conduct and reporting of research funded under NIH awards will be free from bias resulting from an Investigator's Financial Conflict of Interest (FCOI), in accordance with the 2011 revised regulation at 42 CFR Part 50 Subpart F. The Institution shall submit all FCOI reports to the NIH through the eRA Commons FCOI Module. The regulation does not apply to Phase I Small Business Innovative Research (SBIR) and Small Business Technology Transfer (STTR) awards. Consult the NIH website http://grants.nih.gov/grants/policy/coi/ for a link to the regulation and additional important information.

If you have any questions about this award, please direct questions to the Federal Agency contacts.

Sincerely yours,

Ronald Caulder
Grants Management Officer
NATIONAL HEART, LUNG, AND BLOOD INSTITUTE

Additional information follows
