# Supplementary material for: An implementation trial to mAnage siCkle CELl disEase through incReased AdopTion of hydroxyurEa in Nigeria (ACCELERATE): Study protocol
Source: PLoS One. 2025 Jan 8;20(1):e0311900. doi: 10.1371/journal.pone.0311900 (PMC11709263; doi:10.1371/journal.pone.0311900)
Supplement: S2 File — (PDF) [file pone.0311900.s003.pdf]

NYU Langone Health

Office of Science and Research
Institutional Review Board
1 Park Avenue | 6th Floor | New York, NY 10016

Approval of Submission

May 31, 2024

On 5/31/2024 the IRB reviewed and approved the following submission:

principal investigator	Olugbenga Ogedegbe
study number	i24-00437
study title	mAnaging siCkle CELl disEase through incReased AdopTion of hydroxyurEa in Nigeria (ACCELERATE)
performance period	5/28/2024 - 5/27/2025
location(s)	180 Madison Avenue (NYUMC Locations)
sponsor(s)	Name: NATIONAL HEART, LUNG, AND BLOOD INSTITUTE (NHLBI)
review type	Initial Study
board name	Board C (NYU Grossman SoM)
materials approved for use	• Provider_Policymaker Focus Group Form_ACCELERATE_FINALv3.2.24.pdf, Category: Recruitment Materials;<br>• template-assent-non-bio_7-11.pdf, Category: Consent Form;<br>• ACCELERATE STUDY FORMS_FINAL .docx, Category: IRB Protocol;<br>• key-information-template-2019 _Patient Intervention_5.3.24.pdf, Category: Consent Form;<br>• template-assent-15-17.pdf, Category: Consent Form;<br>• Patient Focus Group Form_ ACCELERATE_FINALv3.2.24.pdf, Category: Recruitment Materials;<br>• apdx-intl-research 5.3.24_tracked changes.docx, Category: IRB Protocol;<br>• FOCUS GROUP ICF_5.28.24_IRB edits_jg .pdf, Category: Consent Form;<br>• template-assent-12-14-non-bio _ tracked changes_5.3.24.pdf, Category: Consent Form;<br>• ACCELERATE Focus Group Discussion Guide for pts_providers_pol_jg2.22.24.docx, Category: IRB Protocol;<br>• National IRB Approval for ACCELERATE(Nigeria).pdf, Category: Other;<br>• 2024.04.05 Apdx Children.docx, Category: IRB Protocol;<br>• UATH IRB Ethical Approval.pdf, Category: Other;<br>• template-consent-audio-video_5.3.24.pdf, Category: Consent Form;<br>• ACCELERATE Recruitment Flyer_PATIENTS_V1_Revised.pdf, Category: Recruitment Materials;<br>• ACCELERATE Recruitment Flyer_HC PROVIDERS_v1.pdf, Category: Recruitment Materials;<br>• PATIENT ICF_5.28.24_IRB edits_jg.pdf, Category: Consent Form;<br>• PROTOCOL_comments_ep V3.5.24.24.docx, Category: IRB Protocol;<br>• key-information-template-2019 _ALL Participants_Focus Group_5.3.24.pdf, Category: Consent Form;

31-MAY-2024

Link to Study	Open study in web browser

The current IRB Status of your submission is: Approved.

This submission was reviewed by the NYU Langone Health Institutional Review Board (IRB) referenced above. During the review of your study, the IRB specifically considered:

1. the risks and anticipated benefits (if any) to your subjects
2. the selection of subjects
3. the procedures for securing and documenting informed consent
4. the safety of your subjects
5. the privacy of your subjects and confidentiality of the data

Your study cannot commence until all ancillary review decisions are complete. To determine the state of all ancillary reviews, go the MyStudies page of this study in Research Navigator. Ancillary review statuses are located on the top/right area of your study's main screen.

Note: Ensure that approval has been issued in MyAgreements/CRMS and the Clinical Research Support Unit (CRSU) before you proceed with any aspect of this study, including the enrollment of human subjects.

Note: Financial Disclosures for all study team members must be kept up to date throughout the study. Should the Conflict of Interest Management Unit (CIMU) issue a new or revised financial conflict of interest management decision that effects any IRB approved study documents associated with this study, the Principal Investigator of this study is responsible for making sure the revised documents reflecting the CIMU decision are submitted to the IRB for review and approval via Modification. The Modification should include all revised documents, as applicable.

For questions regarding this submission, please contact ***Olga Shulyha.***

Thank you for using the IRB at NYU Langone Health.

Review Notes

For NIH grant funded research approved before the revised Common Rule: the IRB has found the IRB approved protocol referenced above to be consistent with the NIH grant application.

This study involves a vulnerable population: children. In accordance with 45 CFR 46.401 and NYU Policy the IRB has determined that this research involves minimal risk (45 CFR 46.404). Additionally, the IRB found the research includes adequate provision for soliciting the assent of the child and the permission of one parent(s) or guardian.

This approval letter serves as the approval for the abovementioned location(s) only. If your study is a multi-site study using NYU SoM IRB as the Single IRB, this approval letter does not serve as approval for participating sites. If your study is a multi-site study not requiring the use of a Single IRB, this approval does not serve as approval for any of the other sites. Each site will need to obtain IRB approval before human subjects research may commence at the site.

31-MAY-2024

NYU Langone Health

Office of Science and Research
Institutional Review Board
1 Park Avenue | 6th Floor | New York, NY 10016

NYU Grossman School of Medicine Federal wide Assurance: FWA00004952
NYU Winthrop/Long Island School of Medicine Federal wide Assurance: FWA00000726

NYU Langone Health operates in accordance with Good Clinical Practices (GCP) and applicable laws and regulations. Federal rules allow IRB to document their determination/authorization process in their policy manual. Determination letters generated by NYU Langone Health IRB administration system are not physically signed as per policy. All approved study materials are clearly identified and locked in each study submission record within the IRB's administration system.

NYU Langone Health IRB Policy

- *All current IRB policy documents can be found on our website*
- *You must submit all modifications to this study (e.g., protocol updates, modified recruitment materials, consent forms, etc.) using Research Navigator to communicate with the IRB ("eSubmission") for review and approval prior to initiation of those change(s), except where necessary to eliminate apparent immediate hazards to the subject(s). Changes made to eliminate apparent immediate hazards to subjects must be reported to the IRB within 24 hours.*
- *All adverse and/or unanticipated event(s) that occur while conducting this study must immediately be reported to the IRB via* eSubmission.
- *You may only use IRB-approved copies of your consent form(s), questionnaire(s), letter(s), advertisement(s), etc. in your study. Never use expired consent forms.*
- *If modifications are made to the study or adverse events occur while conducting study, the PI must inform all research staff listed on this study.*
- *IRB's approval is valid as per the period indicated above. A reminder to submit a continuation (should one be required) will be e-mailed to the PI, PI Proxy and Primary Contact 90, 60 and 30 days prior to this study's expiration date if one is indicated. After expiration, a daily reminder will be sent for 30 days followed by a weekly reminder until the study receives re-approval or a study closure.*
- *Prior to initiating an IRB-approved study, you must receive written approval from an authorized representative for each site where your study will take place. Key contacts are:*
  - *Bellevue Hospital (BHC): if you are conducting all or part of your study at BHC, you must contact them to obtain additional approvals. BHC will be notified if any of their sites are selected as a location where your study takes place, but your team is obligated to contact them at bellevueresearch@nychhc.org to find out what approvals are required before conducting any research at a BHC location.*
  - *CTSI - Clinical and Translational Science Institute, NYU School of Medicine [formerly General Clinical Research Center (GCRC)]: email ctsi@nyulangone.org*
  - *NYU Langone Health Centers (Tisch Hospital/Rusk Institute/Co-op Care/HJD/Perlmutter Cancer Center) site approval is handled for you automatically (as needed) by the CRSU*
- *The IRB may suspend or terminate studies that are not in compliance with NYU Langone Health IRBs Policies & Procedures and the requirements of the Institution's Federal Wide Assurance with the federal government.*
- *Direct IRB questions and comments to 212-263-4110 or IRB-INFO@nyulangone.org*

Let Us Know How We're Doing

Click on the title above to send us feedback via a short, anonymous survey. Providing exceptional customer service is a top priority of the IRB and your responses will help us understand how we can continue to improve our service to the research community.

IRB Board Rosters

Effective **2024-05-01** FWA#00004952
Quorum is simple majority only = greater than half

31-MAY-2024

Board A (Effective 2024-05-01)

Members; Quorum of 7; 8 for prisoner-related studies

Member	Degree(s)	Science – Non-Science	Specialty	Affiliated With NYU	Alternate(s)
More, Frederick (Chair)	DDS	S	Epidemiology & Health Promotion Pediatrics	Y	Goddard, Hazen, Katz, Nishawala, Novik, Quinn, Trinh
Ayoubai, Doaa	PharmD, PhD	S	Pharmacy	Y	Correll, De los Reyes, D'Onofrio, Dubrovskaya, Vasile
Basu Roy, Upal	PhD, MPH	S	Community Representative	N	Bee, Raskin, Wu (Lillian)
Donnino, Robert (Vice Chair)	MD	S	Medicine – Cardiology	Y	Vice Chair Alternates: Hazen, Nishawala,<br><br>Member Alternates: Katz, Kerwin, Lachmann
Davies, Faith	MD	S	Oncology	Y	D'Abreo, Garrison, Kwa, Marks, Novik, O'Keeffe, Raince, Ryan, Tegla, Schiff, Wu
Diefenbach, Catherine	MD	S	Oncology	Y	D'Abreo, Garrison, Kwa, Marks, Novik, O'Keeffe, Raince, Ryan, Tegla, Schiff, Wu
Gallagher, Richard	PhD	S	Child & Adolescent Psychiatry	Y	Chervinsky, Frankle, Nishawala, Schultebraucks
Godina, Marina	RN	S	IRB Administration; Nursing	Y	Berkovitz, Jeschke-Lopez, Johnson, Joseph
Kadidal, Shane *	JD	NS	Law – Prisoner Advocate	N	
Maril, Robert	CIP	NS	IRB Administration	Y	Decker, Mann, McGowan, Mosby, Nicolas, Panageas, Pogorelec-Khan, Vieira
Meftah, Morteza	MD	S	Orthopedic Surgery	Y	Aggarwal, Kirsch, Petrone, Prescott
Ross, Stephen	MD	S	Psychiatry	Y	Bogenschutz, Chervinsky, Frankle, Nishawala, Schultebraucks
Storey, Elizabeth	PhD	S	Radiology	Y	Fieremans, Ge, Kirov, Lazar, Rusinek
Wang, Jing	MD	S	Anesthesiology	Y	Kim

Counts toward quorum only when reviewing studies subject to Subpart C

Board B (Effective 2024-05-01)

Members; Quorum of 9

Member	Degree(s)	Science – Non-Science	Specialty	Affiliated With NYU	Alternate(s)
Katz, Stuart (Chairman)	MD PhD	S	Cardiology	Y	Donnino, Goddard, Hazen, More, Nishawala, Novik, Quinn, Trinh
Hazen, Alexes (Vice Chair)	MD	S	Surgery – Plastic	Y	Vice Chair Alternates: Donnino, More, Nishawala, Novik, Petrone
Brar, Preneet	MD	S	Pediatric Endocrinology	Y	Ramirez,

Dubrovskaya, Yanina	PharmD	S	Pharmacy	Y	Ayoubai, Correll, De los Reyes, D'Onofrio, Vasile
Ge, Yulin	MD	S	Radiology	Y	Fieremans, Kirov, Lazar, Rusinek, Storey
Godina, Marina	RN	S	IRB Administration; Nursing	Y	Berkovitz, Jeschke-Lopez, Johnson, Joseph
Grossman, Scott	MD	S	Neurology	Y	Leeman-Markowski, Ross-Rizzo
Kadidal, Shane *	JD	NS	Law-Prisoner Advocate	N	
Kirsch, Thorsten	PhD	S	Orthopedic Surgery	Y	Aggarwal, Meftah, Petrone, Prescott
Kwa, Maryann	MD	S	Oncology	Y	D'Abreo, Davies, Diefenbach, Garrison, Marks, Novik, O'Keeffe, Raince, Ryan, Tegla, Schiff, Wu
Lachmann, Justine	MD	S	Cardiology	Y	Donnino, Giannarelli, Katz, Kerwin
Maril, Robert	CIP	NS	IRB Administration	Y	Decker, Mann, McGowan, Mosby, Nicolas, Panageas, Pogorelec-Khan, Vieira
McGowan, Richard	MLS	NS	Public Services	Y	Nicolas, Vieira
Nolan, Anna	MD	S	Pulmonary	Y	
Raskin, Joyce	JD	NS	Community Representative	N	Basu Roy, Bee, Wu(Lillian)
Schultebraucks, Katharina	PhD	S	Psychiatry	Y	Bogenschutz, Chervinsky, Frankle, Gallagher, Nishawala, Ross
Silverman, Joshua	MD, PhD	S	Radiation Oncology	Y	D'Abreo, Davies, Diefenbach, Garrison, Kwa, Marks, Novik, O'Keeffe, Raince, Ryan, Tegla, Schiff, Wu

**Counts toward quorum only when reviewing studies subject to Subpart C*

Board C (Effective 2024-05-01)

Members; Quorum of 8

Member	Degree(s)	Science – Non-Science	Specialty	Affiliated With NYU	Alternate(s)
Novik, Yelena (Chair)	MD	S	Oncology	Y	Donnino, Goddard, Hazen, Katz, More, Nishawala, Quinn, Trinh

31-MAY-2024

Nishawala, Melissa (Vice Chair)	MD	S	Child & Adolescent Psychiatry	Y	Vice Chair Alternates: Donnino, Hazen, Katz, More<br><br>Member Alternates: Bogenschutz, Chervinsky, Frankle, Gallagher, Ross, Schultebraucks
Chervinsky, Alexander B	PhD	S	Neuropsychology	Y	Gallagher, Nishawala, Ross, Schultebraucks
D'Onofrio, Nicole	PharmD, RPh	S	Pharmacy	Y	Ayoubai, Correll, De los Reyes, Dubrovskaya, Vasile
Godina, Marina	RN	S	IRB Administration; Nursing	Y	Berkovitz, Jeschke-Lopez, Johnson, Joseph
Giannarelli, Chiara	MD	S	Cardiology	Y	Donnino, Katz, Kerwin, Lachmann
Kadidal, Shane *	JD	NS	Law – Prisoner Advocate	N	
Kim, Sunmi	MD	S	Anesthesiology	Y	Wang
Maril, Robert	CIP	NS	IRB Administration	Y	Decker, Mann, McGowan, Mosby, Nicolas, Panageas, Pogorelec-Khan, Vieira
Nippita, Siripanth	MD	S	OB/GYN	Y	Kuokkanen, Mehta-Lee, Shanahan, Wissner-Greene
Prescott, Jason	MD	S	Endocrine Surgery	Y	Aggarwal, Kirsch, Meftah, Petrone
Schiff, Peter	MD	S	Radiation Oncology	Y	D'Abreo, Davies, Diefenbach, Garrison, Kwa, Marks, O'Keeffe, Raince, Ryan, Tegla, Silverman
Vieira, Dorice	MA	NS	Public Services	Y	Nicolas, McGowan
Wu, Jennifer	MD	S	Oncology	Y	D'Abreo, Davies, Diefenbach, Garrison, Kwa, Marks, O'Keeffe, Raince, Ryan, Tegla, Silverman
Wu, Lillian	BA, MA	NS	Community Representative	N	Basu Roy, Bee, Raskin

**Counts toward quorum only when reviewing studies subject to Subpart C*

31-MAY-2024

Board D (Effective 2024-05-01)

Members; Quorum of 7

Member	Degree(s)	Science – Non-Science	Specialty	Affiliated With NYU	Alternate(s)
Trinh, Chau (Chair)	DrPH	S	Population Health	Y	Donnino, Goddard, Hazen, Katz, More, Nishawala, Novik, Quinn
Dedania, Vaidehi (Vice-Chair)	MD	S	Ophthalmology	Y	
Aggarwal, Vinay	MD	S	Orthopedic Surgery	Y	Kirsch, Meftah, Petrone, Prescott
De Los Reyes, Francis	PharmD	S	Pharmacy	Y	Ayoubai, Correll, Dubrovskaya, D'Onofrio, Vasile
Godina, Marina	RN	S	IRB Administration; Nursing	Y	Berkovitz, Jeschke-Lopez, Johnson, Joseph
Kadidal, Shane *	JD	NS	Legal – Prisoner Advocate	N	
Kirov, Ivan	PhD	S	Radiology	Y	Ge, Fieremans, Lazar, Rusinek, Storey
Leeman-Markowski, Beth	MD	S	Neurology	Y	Grossman, Ross-Rizzo
Maril, Robert	CIP	NS	IRB Administration	Y	Decker, Mann, McGowan, Mosby, Nicolas, Panageas, Pogorelec-Khan, Vieira
Mehta-Lee, Shilpi	MD	S	Ob-Gyn	Y	Kuokkanen, Nippita, Shanahan, Wissner-Greene
Punekar, Salman	MD	S	Oncology	Y	D'Abreo, Davies, Diefenbach, Kwa, Novik, Marks, O'Keeffe, Raince, Ryan, Tegla, Schiff, Wu
Ramirez, Michelle	MD	S	Pediatric Critical Care	Y	Brar, Oshva
Tegla, Cosmin	MD	S	Oncology	Y	D'Abreo, Davies, Diefenbach, Garrison, Kwa, Novik, Marks, O'Keeffe, Raince, Ryan, Schiff, Wu

**Counts toward quorum only when reviewing studies subject to Subpart C*

31-MAY-2024

Board E (Effective 2024-05-01)

Members; Quorum of 4; 5 for prisoner-related studies

Member	Degree(s)	Science – Non-Science	Specialty	Affiliated With NYU	Alternate(s)
More, Frederick (Chair)	DDS	S	Dentistry	Y	Donnino, Goddard, Hazen, Katz, Nishawala, Novik, Quinn, Trinh
Katz, Stuart (Vice-Chair)	MD	S	Cardiology	Y	Vice Chair Alternates: Dedania, Donnino, Hazen, Marciano, More, Nishawala, Novik, Trinh<br>Member Alternates: Donnino, Giannarelli, Kerwin, Lachmann
Basu Roy, Upal	PhD, MPH	S	Community Representative	N	Bee, Raskin, Wu(Lillian)
Gallagher, Richard	PhD	S	Child & Adolescent Psychiatry	Y	Aggarwal, Bogenschutz, Brar, Chervinsky, Davies, Diefenbach, Donnino, Fieremans , Frankle, Ge, Hazen, Kim, Kirsch, Kwa, Nishawala, Nolan, Novik, Ramirez, Ross, Ross-Rizzo, Ryan, Tegla, Schiff, Schultebraucks, Storey, Wang, Wu
Goddard, David H	MD	S	Rheumatology	Y	Aggarwal, Bogenschutz, Brar, Chervinsky, Davies, Diefenbach, Donnino, Fieremans, Frankle, Ge, Hazen, Kim, Kirsch, Kirov, Kwa, Leeman-Markowski, Nishawala, Nolan, Novik, Ramirez, Ross, Ross-Rizzo, Ryan, Tegla, Schiff, Nippita, Shanahan, Storey, Wang, Wu
Godina, Marina	RN	S	IRB Administration	Y	Berkovitz, Jeschke-Lopez, Johnson, Joseph
Kadidal, Shane *	JD	NS	Law-Prisoner Advocate	N	
Panageas, Helen	BA	NS	IRB Administration	Y	Decker, Mosby, Liu, Mann, Maril, McGowan, Nicolas, Pogorelec-Khan, Vieira

**Counts toward quorum only when reviewing studies subject to Subpart C*

31-MAY-2024

NYU Langone Health

Office of Science and Research
Institutional Review Board
1 Park Avenue | 6th Floor | New York, NY 10016

Board F (Effective 2024-05-01)

FWA# 00000726

Members; Quorum of 6, 7 for Prisoner Studies

Member	Degree(s)	Science – Non-Science	Specialty	Affiliated With NYU	Alternate(s)
Goddard, David (Chair)	MD	S	Rheumatology	Y	Donnino, Hazen, Katz, Marciano, More, Nishawala, Novik, Quinn, Trinh
Marciano, Tuvia (Vice Chair)	MD	S	GI/ Pediatrics	Y	Vice Chair Alternates: Donnino, Hazen, Nishawala<br><br>Member Alternate: Poppers
Correll, April	PharmD	S	Pharmacy	Y	Ayoubai, De los Reyes, Dubrovskaya, D'Onofrio, Vasile,
Bee, Peter	JD	NS	Community Representative	N	Basu Roy, Raskin, Wu(Lillian)
Dutka, Paula	BSN, MSN	S	Nephrology – Nursing Representative	Y	Godina, Joseph
Garrison, Michael	MD	S	Oncology/Hematology	Y	D'Abreo, Davies, Diefenbach, , Kwa, Novik, Marks, O'Keeffe, Raince, Ryan, Tegla, Schiff, Silverman, Wu
Kadidal, Shane *	JD	NS	Law – Prisoner Advocate	N	
Kerwin, Todd	MD	S	Cardiology	Y	Donnino, Giannarelli, Katz, Lachmann
Lazar, Mariana	PhD	S	Radiology	Y	Fieremans, Ge, Kirov, Rusinek, Storey
Maril, Robert	CIP	NS	IRB Administration	Y	Decker, Mann, McGowan, Mosby, Nicolas, Panageas, Pogorelec-Khan, Vieira
Petrone, Patrizio	MD	S	Surgery	Y	Aggarwal, Hazen, Kirsch, Meftah, Prescott
Rajan, Anand	MPH	S	Biostatistician	Y	Islam

Counts toward quorum only when reviewing studies subject to Subpart C

31-MAY-2024

Office of Science and Research
Institutional Review Board
1 Park Avenue | 6th Floor | New York, NY 10016

Board G (Effective 2024-05-01)

FWA# 00000726

Members; Quorum of 7, 8 for prisoner studies

Member	Degree(s)	Science – Non-Science	Specialty	Affiliated With NYU	Alternate(s)
Quinn, Gwendolyn (Chair)	PhD	S	OB/GYN	Y	Donnino, Goddard, Hazen, Katz, More, Nishawala, Novik, Trinh
Bee, Peter	JD	NS	Community Representative	N	Basu Roy, Raskin, Wu(Lillian)
D'Abreo, Nina	MD	S	Oncology	Y	Davies, Diefenbach, Garrison, Kwa, Novik, O'Keeffe, Raince, Ryan, Tegla, Schiff, Wu
Godina, Marina	RN	S	IRB Administration – Nursing Representative	Y	Berkovitz, Dutka, Jeschke-Lopez, Johnson, Joseph
Islam, Shahidul	DrPH, PStat®	S	Biostatistics	Y	Rajan
Kadidal, Shane *	JD	NS	Law – Prisoner Advocate	N	
Kuokkanen, Satu	MD, PhD	S	OB/GYN	Y	Mehta-Lee, Nippita, Wissner-Greene
Maril, Robert	CIP	NS	IRB Administration	Y	Decker, Mann, McGowan, Mosby, Panageas, Pogorelec-Khan, Vieira
Marks, Doug	MD	S	Oncology	Y	Davies, Diefenbach, Garrison, Kwa, Novik, O'Keeffe, Raince, Ryan, Tegla, Schiff, Wu
Nicolas, Barnaby	MSIS, AHIP	NS	Non-Scientist	Y	McGowan, Vieira
Poppers, David	MD	S	Gastroenterology	Y	Marciano
Rusinek, Henry	PhD	S	Radiology	Y	Fieremans, Ge, Kirov, Lazar, Storey
Shanahan, Lisa	DO	S	OB-GYN	Y	Mehta-Lee, Nippita, Wissner-Greene
Vasile, Alicia	RPh	S	Pharmacy	Y	Ayoubai, Correll, De los Reyes, D'Onofrio, Dubrovskaya

Alternates

Alternate	Degree(s)	Science – Non-Science	Specialty	Affiliated With NYU	Alternate For
Aggarwal, Vinay	MD	S	Orthopedic Surgery	Y	Kirsch, Meftah. Petrone, Prescott **Board E only Goddard**
Ayoubai, Doaa	PharmD, PhD	S	Pharmacy	Y	Correll, De los Reyes, D'Onofrio, Dubrovskaya, Vasile
Basu Roy, Upal	PhD, MPH	S	Community Representative	N	Raskin, Wu (Lillian)
Bee, Peter	JD	NS	Community Representative	N	Basu Roy, Raskin, Wu (Lillian)
Berkovitz, David	MD	S	IRB Administration	Y	Godina
Bogenschutz, Michael	MD	S	Psychiatry	Y	Chervinsky, Frankle, Gallagher, Nishawala, Ross, Schultebraucks**, Board E only Goddard**
Brar, Preneet	MD	S	Pediatric Endocrinology	Y	Ramirez, **Board E only Goddard**
Chervinsky, Alexander	PhD	S	Neuropsychology	Y	Gallagher, Nishawala, Ross, Schultebraucks
Correll, April	PharmD	S	Pharmacy	Y	Ayoubai, De los Reyes, D'Onofrio, Dubrovskaya, Vasile
Davies, Faith	MD	S	Oncology	Y	D'Abreo, Garrison, Kwa, Marks, Novik, O'Keeffe, Raince, Ryan, Tegla, Schiff, Wu**, Board E only Goddard**
De los Reyes, Francis	PharmD	S	Pharmacy	Y	Ayoubai, Correll, D'Onofrio, Dubrovskaya, Vasile
Decker, David	BA	NS	IRB Administration	Y	Mann, Maril, McGowan, Mosby, Nicolas, Panageas, Pogorelec-Khan, Vieira
Diefenbach, Catherine	MD	S	Oncology	Y	D'Abreo, Garrison, Kwa, Marks, Novik, O'Keeffe, Raince, Ryan, Tegla, Schiff, Wu**, Board E only Goddard**
Donnino, Robert	MD	S	Medicine – Cardiology	Y	Hazen, Katz, More, Nishawala, Novik, Trinh **Board E only Goddard**
D'Onofrio, Nicole	PharmD, RPh	S	Pharmacy	Y	Ayoubai, Correll, De los Reyes, Dubrovskaya, Vasile
Dubrovskaya, Yanina	PharmD	S	Pharmacy	Y	Ayoubai, Correll, De los Reyes, D'Onofrio, Vasile
Fieremans, Els	PhD	S	Radiology	Y	Ge, Kirov, Lazar, Rusinek, Storey
Frankle, William Gordon	MD	S	Psychiatry	Y	Gallagher, Nishawala, Ross, Schultebraucks**, Board E only Goddard**
Gallagher, Richard	PhD	S	Child & Adolescent Psychiatry	Y	Chervinsky, Frankle, Nishawala, Ross, Schultebraucks
Garrison, Michael	MD	S	Oncology/Hematology	Y	D'Abreo, Davies, Diefenbach, Kwa, Novik, Marks, O'Keeffe, Raince, Ryan, Tegla, Schiff, Silverman, Wu
Ge, Yulin	MD	S	Radiology	Y	Fieremans, Kirov, Lazar, Rusinek, Storey

Goddard, David	MD	S	Rheumatology	Y	Donnino, Hazen, Katz, Marciano, More, Nishawala, Novik, Quinn, Trinh
Grossman, Scott	MD	S	Neurology	Y	Leeman-Markowski, Ross-Rizzo
Hazen, Alexes	MD	S	Plastic Surgery	Y	Katz, More, Nishawala, Novik, Trinh**, Board E only Goddard**
Jeschke-Lopez, Ikoa	MD	S	IRB Administration	Y	Godina
Johnson, Nadia	MS, CIP	S	IRB Administration	Y	Berkovitz, Dutka, Godina, Jeschke-Lopez, Joseph
Joseph, Yonnette	RN, CIP	S	IRB Administration	Y	Berkovitz, Dutka, Godina, Jeschke-Lopez, Johnson
Katz, Stuart	MD	S	Cardiology	Y	Donnino, Hazen, More, Nishawala, Novik, Trinh**, Board E only Goddard**
Kerwin, Todd	MD	S	Cardiology	Y	Donnino
Kim, Sunmi	MD	S	Anesthesiology	Y	Wang**, Board E only Goddard**
Kirov, Ivan	PhD	S	Radiology	Y	Fieremans, Ge, Lazar, Rusinek, Storey
Kirsch, Thorsten	PhD	S	Orthopedic Surgery	Y	Aggarwal, Meftah**,** Prescott, Petrone **Board E only Goddard**
Kuokkanen, Satu	MD, PhD	S	OB/GYN	Y	Mehta-Lee, Nippita, Shanahan, Wissner-Greene
Kwa, Maryann	MD	S	Oncology	Y	D'Abreo, Davies, Diefenbach, Garrison, Marks, Novik, O'Keeffe, Raince, Ryan,Tegla, Schiff, Wu**, Board E only Goddard**
Leeman-Markowski, Beth	MD	S	Neurology	Y	Grossman, Ross-Rizzo
Liu, Jasmine	BS, CIP	NS	IRB Administration	Y	Panageas
Mann, Erik	MPH, CIP	NS	IRB Administration	Y	Panageas
Marciano, Tuvia	MD	S	GI/ Pediatrics	Y	<u>Vice Chair Alternates:</u> Donnino, Hazen, Nishawala<br><br>Goddard, Poppers
Maril, Robert	CIP	NS	IRB Administration	Y	Mann, McGowan, Mosby, Nicolas, Panageas, Pogorelec-Khan, Vieira
Marks, Doug	MD	S	Oncology	Y	Davies, Diefenbach, Garrison, Kwa, Novik, O'Keeffe, Raince, Ryan, Tegla, Schiff, Silverman, Wu
McGowan, Richard	MLS	NS	Public Services	Y	Nicolas, Panageas, Vieira
Meftah, Morteza	MD	S	Orthopedic Surgery	Y	Aggarwal, Kirsch, Petrone, Prescott
Mehta-Lee, Shilpi	MD	S	Ob-Gyn	Y	Kuokkanen, Nippita, Shanahan, Wissner-Greene
More, Frederick	DDS	S	Dentistry – Pediatric	Y	Hazen, Katz, Nishawala, Novik, Trinh
Mosby, Jennifer	MS	NS	IRB Administration	Y	Panageas
Nicolas, Barnaby	MSIS, AHIP	NS	Non-Scientist	Y	McGowan, Vieira

31-MAY-2024

31-MAY-2024

Nishawala, Melissa	MD	S	Child & Adolescent Psychiatry	Y	Vice Chair Alternates: Donnino, Hazen, Katz, More<br><br>Member Alternates: Bogenschutz, Chervinsky, Frankle, Gallagher, Ross, Schultebraucks **Board E only Goddard**
Nolan, Anna	MD	S	Pulmonary	Y	**Board E only Goddard**
Novik, Yelena	MD	S	Oncology	Y	D'Abreo, Davies, Diefenbach, Katz, Kwa, Hazen, More, Nishawala, Tegla, Trinh, Wu**,** **Board E only Goddard**
O'Keeffe, Mary	MD	S	Oncology/Hematology	Y	D'Abreo, Davies, Diefenbach, Garrison, Kwa, Marks, Raince, Tegla, Schiff, Wu
Oshva, Lillian	MD	S	Emergency Medicine	Y	Ramirez**, Board E only Goddard**
Petrone, Patrizio	MD	S	Surgery	Y	Aggarwal, Hazen, Kirsch, Meftah, Prescott
Pogorelec-Kahn, Dana	BA, CIP	NS	IRB Administration	Y	Panageas
Poppers, David	MD	S	Gastroenterology	Y	Marciano
Prescott, Jason	MD	S	Endocrine Surgery	Y	Aggarwal, Kirsch, Meftah, Petrone
Quinn, Gwendolyn	PhD	S	OB/GYN	Y	Donnino, Goddard, Hazen, Katz, More, Nishawala, Novik, Trinh
Raince, Jagdeep	MD	S	Oncology	Y	Davies, Diefenbach, Garrison, Kwa, Marks, Novik, O'Keeffe, Ryan, Tegla, Schiff, Silverman, Wu
Rajan, Anand	MPH	S	Biostatistician	Y	Islam
Ramirez, Michelle	MD	S	Pediatric Critical Care	Y	Brar**, Board E only Goddard**
Raskin, Joyce	JD	NS	Community Representative	N	Basu Roy, Wu
Ross, Stephen	MD	S	Addiction Psychiatry	Y	Bogenschutz, Chervinsky, Frankle, Nishawala, Schultebraucks, **Board E only Goddard**
Rizzo, JohnRoss	MD	S	Neurology	Y	Chervinsky, **Board E only Goddard,** Leeman-Markowski
Rusinek, Henry	PhD	S	Radiology	Y	Fieremans, Ge, Kirov, Lazar, Storey
Ryan, Theresa	MD	S	Oncology	Y	D'Abreo, Davies, Diefenbach, Garrison, Kwa, Marks, Raince,Tegla, Schiff, Wu**,** **Board E only Goddard**
Schiff, Peter	MD	S	Radiation – Oncology	Y	D'Abreo, Davies, Diefenbach, Garrison, Kwa, Marks, O'Keeffe, Raince, Ryan, Silverman,Tegla**, Board E only Goddard**
Schultebraucks, Katharina	PhD	S	Psychiatry	Y	Chervinksy, Frankle, Gallagher, Nishawala, Ross
Shanahan, Lisa	DO	S	OB-GYN	Y	Kuokkanen, Mehta-Lee, Nippita, Wissner-Greene
Storey, Elizabeth	PhD	S	Radiation	Y	Fiermans, Ge, Kirov, Lazar, Rusinek,

Tegla, Cosmin	MD	S	Oncology	Y	D'Abreo, Davies, Diefenbach, Garrison, Kwa, Novik, Marks, O'Keeffe, Raince, Ryan, Schiff, Wu, **Board E only Goddard**
Trinh, Chau	DrPH	S	Population Health	Y	Katz, More, Novik
Vasile, Alicia	RPh	S	Pharmacy	Y	Ayoubai, Corell, De los Reyes, D'Onofrio, Dubrovskaya
Vieira, Dorice	MA	NS	Public Services	Y	McGowan, Nicolas
Wang, Jing	MD	S	Anesthesiology	Y	Kim, **Board E only Goddard**
Wissner-Greene, Loren	MD, MA	S	Medicine – Endocrinology – Ob-Gyn	Y	Kuokkanen, Mehta-Lee, Nippita, Shanahan
Wu, Jennifer	MD	S	Oncology	Y	D'Abreo, Davies, Diefenbach, Garrison, Kwa, Marks, O'Keeffe, Raince, Ryan, Silverman, Tegla, **Board E only Goddard**
Wu, Lillian	BA, MA	NS	Community Representative	N	Basu Roy, Bee, Raskin

31-MAY-2024
