# Supplementary material for: An implementation trial to mAnage siCkle CELl disEase through incReased AdopTion of hydroxyurEa in Nigeria (ACCELERATE): Study protocol
Source: PLoS One. 2025 Jan 8;20(1):e0311900. doi: 10.1371/journal.pone.0311900 (PMC11709263; doi:10.1371/journal.pone.0311900)
Supplement: S3 File — (PDF) [file pone.0311900.s004.pdf]

National Health Research Ethics Committee of Nigeria (NHREC)
Promoting Highest Ethical and Scientific Standards
for Health Research in Nigeria

Federal Ministry of Health

NHREC Protocol Number NHREC/01/01/2007- 21/11/2023
NHREC Approval Number NHREC/01/01/2007- 19/12/2023
Date: 19th December, 2023

Re: Managing Sickle Cell Disease through Increased Adoption of Hydroxyurea in Nigeria (ACCELERATE)

Health Research Committee assigned number: NHREC/01/01/2007

Name of Principal Investigator: Prof. Obiageli E Nnodu
Address of Principal Investigator: Director, CoE for Sickle Cell Disease Research and Training
University of Abuja,
Email: oennodu@gmail.com
Tel: +234-813-0610603

Name of Principal Investigator: Dr. Emmanuel Peprah
Address of Principal Investigator: NYU School of Global Public Health
Global Health Program
Department of Social and Behavioral Sciences
708 Broadway New York, NY10003
Email: ep91@nyu.edu
Tel: +1(212) 992-6085

Date of receipt of valid application: 21/11/2023
Date when final determination of research was made: 19-12-2023

Notice of Full Committee Review and Approval

This is to inform you that the research described in the submitted protocol, the consent forms and other participant information materials have been reviewed and given full committee approval by the National Health Research Ethics Committee.

This approval dates from 19/12/2023 to 18/12/2024. If there is delay in starting the research, please inform the HREC so that the dates of approval can be adjusted accordingly. Note that no participant accrual or activity related to this research may be conducted outside of these dates. *All informed consent forms used in this study must carry the HREC assigned number and duration of HREC approval of the study.* **If this is a multi-year research, endeavour to submit your annual report to the HREC early in order to obtain renewal of your approval and avoid disruption of your research.**

The National Code for Health Research Ethics requires you to comply with all institutional guidelines, rules and regulations and with the tenets of the Code including ensuring that all adverse events are reported promptly to the HREC. No changes are permitted in the research without prior approval by the HREC except in circumstances outlined in the Code. The HREC reserves the right to conduct compliance visit to your research site without previous notification.

Signed

Professor Zubairu Iliyasu MBBS (UniMaid), MPH (Glasg.), MD, PhD, FWACP, FMCPH, FFPH(UK)
Chairman, National Health Research Ethics Committee of Nigeria (NHREC)
