# Supplementary material for: An implementation trial to mAnage siCkle CELl disEase through incReased AdopTion of hydroxyurEa in Nigeria (ACCELERATE): Study protocol
Source: PLoS One. 2025 Jan 8;20(1):e0311900. doi: 10.1371/journal.pone.0311900 (PMC11709263; doi:10.1371/journal.pone.0311900)
Supplement: S4 File — (PDF) [file pone.0311900.s005.pdf]

UNIVERSITY OF ABUJA TEACHING HOSPITAL

P.M.B. 228 ABUJA - F.C.T. NIGERIA
☎ 07040045614, 07010993173,
www. uath.gov.ng.
Email: info@uath.gov.ng.

Chief Medical Director
Professor Bissallah A. Ekele
FWACS, FICS, FRCOG

Chairman Medical Advisory Committee
Dr. Bob Agwu Ukonu
MBBS, FMCP

Director of Administration
Modupe K. Adebanjo (Mrs)
BA, M.Sc, FCIA, AHAN

Our Ref ~~FCT/UATH/HREC/1085~~

Date ~~05/2/2024~~

UATH HREC Protocol number: *UATH/HREC/PR/2024/02/135*
UATH HREC Approval Number: *UATH/HREC/PR/2024/02/145*

Proposed Title: **mAnaging sickle disease through increased AdopTion of hydorxyurEa in Nigeria (ACCELARATE)**

Name of Investigators: **Prof. Obiageli Eunice Nnodu**
Dr. Emmanuel Peprah, MD

Address of Investigator: Department of Haematology and Blood Transfusion, UATH Abuja Gwagwalada

Date of Receipt of Valid Application:	29/01/2024
Date of full Committee Approval:	14/02/2024
Proposed Site:	UATH
Sponsor:	Principal Investigator

This is to inform you that the activity described in the submitted protocol/documents have been reviewed and the UATH HREC has determined that according to the National Code for Health Research, the activity described has met the criteria for approval and thus approved.

The National Code for Health Research Ethics requires you to comply with all institutional guidelines, rules, regulations and the tenets of the code.

The approval is for one year and will lapse on 14/02/2025. However, it could be renewed on request by application Four weeks before the expiration of this approval, if the life span of the project exceeds February, 2025.

Accept assurances of our highest regards, please.

15/2/24

Prof. Aliyu Y. Isah
Chairman UATH HREC
