# Supplementary material for: An implementation trial to mAnage siCkle CELl disEase through incReased AdopTion of hydroxyurEa in Nigeria (ACCELERATE): Study protocol
Source: PLoS One. 2025 Jan 8;20(1):e0311900. doi: 10.1371/journal.pone.0311900 (PMC11709263; doi:10.1371/journal.pone.0311900)
Supplement: S5 File — (PDF) [file pone.0311900.s006.pdf]

# mAnaging siCkle CELl disEase through incReased AdopTion of hydroxyurEa in Nigeria (ACCELERATE)

**Principal Investigator**
Dr. Emmanuel Peprah, PhD
NYU Langone Health

**Co-Principal Investigator**
Dr. Obiageli Nnodu, MD
University of Abuja

**Co-Investigator**
Dr. Gbenga Ogedegbe, MD
NYU Langone Health

**Protocol Version**
29 April 2024

## ABBREVIATIONS

HU - Hydroxyurea
CESTRA - The Centre of Excellence for Sickle Cell Disease Research and Training
SCD - Sickle Cell Disease
REACH - Realizing Effectiveness Across Continents with Hydroxyurea
TASSH - TAsk-Strengthening Strategy for Hemoglobinopathies
TCP - Training healthcare workers and providers to be more patient-centered in clinical consultations + ii) Clinical reminders + iii) Practice facilitation
EPIS - Exploration, Preparation, Implementation, and Sustainment
SSA - Sub-Saharan Africa
ACS - acute chest syndrome
EBP - evidence-based practice
SPARCO - Sickle Pan African Research Consortium
SCSSN - Sickle Cell Support Society of Nigeria
CESRTA - Centre of Excellence for Sickle Cell Disease Research and Training, University of Abuja
SPARC-NEt - Sickle Pan African Research Consortium - Nigeria
SIM - Screening, Initiation, and Maintenance
SADaCC - Sickle Africa Data Coordinating Centre
LMIC - low- and middle-income country
MDD - minimum detectable difference
ICC - intra-class coefficients
CONSA - Consortium for Newborn Screening in Africa
SDG - Sustainable Development Goal
LOS - letters of support
EMR - electronic medical records
ARC - absolute reticulocyte count
ANC - absolute neutrophil count
CBC - Complete Blood Count
CML - chronic myeloid leukemia
OSMB - Observational Safety and Monitoring Board
REDCap - Research Electronic Data Capture
FIML - full information maximum likelihood

## SYNOPSIS

**Primary Objective**
To evaluate the level of hydroxyurea (HU) adoption among healthcare providers (i.e., physicians and nurses) of our task-sharing (SIM) intervention in Nigeria and determine if HU adoption improved patient-level SCD management and outcomes.

**Secondary Objectives**
To assess the sustainability and implementation fidelity of our task-sharing intervention after completion of the 12-month implementation trial.

- To evaluate organizational factors as potential predictors of HU adoption using the task-sharing strategy.

**General Design Description**
Utilizing a robust mixed-methods study design, our investigation unfolds across four distinct phases of the Exploration, Preparation, Implementation and Sustainment (EPIS) framework, meticulously examining the multifaceted landscape of HU utilization at the provider level. Simultaneously, we assess the capacity of SPARC-NEt Nigeria clinical sites to embrace the SIM approach. Our inquiry extends to scrutinizing the inner organizational context, meticulously examining factors such as the existing SCD management protocols, staffing profiles (comprising nurses, hematologists, and non-hematologist physicians), and prevailing prescription practices among providers.

In the initial Exploration Phase of EPIS, our objective is to delve into the facilitators and barriers influencing the prescription of HU among providers. Through qualitative stakeholder interviews

conducted at the provider level, we aim to unravel the nuanced attitudes and perceptions that shape providers' decisions regarding HU prescription for SCD patients. The outcomes of these interviews will illuminate potential barriers hindering HU prescription. For instance, if concerns regarding medication costs emerge prominently during these qualitative sessions, we will actively address them by disseminating information that underscores the coverage of HU under the National Health Insurance Authority Act of 2022 (https://archive.gazettes.africa/archive/ng/2022/ng-government-gazette-dated-2022-05-24-no-95.pdf). We will explore the inner organizational context to adapt a tailored intervention, including examining the SCD management standard protocol at clinical sites, staffing data (i.e., numbers of nurses, hematologist, and non-hematologist physicians available), and provider prescription practices for HU. This phase of exploration promises to enrich our understanding of the specific contextual factors surrounding the initiation of provider training for HU in Nigeria. Providers and patients from diverse clinical sites will be recruited to participate, ensuring a comprehensive representation of perspectives. Upon the completion of our qualitative exploration, we will transition to the Preparation Phase of EPIS. Drawing upon insights garnered from the Exploration Phase, we will curate evidence-based materials on HU, leveraging resources such as the American Society of Hematology (ASH) pocket guides for SCD management (https://www.hematology.org/education/clinicians/guidelines-and-quality-care/pocket-guides). In the Preparation Phase, we adapt the provider-level task-sharing implementation strategy. Our adaptive process ensures the tailored TASSH TCP strategy aligns seamlessly with the context-specific needs and challenges identified. Through this iterative refinement, we can fine-tune our approach to maximize impact and relevance within the unique landscape of Nigeria clinical sites. During this phase, we will meticulously chart out the training cycles for non-hematologist physicians and nurses across the 20 clinical sites. We will also train Practice Outreach Facilitators (POFs) whom will provide clinical support for the implementation of the intervention during this phase. These sites encompass a spectrum of healthcare facilities, ranging from tertiary-level institutions like the University of Abuja Teaching Hospital to primary care facilities including Primary Healthcare Centers (PHC). We will conscientiously identify convenient timings for providers and POFs to undergo training, ensuring optimal participation, and subsequently develop a structured follow-up training schedule to sustain engagement and knowledge retention among participating providers.

During the Implementation Phase of EPIS, we will deploy our comprehensive SCD management and HU training program to providers across 20 clinical sites participating in a cluster RCT. Within this phase, providers will be randomly assigned to one of two arms within the intervention trial. The first arm will comprise physicians and nurses who will undergo specialized training in the administration of HU and SCD management. Additionally, these physicians and nurses will receive ASH pocket guides and will be supported by highly skilled nurses with extensive experience in both HU administration and SCD management at their local clinical sites. Furthermore, this intervention arm will benefit from periodic refresher courses on prescribing HU for SCD patients and providers will be supported by POFs. This arm will encompass 10 sites, including Primary Healthcare Centers (PHC) and Teaching Hospitals. The second arm, designated as the Control Arm, will consist of physicians and nurses randomly allocated across 10 sites. In this arm, healthcare providers will receive initial training on HU administration and relevant information such as ASH pocket guides. However, unlike the Intervention Arm, they will not receive ongoing support in the form of trained nurses. Throughout the study, investigators will evaluate the prescription behavior of physicians in both arms at 0 and 12 months across all 20 clinical sites. This analysis aims to discern whether the prescription of HU has increased within either or both arms of the cluster RCT.

Moving to the Sustainment Phase, following 12 months of providing support to the intervention arm, we will withdraw clinical support (i.e., remove POF from clinics/health facilities in the intervention arm) and highly trained nurses from this group. Subsequently, we will assess the prescription of HU at the 24-month mark. Sustainment or maintenance will be determined by comparing the prescription rates of HU between the periods of 12-24 months and the initial baseline of 0-12 months across all 20 clinical sites. We will also assess the implementation fidelity of the SIM intervention across all 20 clinical sites to determine whether differences in implementation of the intervention occurred based on site specific factors.

Based on Proctor et al (2011) definition, in essence sustainability is “extent to which a newly implemented treatment is maintained or institutionalized within a service setting’s ongoing, stable operations”…hence our objective is to assess how our strategy of SIM has been embedded into the existing system. This is true sustainability, because in essence external support via grant funds would not be needed and hence we should observe positive downstream affects on providers…and finally patients.

**Duration of the project**
The project spans a 5-year duration in Abuja, Nigeria, with NYU exclusively receiving de-identified data from clinical sites and the University of Abuja. Pre-implementation endeavors are slated for years 1-2, encompassing exploration activities to grasp the implementation landscape, training study providers, and enrolling eligible SCD participants after securing NYU's IRB approval. Year 3 marks the commencement of intervention implementation, coinciding with a two-year cluster RCT. Year 4 focuses on sustaining the intervention at clinical sites, while year 5 sees ongoing data analysis across all sites. Implementation efforts persist for two years, entirely within Nigeria, with NYU serving as the data coordinating hub, ensuring meticulous oversight of all data management tasks while only accessing de-identified data. NYU personnel will visit Nigeria twice yearly; post-IRB approval and before study commencement, maintaining rigorous engagement throughout the study period.

**Primary Outcome Variables**
The primary outcome is the adoption of the SIM intervention, measured by an increase in HU prescription rates for eligible SCD patients facilitated by providers. We hypothesize that clinical sites randomized to the experimental arm of the cluster RCT will exhibit higher levels of SIM adoption compared to those in the control arm. Additionally, our secondary aims include evaluating the sustainability/maintenance of the intervention across clinical sites at 24 months. We anticipate that factors identified in the Exploratory phase of EPIS, related to the inner organizational context, outer context, and implementation process will influence both the adoption and sustainability of the SIM intervention at clinical sites.

**Secondary and Exploratory Outcome Variables**
Secondary outcome measures are implementation fidelity and sustainability across the clinical sites at 12 and 24 months. The following measures will be used to assess the mediators of SIM via self-report. The mediators are based on the constructs of the EPIS including inner context characteristics of the clinics, intervention characteristics, and implementation process measures. In brief, our hypothesis is that the sustainability and implementation fidelity of SIM will be higher in the sites randomized to TASSH TCP than those in control condition (no TASSH TCP). Sustainability is another key implementation outcome and a priority topic in implementation science. Our approach is to use the EPIS framework to evaluate the maintenance of SIM at participating health facilities over time.

**Number of Participants**
Eligible SCD patients (adults and children / both male and females) will be identified from the CONSA NBS program and the SPARC-NEt Nigeria database. Once study is approved, potential study participants will be contacted by a nurse from each of the clinical sites during routine primary care visits with families. Each of the 20 sites will recruit 45 eligible patients for a total sample size of 900 who meet the following eligibility criteria over a 2-year period. Study activities will be conducted at each of the respective sites only after all appropriate institutional / ethics committee approvals are in place.

SCD Providers: Hematologist, Non-Hematologist physician, Nurse, Community health workers (2 per site) will participate in the training and delivery of the intervention. 40 providers will participate in the study across all sites.

**Table of Contents**

***SYNOPSIS........................................................................................................................ 2***
***1. Statement of Compliance........................................................................................................1***
***2. Background..........................................................................................................................6***
***3. Rationale/Significance........................................................................................................ 7***
**3.1 Problem Statement..................................................................................................7**
**3.2 Purpose of Study/Potential Impact.............................................................................. 8**
**3.3 Potential Risks**

**and Benefits............................................................................................................... 8**
3.3.1 Potential Risks.......................................................................................................... 8
3.3.2 Potential Benefits.......................................................................................................9
***4. Study Objectives...................................................................................................................9***
**4.1 Hypothesis.................................................................................................................. 9**
**4.2 Primary Objective.......................................................................................................10**
**4.3 Secondary Objectives ................................................................................................10**
***5. Study Design..................................................................................................................... 11***
**5.1 General Design Description.......................................................................................11**
**5.2 Outcome Variables.................................................................................................... 11**
5.2.1 Primary Outcome Variables..................................................................................... 12
5.2.2 Secondary and Exploratory Outcome Variables ...................................................... 12
***6. Study Population................................................................................................................ 13***
**6.1 Study Population.......................................................................................................13**
6.1.1 Number of Participants............................................................................................ 15
6.1.2 Eligibility Criteria/Vulnerable Populations................................................................ 15
***7. Methods..............................................................................................................................16***
**7.1 Intervention................................................................................................................16**
7.1.1 Description of Intervention........................................................................................ 16
7.1.2 Method of Assignment/Randomization......................................................................17
7.1.3 Selection of Instruments/Outcome Measures............................................................18
7.1.4 Intervention Administration....................................................................................... 18
7.1.5 Reaction Management..............................................................................................21
**7.2 Assessments............................................................................................................. 22**
7.2.1 Efficacy.................................................................................................................... 22
7.2.2 Safety/Pregnancy-related Procedure........................................................................22
7.2.3 Adverse Events Definition and Reporting..................................................................22
7.2.4 Pharmacokinetics .................................................................................................... 22
7.2.5 Biomarkers ...............................................................................................................22
**7.3 Study Procedures.......................................................................................................25**
7.3.1 Study Schedule.........................................................................................................25
7.3.2 Informed Consent..................................................................................................... 25
7.3.3 Screening..................................................................................................................26
7.3.4 Recruitment, Enrollment and Retention.....................................................................26
7.3.5 Study Visits.............................................................................................................. 27
7.3.6 End of Study and Follow Up..................................................................................... 27
7.3.7 Removal of Subjects..................................................................................................27
**7.4 Statistical Method.......................................................................................................28**
7.4.1 Statistical Design...................................................................................................... 28
7.4.2 Sample Size Considerations......................................................................................28
7.4.3 Planned Analyses..................................................................................................... 28
7.4.3.1 Primary Analyses......................................................................................................28
7.4.3.2 Secondary Objectives Analyses, ............................................................................. 29
7.4.3.3 Analysis of Subject Characteristics............................................................................29
7.4.3.4 Interim Analysis ....................................................................................................... 30
7.4.3.5 Health economic evaluation, ....................................................................................**30**
7.4.3.6 Other........................................................................................................................ 31

7.4.4 Subsets and Covariates....31
7.4.5 Handling of Missing Data....31
***8. Trial Administration....31***
8.1 Ethical Considerations: Informed Consent/Assent and HIPAA Authorization....31
8.2 Institutional Review Board (IRB) Review....31
8.3 Subject Privacy, Confidentiality & Data Management....32
8.4 Deviations/Unanticipated Problems....32
8.5 Data Collection.... 33
8.6 Data Quality Assurance....33
8.7 Study Records.... 33
8.8 Access to Source....33
8.9 Data Storage/Security.... 33
8.10 Retention of Records....33
8.11 Study Monitoring....33
8.12 Data Safety Monitoring Plan....33
8.13 Study Modification.... 34
8.14 Study Discontinuation....35
8.15 Study Completion.... 35
8.16 Conflict of Interest Management Plan.... 35
8.17 Funding Source.... 35
8.18 Publication Plan....35
***REFERENCES.... 35***
***APPENDICS....35***

## 1. Statement of Compliance

This study will be conducted in accordance with the Code of Federal Regulations on the Protection of Human Subjects (45 CFR Part 46), any other applicable US government research regulations, and institutional research policies and procedures. The Principal Investigator will assure that no deviation from, or changes to the protocol will take place without prior agreement from the sponsor and documented approval from the Institutional Review Board (IRB), except where necessary to eliminate an immediate hazard(s) to the study participants. All personnel involved in the conduct of this study have completed Human Subjects Protection Training.

## 2. Background

Sub-Saharan Africa (SSA) bears the greatest SCD burden in the world, with over 300,000 people affected.[1,2] SCD, the inherited blood disorder characterized by anemia, severe pain, and other vaso-occlusive complications, acute chest syndrome (ACS), disproportionate hospitalization, and early mortality, has significant financial, social, and psychosocial impacts and drains individuals, families, and health systems. It is projected that over 300,000 individuals in SSA are born annually with SCD.[3-5] SCD is a progressively debilitating and chronic multi-organ disease with a 30-50% incidence of disability and unemployment, as well as the leading cause of stroke in children and adolescents.[6] Comprehensive clinical care programs in the US have reduced childhood mortality attributed to SCD by 70%.[7] Unfortunately, translation of this evidence-based intervention/practice (EBI/P) is lacking in Africa; where 50—90% of children with SCD die before the age of 10.[8]

Nigeria has the highest prevalence of SCD (~4 million of total population of 202 million), with most undiagnosed and not linked to care.[9] About 150,000 babies are born annually with SCD in Nigeria;[10] this burden can be substantially reduced by early diagnosis and supportive care with penicillin prophylaxis and HU treatment.[11,12] However, these interventions have not been widely implemented in SCD patients in Nigeria. There is also low adoption in prescribing HU and anti-pneumococcal prophylaxis amongst Nigerian caregivers.[13.]

Widespread adoption of HU in Nigeria is sub-optimal. HU therapy, a cost-effective myelosuppressive agent, is very well tolerated with minimum short-term toxicity, and it significantly reduces the number of SCD-related painful episodes, ACS, transfusions, and hospitalizations.[14-16] However, HU administration, including laboratory monitoring, requires specialized training to improve prescription and monitoring practice. Galadanci et al. (2014) found that only 8 out of 18 SCD specialist health institutions studied in Nigeria prescribed HU to their patients,[17] and within those institutions, only 5-33% of their patients-maintained HU use.[18-21]

Provider-level barriers impede the adoption of HU therapy for SCD management in Nigeria.[22-25] The vast majority of people with SCD in Africa do not receive evidenced-based health care (e.g., newborn screening, health education, prophylaxis for infection, optimal nutrition and hydration, blood transfusion, transcranial Doppler screening, and HU therapy), despite its effectiveness in reducing SCD-related adverse outcomes and mortality. The use of HU in SSA is <1% among SCD patients.[26] As highlighted in our preliminary data and existing research, cost and laboratory monitoring is a significant barrier to the use of HU as an evidence-based intervention (EBI) to address SCD in Africa[.27,28] Provider-level factors like HU acceptability, fidelity, delivery context/platform, and adoption have not been rigorously assessed. Our preliminary findings indicate that provider-level barriers are significant and must be addressed to improve HU adoption.[20,21,29,30] Our recent survey of 87 providers (e.g., nurses and doctors in Nigeria) who are part of the NHLBI-funded Sickle Pan African Research Consortium (SPARCO) network (i.e., Nigeria, Ghana, and Tanzania) suggests that barriers to HU prescription include inadequate formal healthcare worker training, lack of clinical reminders, and the need for performance feedback to improve SCD management in Nigeria[31] exacerbated by the shortage of hematologists.

Task sharing is an evidence-based implementation strategy for effective management of SCD. One of the greatest challenges to optimal use of HU and management of SCD in Nigeria, as throughout SSA, is the acute shortage of hematologists. As of 2013, there were 4.1 physicians and 16.1 nursing/midwifery personnel per 10,000 patients in Nigeria.[32] The shortage of health workers is partly due to the brain drain of health workers who migrate to high-income countries for better standards of living, higher salaries, and stable political conditions.[33] There is an urgent need to increase capacity for SCD management among non-hematologists to provide care for SCD patients. One such approach is a task-sharing strategy, defined as the rational distribution of tasks among health workforce teams,[34] including the redistribution of SCD care duties among hematologists, non-hematologist physicians, and nurses. Task sharing/shifting is useful in low-resource settings that face acute healthcare human resource crises.[35] Our previous research utilizing task sharing (i.e., TASSH) for other chronic diseases (e.g., HIV

and hypertension) in West Africa suggests it is an effective strategy to improve adoption and delivery of EBI in resource constrained settings.[34,36-42]

EBIs targeted at SCD management must address provider-level barriers of care delivery. The Sickle Cell Support Society of Nigeria (SCSSN) was formed by physicians, nurses, individuals with SCD, and other stakeholders, with Prof. Nnodu as a founding member. The Centre of Excellence for Sickle Cell Disease Research and Training, University of Abuja (CESRTA), has a multidisciplinary team of basic and clinical scientists, educationists, and social scientists working in collaboration to execute translational research in SCD. Prof. Nnodu has worked with members of the SCSSN on technical committees of the Federal Ministry of Health to develop SCD management guidelines and newborn screening policies and coordinated translational research activities. Sickle Pan African Research Consortium - Nigeria (SPARC-NEt) is located at CESRTA and collaborated with the SCSSN to form a 20-site consortium.[9]

Increasing HU adoption by providers requires leveraging consortium resources to conduct needed implementation research to bridge the evidence to practice gap for SCD management. Leveraging SPARC-NEt's clinical research infrastructure to conduct implementation research on service delivery and building capacity at the provider level will improve HU adoption and SCD management in Nigeria.[25,43-46]

Provider-level training of non-hematologist physicians and nurses in the appropriate use and management of HU can improve prescription practices [1,43] and ensure that SCD management can be maintained with fidelity at SPARC-NEt clinical sites via task sharing. Because the number of hematologists available in Nigeria is limited,[47,48] we believe that the task-sharing strategy can also improve HU adoption at clinical sites. There is an urgent need and an opportunity to build local capacity to implement strategies to facilitate adoption of HU to improve SCD outcomes.

There are not enough hematologists in Nigeria to manage patients with SCD. Our study will evaluate the adoption and maintenance of the algorithm developed by the evidenced-based clinical trial study, REACH, which we call the Screening, Initiation, and Maintenance (SIM) strategy combined with our implementation strategy for task sharing of SCD management that has three components; i) Training healthcare workers and providers to be more patient-centered in clinical consultations + ii) Clinical reminders + iii) Practice facilitation (TASSH TCP).

Our strategy of combining healthcare worker training + clinical reminders + practice facilitation maps to our previous research utilizing task sharing (i.e., TASSH) for other chronic diseases in West Africa which suggests TASSH is an effective implementation strategy to improve adoption and delivery of EBI in resource constrained settings [34,36-42] and will foster sustainability of the SIM intervention effects after study completion.

This project gives us a unique opportunity to fill this research-to-practice gap by evaluating the effectiveness of a practical and replicable strategy for implementing SIM+TASSH TCP using clinical evidence from the NHLBI-funded REACH Clinical Trial, [26] to address the adoption of our intervention for SCD management and evaluate its effects via a sequential exploratory mixed-methods[49] study design on the adoption, implementation fidelity, and sustainability of the implementation strategy across SPARC-NEt clinical sites with over 9,000 SCD patients. The strategy leverages existing NHLBI-funded research infrastructure of the SPARC-NEt sites [50] and existing Sickle Africa Data Coordinating Centre (SADaCC).[51]

A 2017 Cochrane review shows our TASSH implementation strategy is sustainable and has been demonstrated to be effective in high income countries[52,53] and low- and middle-income countries (LMICs),[54] although our TASSH TCP is yet to be widely applied in LMICs,[54] especially for SCD management. Moreover, healthcare providers in LMICs lack the resources and expertise to implement systematic change without assistance. Our evidence-based implementation strategy may effectively overcome the barriers identified in our preliminary data. This PF[53,55-57] strategy provides external practice redesign expertise and a tailored approach to implement guideline-concordant care.[52]

## 3. Rationale/Significance

### 3.1 Problem Statement

Large knowledge gaps remain regarding strategies to promote the adoption of HU, particularly in sub-Saharan African countries including Nigeria, where more than 75% of annual sickle cell anemia births occur. The vast

majority of people with SCD in Africa do not receive evidenced-based health care (e.g., newborn screening, health education, prophylaxis for infection, optimal nutrition and hydration, blood transfusion, transcranial Doppler screening, and HU therapy), despite its effectiveness in reducing SCD-related adverse outcomes and mortality. The use of HU in SSA is <1% among SCD patients. Our preliminary findings indicate that provider-level barriers are significant and must be addressed to improve HU adoption at the provider-level. To address HU adoption, we will use the NIH-funded study, REACH (NCT01966731)) that developed an evidence-informed, clinical, practical, and easy-to-follow algorithm to 1) Screen patients for SCD, 2) Initiate HU treatment, and 3) Maintain HU dosage over time (SIM) for the improved management of SCD as our intervention aimed at providers in Nigeria. The Nigerian government released guidelines supporting the SIM intervention for HU adoption for improved SCD management, and HU is on the list of essential medicines for Nigeria. Our implementation strategy for improving SCD management in Nigeria uses a practical and replicable evidence-based task-sharing strategy, TAsk-Strengthening Strategy for Hemoglobinopathies (TASSH), adopted from our TAsk-Strengthening Strategy for Hypertension control (TASSH) trials in Ghana and Nigeria containing the essential implementation strategies that include: i) **T**raining healthcare workers/providers to be more patient-centered in clinical consultations, ii) **C**linical reminders, and iii) **P**ractice facilitation (**TCP**) known as (TASSH TCP) for SCD management. Using a sequential exploratory mixed-methods study design, we will conduct this study using the EPIS framework in four sequential phases to assess the effectiveness of SIM adoption by providers in the context of the TASSH TCP implementation strategy in Nigeria.

**Rationale.** We are not aware of any clinical trial that explored factors influencing the implementation and adoption of clinical evidence from the NHLBI-funded REACH Clinical Trial study across SPARC-NEt clinical sites in Nigeria in "real world" settings using EPIS or a similar framework.[16,17] The implementation framework, EPIS will be used as a guide to explore inner context variables (i.e. organization, quality and fidelity, monitoring/support, and leadership support) and provider-level variables and patient characteristics (outer context) likely to influence the adoption and sustainability of SIM.

**Significance.** Our study leverages the infrastructure of the SPARC-NEt (U01HL156942) of Nigeria to assess the adoption of HU among providers to improve SCD management in a manner that is scalable and sustainable across Nigeria and identify best practices for implementing HU therapy in resource constrained settings. We have leveraged the NHLBI-funded SPARC-NEt sites to; i) obtained ethical approvals at national and local institutional levels, ii) established consortium-wide SCD-specific data elements, iii) access the world's largest SCD patient-consented electronic database of over 9,103 pediatric and adult patients with SCD[26] for a first of its kind implementation trial. Our plan to evaluate sustainability of intervention effects beyond the study period is quite innovative, given the lack of data on sustainability for HU and SCD management interventions in LMICs. Nurses and non-hematologist physicians at SPARC-NEt clinical sites will deliver the intervention via task-sharing strategy (i.e., TASSH TCP), making program generalizability and SIM sustainability more likely. The health workers being engaged will align with the local policy for SCD management. Moreover, we will develop a tool kit for wider dissemination of study findings. In addition, our study will be conducted in partnership with Sickle Cell Support Society of Nigeria (SCSSN) and Nigerian Medical Association (NMA) in alignment with the local policy of using trained local nurses as Practice Outreach Facilitators to support providers as they deliver health care services to SCD patients, ensuring SIM intervention effect is sustained.

### 3.2 Purpose of Study/Potential Impact

To address the significant problem of SCD patients who are not linked to care in Nigeria, we will implement the SIM intervention at the provider level to increase HU adoption and improve health outcomes for SCD patients in 20 sites in Nigeria. Our strategy is to use a context specific TASSH-TCP strategy to embed SIM into the workflow of the clinical site to promote provider-level adoption and sustainability, guided by the EPIS framework, which provides a structured model for evaluating change processes and long-term impacts. We will also assess the mediators of implementation of the evidence-based strategy, adoption, implementation fidelity and sustainability of this intervention in Nigeria. The intervention will be implemented by systematically leveraging the NHLBI-funded SPARC-NEt platform. We anticipate that the findings will inform best practices to address HU adoption for SCD management in Nigeria and other countries in the Sickle in Africa consortium (https://www.sickleinafrica.org/).

### 3.3 Potential Risks and Benefits

#### 3.3.1 Potential Risks

All patients will receive HU monitoring as part of their standard medical care.

As part of usual care, all administration of HU will be conducted in accordance with FDA-approved indications, and Nigeria (local) guidelines for the administration of HU and patient-level treatment decisions will be made by the provider due to their medical judgement, not as part of this protocol.

Hydroxyurea (HU) therapy is already administrated in Nigeria as standard of care but not to all eligible SCD patients due to the lack of provider training. HU is very well tolerated with minimum short-term toxicity including carcinogenesis, reversible oligospermia, reactivation of latent tuberculosis, and patient compliance. Side effects including skin rashes, dark patches on skin, vomiting, and dizziness have been observed in some patients who use HU in Nigeria. We have an extensive case report forms (CRFs) to document patient side effects although this is a provider-level intervention. Our rationale has always been to ensure the safety of patients that aligns with established protocols for care of SCD patients on HU during this pragmatic implementation trial. For example, HU will be administered by trained Nigerian physicians, this pragmatic trial is overseen by a world renowned Nigerian hematologist who has managed SCD patients in Nigeria for over 20+ years. Furthermore, we also have OSMB and that will review all patient reported side-affects. Also, to ensure confidentiality, we will obtain written consent from subjects who participate in surveys and interviews; however, no identifying information will be included in the transcripts of interviews or surveys. Furthermore, we will follow established guidelines for HU administration from the FDA and American Society of Hematology which has produced specific pocket guides for providing HU for SCD patients. All survey data will be stored on a password-protected computer. SPARC-NEt has considerable infrastructure that we will leverage to execute this implementation trial. Leveraging the SPARC-NEt platform will allow us to utilize the NIH-funded infrastructure built for clinical research to address the significant knowledge/practice gap for HU adoption for SCD management and leverage the expertise within the Sickle in Africa, a consortium composed of six countries undertaking SCD research in both implementation and clinical research.

SPARC-NEt has established common data elements and secure databases to ensure that all survey data entered into the research database will be protected by confidential entry codes. Names will be replaced with identification numbers. Patients will have the right to refuse to participate without any compromise of their care. Also, if a participant is uncomfortable during a research encounter, they may stop at any time.

SPARC-NEt has also developed protocols to ensure that all patient data will be de-identified prior to transfer to NYU. Additional levels of security exist, including locked file cabinets for storing materials with identifying information (e.g., patient consent forms) at University of Abuja. All computer systems are protected from possible external access. No Internet access is possible with the research systems. The data collected for this study will be used strictly for the purposes stated in this grant application and will only be available to relevant research staff. IRB approval will be sought prior to any data collection involving human subjects.

Collection of blood samples from adults (10mL) and children (3ml). for laboratory use may cause some discomfort to the patients. However, experienced lab technicians will perform this procedure and will be trained to minimize risk. Taking blood may cause dizziness, fainting, pain, bruising, bleeding from where the needle entered your skin, pain, and / or infection. Relevant medical expertise and trained personnel are available to handle such adverse effects if they occur including adequate patient hydration, pain management, bleeding control and infection management.

As part of routine HU monitoring, blood samples will be collected via venipuncture, utilizing either the antecubital or dorsal vein, aligning with the standard protocol recommended for patients as young as 12 months in Nigeria. While venipuncture is the preferred method, acknowledging that for certain children, especially those for whom a finger-prick might be more suitable, this method could be considered optimal. Therefore, we will defer to the discretion of the physician or nurse to determine the most appropriate method, considering caregiver/guardian preferences or cultural considerations, while prioritizing sensitivity to the child's comfort is paramount throughout the process. Adherence to the World Health Organization Guidelines on Drawing Blood: Best Practices in Phlebotomy ensures standardize procedures for blood draws in pediatric patients, with consideration given to factors such as age, weight, and potential hindrances like calluses on the feet for finger-prick selection. The process of performing venipuncture on children aged 12 months and older demands not only technical proficiency but also a compassionate, patient-centered approach. Following the guidelines endorsed by the Paediatric

Association of Nigeria (PAN), local protocols will be followed with blood draws done by trained physicians or nurses. The volume of blood collected will be minimized to reduce the risk of adverse effects, typically ranging from 0.5 milliliters (mL) to 3 mL for routine laboratory tests like complete blood count (CBC), based on individual clinical assessment and test requirements. Thorough preparation, including clear communication and positioning, will help with vein selection, while the use of antiseptic swabs and optional topical anesthesia reduces discomfort. Skillful needle insertion, coupled with strict monitoring of blood flow and gentle pressure, stabilizes the vein during the procedure. Post-procedure care, as indicated by PAN guidelines, emphasizes measures to promote hemostasis, proper disposal of sharps, and comprehensive instructions for follow-up care, with rigorous documentation ensuring accuracy and accountability throughout. The purpose of the blood collection is to monitor neutropenia when on HU. This is standard of care for any individual beginning HU treatment for SCD and ensures that patients that are not tolerant of HU are identified and treatment is discontinued. Based on the SCD literature, these patients are a small population, and clinical monitoring of the cohort ensures patient safety at the initiation of HU.

***For both patients receiving HU monitoring and patients and providers participating in focus groups:***
It is also possible some subjects may experience a mild stress during quantitative and qualitative survey completion response, anxiety, or some other form of emotional distress during study activities. However, if some of the questions cause uncomfortable feelings, counseling will be offered. Also, subjects do not have to answer any questions they choose not to. Subjects might feel inconvenienced by giving their time for the study visits and meetings with their provider and study team.

There is a small risk people not connected with this study will learn subjects' identity or personal information. We will follow all institutional standards to keep this information safe.

Providers in the intervention arm will receive enhanced SIM training with clinical support while those in the control arm will follow SIM, with no additional clinical support. All patients will receive information about SCD management adopted from the American Society of Hematology.

### 3.3.2 Potential Benefits

***ALL Patients will receive HU monitoring.***
Subjects will not benefit directly from their participation in this study (i.e. from completing the blood draws, surveys, focus groups, etc.).

***For both Patients receiving HU monitoring and Patients and providers participating in Focus groups:***

You will not directly benefit from completing the surveys and taking part in the focus group. However, the results of this research study will contribute directly to the understanding of ways in which programs like this may be used to improve SCD management and the quality of life of SCD patients in Nigeria and other similar settings.

This study will yield knowledge regarding methods for adoption of HU for significant reduction of adverse effects (e.g., the number of painful episodes, acute chest syndrome, transfusions, and hospitalizations). Overall, the benefits of understanding effective methods for helping patients reduce their risk of adverse effects of SCD is important and may improve quality of health.

The study may have relevance to the Nigerian health care system and other low resource settings by testing new strategies to enhance implementation, adoption, and sustainment of evidence-based interventions including building capacity for well-trained SCD providers to improve SCD management in high-risk populations.

## 4. Study Objectives

### 4.1 Hypothesis

For the primary aim, we hypothesize that clinical sites randomized to the experimental arm (SIM+TASSH TCP) of the cluster RCT will exhibit higher levels of SIM adoption compared to those in the control arm (SIM, no TASSH

TCP) for SCD management.

For our secondary aim, which includes evaluating the implementation fidelity and sustainability/maintenance of the intervention across clinical sites at 24 months. We anticipate that factors related to the inner organizational context, outer context, and implementation process will influence both the adoption and sustainability of the SIM intervention at clinical sites.

**4.2 Primary Objective**

To evaluate the level of HU adoption among health care providers (i.e., physicians and nurses) of our task-sharing (SIM) intervention in Nigeria and determine if HU adoption improved patient-level SCD management and outcomes.

**4.3 Secondary Objectives**

To assess the sustainability and implementation fidelity of our task-sharing intervention after completion of the 12-month implementation trial.

- To evaluate organizational factors as potential predictors of HU adoption using the task-sharing strategy.

**5. Study Design**

**5.1 General Design Description**

Utilizing a robust mixed-methods study design, our investigation unfolds across four distinct phases of the Exploration, Preparation, Implementation and Sustainment (EPIS) framework, meticulously examining the multifaceted landscape of HU utilization at the provider level. Simultaneously, we assess the capacity of SPARC-NEt Nigeria clinical sites to embrace the SIM approach. Our inquiry extends to scrutinizing the inner organizational context, meticulously examining factors such as the existing SCD management protocols, staffing profiles (comprising nurses, hematologists, and non-hematologist physicians), and prevailing prescription practices among providers.

In the initial Exploration Phase of EPIS, our objective is to delve into the facilitators and barriers influencing the prescription of HU among providers. Through qualitative stakeholder interviews conducted at the provider level, we aim to unravel the nuanced attitudes and perceptions that shape providers' decisions regarding HU prescription for SCD patients. The outcomes of these interviews will illuminate potential barriers hindering HU prescription. For instance, if concerns regarding medication costs emerge prominently during these qualitative sessions, we will actively address them by disseminating information that underscores the coverage of HU under the National Health Insurance Authority Act of 2022 (https://archive.gazettes.africa/archive/ng/2022/ng-government-gazette-dated-2022-05-24-no-95.pdf). We will explore the inner organizational context to adapt a tailored intervention, including examining the SCD management standard protocol at clinical sites, staffing data (i.e., numbers of nurses, hematologist, and non-hematologist physicians available), and provider prescription practices for HU. This phase of exploration promises to enrich our understanding of the specific contextual factors surrounding the initiation of provider training for HU in Nigeria. Providers and patients from diverse clinical sites will be recruited to participate, ensuring a comprehensive representation of perspectives.

Upon the completion of our qualitative exploration, we will transition to the Preparation Phase of EPIS. Drawing upon insights garnered from the Exploration Phase, we will curate evidence-based materials on HU, leveraging resources such as the American Society of Hematology (ASH) pocket guides for SCD management (https://www.hematology.org/education/clinicians/guidelines-and-quality-care/pocket-guides). In the Preparation Phase, we adapt the provider-level task-sharing implementation strategy. Our adaptive process ensures the tailored TASSH TCP strategy aligns seamlessly with the context-specific needs and challenges identified. Through this iterative refinement, we can fine-tune our approach to maximize impact and relevance within the unique landscape of Nigeria clinical sites. During this phase, we will meticulously chart out the training cycles for non-hematologist physicians and nurses across the 20 clinical sites. We will also train Practice Outreach Facilitators (POFs) whom will provide clinical support for the implementation of the intervention during this phase. These sites encompass a spectrum of healthcare facilities, ranging from tertiary-level institutions like the University of Abuja Teaching Hospital to primary care facilities including Primary Healthcare Centers (PHC). We will conscientiously identify convenient timings for providers and POFs to undergo training, ensuring optimal

participation, and subsequently develop a structured follow-up training schedule to sustain engagement and knowledge retention among participating providers.

During the Implementation Phase of EPIS, we will deploy our comprehensive SCD management and HU training program to providers across 20 clinical sites participating in a cluster RCT. Within this phase, providers will be randomly assigned to one of two arms within the intervention trial. The first arm will comprise physicians and nurses who will undergo specialized training in the administration of HU and SCD management. Additionally, these physicians and nurses will receive ASH pocket guides and will be supported by highly skilled nurses with extensive experience in both HU administration and SCD management at their local clinical sites. Furthermore, this intervention arm will benefit from periodic refresher courses on prescribing HU for SCD patients and providers will be supported by POFs. This arm will encompass 10 sites, including Primary Healthcare Centers (PHC) and Teaching Hospitals. The second arm, designated as the Control Arm, will consist of physicians and nurses randomly allocated across 10 sites. In this arm, healthcare providers will receive initial training on HU administration and relevant information such as ASH pocket guides. However, unlike the Intervention Arm, they will not receive ongoing support in the form of trained nurses. Throughout the study, investigators will evaluate the prescription behavior of physicians in both arms at 0 and 12 months across all 20 clinical sites. This analysis aims to discern whether the prescription of HU has increased within either or both arms of the cluster RCT.

Moving to the Sustainment Phase, following 12 months of providing support to the intervention arm, we will withdraw clinical support (i.e., remove POF from clinics/health facilities in the intervention arm) and highly trained nurses from this group. Subsequently, we will assess the prescription of HU at the 24-month mark. Sustainment or maintenance will be determined by comparing the prescription rates of HU between the periods of 12-24 months and the initial baseline of 0-12 months across all 20 clinical sites. We will also assess the implementation fidelity of the SIM intervention across all 20 clinical sites to determine whether differences in implementation of the intervention occurred based on-site specific factors.

**Duration of the project**

The project spans a 5-year duration in Abuja, Nigeria, with NYU exclusively receiving de-identified data from clinical sites and the University of Abuja. Pre-implementation endeavors are slated for years 1-2, encompassing exploration activities to grasp the implementation landscape, training study providers, and enrolling eligible SCD participants after securing NYU's IRB approval. Year 3 marks the commencement of intervention implementation, coinciding with a two-year cluster RCT. Year 4 focuses on sustaining the intervention at clinical sites, while year 5 sees ongoing data analysis across all sites. Implementation efforts persist for two years, entirely within Nigeria, with NYU serving as the data coordinating hub, ensuring meticulous oversight of all data management tasks while only accessing de-identified data. NYU personnel will visit Nigeria twice yearly; post-IRB approval and before study commencement, maintaining rigorous engagement throughout the study period.

### 5.2 Outcome Variables

#### 5.2.1 Primary Outcome Variables

Primary outcome measure is the rate of adoption of SIM at clinical sites at 12 months. This is a composite measure of adoption ratings to assess the degree to which the three essential elements of the SIM protocol (Screen, Initiate, and Maintain) are implemented at SPARC-NEt clinical sites. Thus, the primary outcome will be assessed at 12 months by the following measures:

- the number of SCD patients taking HU identified through screening,
- proportion of patients that on HU based on the REACH Clinical Trial algorithm tailored for aged 9 months through adulthood using SPARCO HU Guidelines, and
- proportion of patients who maintained dosage.

To assess these measures, we will use laboratory measures which include several hematological parameters to allow the attending physician (healthcare provider) to assess improvement of SCD on HU. Examples of these laboratory measure include: Hematocrit (%), Hemoglobin (g/dL), Leukocytes (cell/mm3), Neutrophil (cell/mm3), Platelets (cell/mm3), Fetal hemoglobin (%), Serum iron (µg/dL), Ferritin (ng/mL) and several other hematological variables that can improve once patients are on HU. We will also use physician, nurse, and pharmacist-completed questionnaires about newly diagnosed SCD patients, the retention of patients for follow-up at each study visits, and medication history. This analysis will be accomplished with a multilevel MANOVA (unstructured covariance matrix across two-time points baseline and 12 months). The analysis will have one within person factor — Time

(baseline and 12-month coded naturally as months (0 and 12)) and one primary between-patient factor (Randomization Group dummy coded as 0 = Control and 1 = TASSH TCP).

**5.2.2 Secondary and Exploratory Outcome Variables**

Secondary outcome measures are sustainability and implementation fidelity across the clinical sites at 12 and 24 months. The mediators measured via self-report are based on the constructs of the EPIS including inner context characteristics of the clinics, intervention characteristics, and implementation process measures. In brief, our hypothesis is that the sustainability and implementation fidelity of SIM will be higher in the sites randomized to TASSH TCP than those in control condition (no TASSH TCP). Sustainability is a key implementation outcome and a priority topic in implementation science. We will use the EPIS framework to evaluate the maintenance of SIM at participating health facilities over time. Sustainability will be assessed using a validated sustainability tool and qualitatively, based on interviews with nurses, physicians, clinic leadership, and patients from 12 to 24 months. Two research coordinators will conduct the interviews with two nurses, one physician, and one key leadership personnel at each facility.

Measuring implementation fidelity is crucial for assessing the extent to which an intervention is delivered as intended. One effective method involves developing a fidelity monitoring plan that outlines key components of the intervention and how they should be delivered. This plan typically includes detailed protocols, checklists, and guidelines for implementers to follow. Direct observation by trained assessors can provide valuable insights into the fidelity of intervention delivery, allowing for real-time feedback and adjustments. For the TASSH TCP, we will use self-report measures, checklist (see FORM 10). Implementation Fidelity) and document those encounters with session logs or audio/video recordings. The power of our approach is that it providers for regular fidelity assessments at various stages of implementation enable tracking of implementation fidelity over time and identification of areas for improvement. Ultimately, a comprehensive approach to measuring implementation fidelity ensures that the intervention is being implemented consistently and with integrity, maximizing its potential effectiveness.

We will also assess the extent to which inner setting variables (e.g., implementation leadership, implementation climate, and organizational culture) affect the degree of adoption of SIM and its sustainability at 24 months. We will pay particular attention to the pathways via which these variables influence the association of intervention and adoption implementation fidelity and sustainability outcomes. We will estimate a just-identified path model using the robust weighted least squares estimator to investigate relationships among the theoretical mediators of implementation climate, implementation leadership, organizational culture, organizational readiness to change, and external change agent support. Based on our conceptual model, we will test the direct effects from the theoretical constructs to the adoption components (individually). In addition to the direct effects, the indirect effects from each variable to adoption via inner setting variables will be estimated as the product of component direct effects and tested using bootstrapped 95% confidence intervals. Finally, we will estimate the direct effects of the predicted model of adoption on SCD outcomes. Predicted probabilities of the adoption and sustainability outcomes will be calculated from path model coefficients to elucidate the magnitudes of direct and indirect effects.

## 6. Study Population

### 6.1 Study Population

Participants (providers and patients)) will be recruited from all eligible clinical sites from the Primary Health Care Centres that are part of the Consortium for Newborn Screening in Africa (CONSA) in the Federal Capital Territory as well as tertiary hospitals that are part of the Sickle Pan African Research Consortium NigEria NEtwork (SPARC-NEt) in North Central Nigeria (See Table 1). Providers will be recruited via recruitment flyers. Patients will be recruited at their regularly scheduled clinical appointment. The Nigerian healthcare system is a three-tiered pyramidal health structure in which the primary-level healthcare centers/facilities occupy the bottom tier, the district hospitals at the secondary-level or middle tier with teaching hospitals at the tertiary-level or top tier. We have targeted district level hospitals because it is where most SCD patients in Nigeria seek primary care and also primary health centers (PHC). The sites that form the SPARC-NEt Nigeria network consisting of medical centers, teaching hospitals, general hospitals/facilities, and PHC will participate in this study.

| Table 1. List of Health Facilities in the Federal Capital Territory (FCT) |
| --- |

| Primary Health Care Centres Involved in Newborn Screening | | | |
|---|---|---|---|
| S/No | **Site** | **Number of Staff trained in NBS** | **Qualification of staff** |
| 1 | PHC Lugbe | 4 | RN, MSC Public Health, MLT, SLT |
| 2 | PHC Kuje | 3 | Ph. D Public Health, Diploma BSc |
| 3 | PHC Tunga Maje | 3 | Nurse/Midwife (CNO) CHEW, MLT |
| 4 | PHC DEI DEI | 10 | MBBS, MLT, MLT, BSc, SLT, Interns (5) |
| 5 | PHC Byahzim | 3 | MSc. MLT SLT |
| 6 | Gwagwalada Town Clinic | 3 | MBBS, CNO, CNO ACHO |
| 7 | PHC Karu | 3 | BSc, CHEW, MLT |
| 8 | St Mary Hospital | 3 | MBBS, RN ,RN |
| 9 | PHC Dutse Alhaji | 4 | MSc,MLT,MLT, SLT |
| 10 | PHC Dutse Garki | 4 | MLT, MCB, BSc Nursing. CNO |
| 11 | PHC Waru | 5 | BSc, MLT, CHEW, Nurse/Midwife, Intern |
| 12 | PHC Angwan Dodo | 3 | CHO, CHEW. RM/HE |
| 13 | PHC Mpape | 4 | Nurse/Midwife, MLT, SLT, Intern |
| 14 | PHC Dutse Makaranta | 3 | Nurse, Nurse, Intern |
| 15 | PHC Dagiri | 3 | RN, Nurses, Research Nurse |
| Tertiary Health Care Centres with Current SCD Clinics | | | |
| S/No | **Site** | **Number** of Staff Available/Specific for SCD Services | **Qualification of staff** |
| 16 | National Hospital Abuja | 34 | |
| 17 | Federal Medical Centre Keffi | 56 | MBBS, FWACP, RN, BSc Pharm. MLS |
| 18 | University of Abuja | 33 | MBBS, FWACP, RN, |

| | | | |
|---|---|---|---|
| | Teaching Hospital | | BSc Pharm. MLS, FWACS |
| 19 | Nyanya General Hospital | 5 | MBBS, FWACP, RN, |
| 20 | Jos University Teaching Hospital | 19 | MBBS, FWACP, RN, BSc Pharm. MLS, FWACS |

The clinical sites infrastructures are indicated in the table and have at least 2 Nurses employed and are able to participate in the SIM intervention. These are the sites for newborn screening in the Federal Capital Territory under the Consortium for Newborn Screening (CONSA) program as well as tertiary health care hospitals which are part of SPARC-NEt with pre-consented patients enrolled an electronic data base. These sites have a demonstrated capacity to recruit >100 patients (See Table 1). The CONSA NBS program requires staff training to provide hydroxyurea treatment as an important intervention

Each clinical site receiving the SIM+TASSH TCP intervention (n=10), a task force will be formed to support program implementation and it will include 1) leadership support (e.g., executive/clinical director) 2) performance feedback (provide feedback to nurses and physicians on using SIM, and 3) TCP strategy to support nurses and physicians in the delivery of care for SCD patients. All Study sites are part of the CONSA and SPARC-NEt which is led by Prof. Nnodu, a hematologist and renowned SCD specialist. The number of health care workers and their qualifications have been highlighted in Table 1, Having both PHCs as well as tertiary hospitals in this study is strategic. This will build the capacity of health care workers in primary health care centres to provide essential health services such as screening and treatment for non-communicable diseases including SCD in the communities close to where patients live in other to help achieve the health-related SDG goals 3. The tertiary hospitals have an average of 5 consultants, 10 resident doctors, 7 nurses and 6 laboratory Scientists in each of the facilities. Medical specialists such as cardiologists, nephrologists, orthopedic surgeons, and ophthalmologists are available in most of the centers forming part of the multidisciplinary team and we will also leverage the expertise of specialists within SPARC-NEt in the management of SCD especially when end-organ complications occur. Other health workers such as pharmacists, medical records officers, counsellors, social workers, physiotherapists, nutritionists, data entry staff, laboratory scientist are also involved in the management of the SCD and members of SPARC-NEt. SPARC-NEt has developed common protocols to leverage the expertise of various specialists within the consortium.

We will utilize this existing clinical infrastructure for our ACCELERATE study. Our investigators have significant experience working with the Nigerian healthcare system. The selected participating facilities are sites with active ongoing projects with demonstrative access to their patient populations. We will use several strategies to retain participants while they are enrolled in the study based on best practices from the literature. Currently (1) CESRTA has an MOU with the Federal Capital Territory Primary Health Care Board for the CONSA NBS program giving us access to designated PHCs with enough manpower to participate in the program.

**6.1.1 Number of Participants**

Eligible SCD patients (adults and children/ both male and females) will be identified from the CONSA NBS program and the SPARC-NEt Nigeria database. Once study is approved, potential study participants will be contacted by a nurse from each of the clinical sites during routine primary care visits with families. Each of the 20 sites will recruit 45 eligible patients for a total sample size of 900 who meet the following eligibility criteria over a 2-year period.

SCD Providers: Hematologist, Non-Hematologist physician, Nurse, Community health workers (2 per site) will participate in the training and delivery of the intervention.

**6.1.2 Eligibility Criteria**

***Inclusion criteria (Providers)***

- SCD provider (Hematologist, Non-Hematologist physician, Nurse, Community health workers) at a

participating clinical site;
- Have experience with managing SCD

***Exclusion Criteria (Providers)***
- Unable to participate in study activities

***Inclusion criteria (Patients)***
- Adult SCD patients age 18 years or older
- Pediatric SCD patients aged 12 months to 17 years with an accompanying guardian
- Willingness and ability to provide informed consent / assent
- Registration in the electronic medical records (EMR) database with clinical charts and received care at the local clinical sites or health facilities and not on HU therapy
- Hb Genotype: SCD-SS, SCD-Sβ° thal, SCD-$SO_{Arab}$ (On a case-by-case basis based on the physician assessment of the patient, a severely affected person with SCD-SC may be offered HU therapy under a modified treatment protocol)
- Clinical: None
- The adult patient, parent or legal guardian of the child patient must have demonstrated a high level of responsibility and must be regarded as capable of understanding the concepts of the therapy, follow the treatment guidelines, and be willing to comply with the required visits, laboratory evaluations, and schedule of medications. This is a provider level intervention, we will not assess parents and guardians except via focus groups and general follow-up questions during their regularly scheduled clinic visits.

***Exclusion criteria (Patients)***
- Any SCD patient not registered in the EMR database. We will only use the established SCD registry (EMR database) for recruitment.
- Pregnancy: Hydroxyurea has contraindications for pregnant women and could potentially affect the fetus as indicated by FDA labels and guidance for use from the American Society of Hematology)
- Physically unable to participate in study activities
- An SCD patient on HU
- Meets REACH exclusion criteria (NCT01966731)
    - Ongoing chronic transfusion therapy
    - Chronic utilization of medications that may enhance toxicities of HU
    - Concomitant chronic illness that has the potential to increase the toxicities of HU

**6.1.2 Vulnerable Populations**

Providers included in this study will provide SCD management to adults as well as children. HU is FDA-approved for use in children. HU is safe and has been shown to reduce: Vaso-occlusive crisis (severe blood clotting), blood transfusion, hospitalizations, incidents of acute chest syndrome (severe chest pain), improvements in organ function, and improvement's in overall survival for both children and adults. Inclusion of vulnerable populations, such as children, in research involving HU among patients with SCD is essential to ensure scientific validity and the applicability of study results to the broader patient population. SCD predominantly affects individuals from infancy through adulthood, with early childhood being a critical period for disease management and intervention. Excluding children from research involving HU could significantly compromise the scientific validity of the study and limit the generalizability of findings. Children with SCD often experience unique clinical manifestations and treatment responses compared to adults, necessitating tailored approaches to disease management. Moreover, excluding children from such research would raise ethical concerns regarding equitable access to potentially life-saving treatments and the advancement of pediatric healthcare. Therefore, the inclusion of vulnerable populations, including children, is justified to ensure comprehensive understanding and adoption strategies for management of SCD with HU therapy.

**7. Methods**

**7.1 Intervention**

**7.1.1 Description of Intervention**

The Nigerian government released guidelines supporting the SIM intervention for HU adoption for improved SCD management, and HU is on the list of essential medicines for Nigeria. Our implementation strategy for improving SCD management in Nigeria uses a practical and replicable evidence-based task-sharing strategy,

TAsk-Strengthening Strategy for Hemoglobinopathies (TASSH), adopted from our TAsk-Strengthening Strategy for Hypertension control (TASSH) trials in Ghana and Nigeria (led by Dr. Ogedgebe) containing the essential components of

- Training healthcare workers/providers to be more patient-centered in clinical consultations,
- Clinical reminders, and
- Practice facilitation (TCP) known as (TASSH TCP) for SCD management (See **Figure 1** below which describes the components of the intervention: **Figure 1. TASSH TCP intervention components and overall structure of implementation trial).**

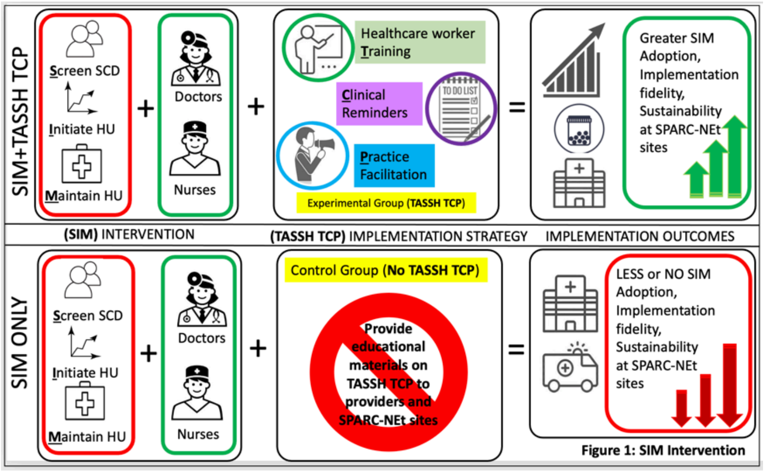

Figure 1: SIM Intervention

Healthcare providers, including physicians and nurses, will undergo comprehensive training to acquire knowledge about HU and SCD management. The training program is designed to be thorough yet efficient, with an initial didactic session expected to last for two hours. Subsequently, follow-up sessions will be conducted every six months throughout the first year of training. For physicians, a more in-depth portion of the training is anticipated, requiring approximately four hours to cover essential aspects comprehensively. This extended duration ensures that physicians receive detailed instruction and sufficient time for discussion and clarification on complex topics related to HU and SCD management. By investing in rigorous training sessions tailored to the specific needs of healthcare providers, we aim to equip them with the necessary knowledge and skills to effectively prescribe and manage HU therapy for patients with SCD, thereby enhancing overall patient care and outcomes.

For patients, no formal training will be necessary. Their interaction with healthcare providers will proceed as usual, with the added step of obtaining HU as prescribed. During each clinical visit, patients will engage in a brief question-and-answer session aimed at assessing their satisfaction with the provided service and their overall experience with HU therapy. This interactive session, designed to gather valuable feedback and ensure patient-centered care, is anticipated to last approximately one hour per visit.
This is a pragmatic provider-level intervention, it does not include formal training of patients and parents outside of physician/nurse contact with parents and SCD patients. This is the current system of standard of care for SCD in Nigeria. SCD patients are normally given pamphlets that is part of usual care in Nigeria for SCD education. We will adhere to these standard of care for SCD management. Essentially we want ensure that our provider-level training is embedded within the existing healthcare system, and aligns with existing standards of care without adding extraneous components which could be viewed as burdensome to patients, parents and providers. Currently, most physicians and nurses can ensure adequate patient education for SCD via existing materials.

**Components of the TASSH TCP implementation strategy.** Two trained nurses per site will implement the SIM protocol as POFs to train healthcare providers (e.g., nurses and non-hematologist physicians) to deliver SIM. The overall ratio for POF to clinical sites will be approximately 2:1. The POFs will be required to complete an intensive 12-week training course focused on adoption of the SIM protocol. Over the course of 12 months, the POFs will provide support to their assigned SPARC-NEt clinical sites to implement SIM as part of routine patient care using TASSH TCP. TASSH TCP will include four outreach facilitation visits to participating SPARC-NEt clinical sites with an educational learning session prior to the launch of the program and monthly telephone support provided by the facilitator. The POFs will help the providers 1) increase their confidence in SCD management strategies using SIM protocol, 2) understand patient concerns about HU therapy and laboratory monitoring, 3) provide counseling to patients for maintenance of HU SCD, and 4) create checklists and other resources as clinical reminders to support clinical decision making in SCD management in the implementation of the SIM protocol.

**SIM Protocol. Screen:** We will review the SPARC-NEt Nigeria database for eligible patients at 20 SPARC-NEt

clinical sites; eligible patients will be invited to participate in the study. **Initiate:** HU treatment will occur for all SCD patients in SPARC-NEt clinical sites based on a dosing calculator. **Maintain** and monitor HU dosage over time via laboratory monitoring and assessment of study measures (see Table 2)

**Conceptual Framework**

Using a sequential exploratory mixed-methods study design, we will conduct this study using EPIS framework to assess the adoption of SIM by providers in the context of the TASSH TCP implementation strategy in Nigeria (**See Figure 2** : **TASSH TCP Study designed aligned with EPIS framework)**

1. **Exploration Phase:** Determine the inner organizational context, outer context, and implementation processes likely to influence implementation and adoption of guidelines concordant care embedded within the TASSH TCP strategy to increase adoption of SIM among providers within clinical sites, assessment of facilitators, and barriers to implementation. Adapt the implementation strategy (e.g., TASSH TCP) using semi-structured interviews and focus group discussions with key stakeholders to assess the practice capacity for SIM adoption;

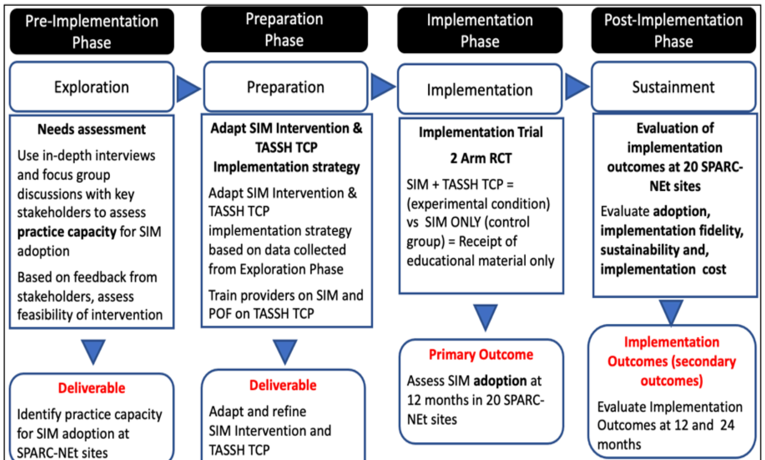


**Figure 2: EPIS Conceptual Framework**

2. **Preparation Phase:** Adapt the implementation strategy based on information from the Exploration Phase and modify the SIM protocol based on expert panel input (e.g., nurses, physicians, patients, other stakeholders) and train support staff including practice facilitators (i.e., POFs);
3. **Implementation Phase:** Assess the effect of TASSH TCP (experimental) vs. receipt of educational information only on task sharing (control) within a cluster RCT on SIM adoption at clinical sites at 12 months (primary outcome);
4. **Sustainment Phase:** Evaluate the sustainability and implementation fidelity of SIM at 24 months in clinical sites (one year after implementation) and finally create a SIM+TASSH TCP tool kit and disseminate our findings for clinical providers in Nigeria and other resource constrained settings.

**7.1.2 Method of Assignment/Randomization**

**Randomization** will occur at the clinical site level, with each facility randomized to the intervention arm (SIM+TASSH TCP) or control (SIM only*)*. The cluster randomization sequence will be computer generated (using SAS, Version 9 in accordance with CONSORT guidelines and overseen by Dr. Do. Sites will be informed of their randomization group by email. Because of the nature of the intervention, it is impossible to blind the patients, healthcare providers, and the study coordinators to the group assignment. Further, because of the pragmatic nature of this implementation trial, the PIs, and research staff will not be blinded.

**7.1.3 Selection of Instruments/Outcome Measures**

**Procedures following randomization:** Once a site is randomized, we will collect data on the primary outcome (adoption of SIM) from providers at clinical sites following the SIM training and at 12 months post-training and also from the other half receiving the control condition (SIM; no TASSH TCP). Information regarding adoption will be collected at baseline, 12-, and 24-months post-intervention initiation from all clinical sites (see Table 2 below for all study forms).

| **Table 2. ACCELERATE PRIMARY/ SECONDARY/ OTHER MEASURES** | |
|---|---|
| **Primary Outcome Measure** | **Assessment Tool** |
| Primary outcome measure is the rate of adoption of SIM at Clinical sites at 12 months. (Screening, | FORM 9. Adoption |

| | |
|---|---|
| Initiate and Maintain) | |
| **Secondary outcome Measure** | |
| Secondary outcome measures are the mediators of SIM adoption including sustainability and implementation fidelity across the SPARC-NEt sites at 12 and 24 months. | FORM 10. Implementation Fidelity<br>FORM 11. Sustainability Assessment Tool<br>FORM 12. Scalability Assessment Tool |
| **Other measures (potential mediators)** | |
| Practice Capacity Assessment | FORMS 8A-8H. Practice Capacity and Implementation Science Scales |
| Implementation Context | FORM 7. Focus Group Discussion Guide |
| Patient-level Surveys | FORMS 1-6B<br>FORM 1. Participant Information<br>FORM 2. Behavioral Measurements includes medication history and Study Medication (HU) Prescription and Dates log<br>FORM 3. Physical health measurements<br>FORM 4. Biochemical Measurements<br>FORM 5. Barriers / Facilitators to HU Uptake<br>FORM 6A-6B. Quality of Life Assessment (Adult and Child) |

**7.1.4 Intervention Administration**
**Clinical Reminders**
As part of the TASSH TCP training, the study providers will receive printed HU pocket guides as clinical reminders tailored for the local context to facilitate SCD management. The Hydroxyurea and Transfusion Therapy for the Treatment of Sickle Cell Disease clinical reminder will be adopted from the American Society of Hematology guidelines (https://apps.hematology.org/store/Product.aspx?productid=98175591).

Hydroxyurea is FDA approved and it is used to prevent painful episodes and reduce the need for blood transfusions in patients with sickle cell anemia. It works by making the red blood cells more flexible (https://www.fda.gov/drugs/resources-information-approved-drugs/fda-approves-hydroxyurea-treatment-pediatric-patients-sickle-cell-anemia).

**Administration of HU Dosage**
The administration of HU in this research study will be conducted in alignment with standard clinical care protocols established for patients with SCD. Adherence to established clinical guidelines ensures the safety and well-being of participants while maintaining consistency with real-world treatment practices. By adhering to established protocols for HU administration, including dosage, monitoring, and management of potential adverse effects, the study aims to replicate the conditions under which patients with SCD receive treatment in routine clinical settings will assessing our implementation outcomes. Additionally, strict adherence to clinical care standards mitigates potential risks associated with HU therapy and fosters trust and confidence among participants in the research process,

**HU Administration and Follow-up Protocol**
**Physical examination procedure**
Clinical evaluation / Screening
1. Completion of History Form
2. Completion of detailed Physical Exam Form
3. Completion of Growth Chart (in patients up to age 18 years)

**Follow up details if required**

Monitoring of HU Therapy

Patients will be monitored as part of usual care through two types of outpatient visits (Interim Visits and Blood Count Visits).

1. Interim Visits: Interim Visits will occur at 12-week intervals and will coincide with routine visits to the patient's regular SCD doctor, or nurse. During this visit, the Interim Visit Form will be completed.
1. Blood Count Visits: These will occur every four weeks after initiation of HU therapy to ensure there are no specific side effects. Thereafter, these visits will occur every 12 weeks in conjunction with the Interim Visits until a change in schedule is necessitated by toxicity or other indications.

The following studies will be obtained on Blood Count and Interim Visits according to the following schedule:

1. FBC, diff & retic
   a. every 4 weeks, in patients for at least 8 weeks and those undergoing dose modification for hematologic toxicity, and,
   b. every 12 weeks, in patients for at least 8 weeks to assess hematologic toxicity
2. Chemistry panel
   a. every 24 weeks
3. Hb F Studies (Hb F%, F-cell % [if available])

- Every 24 weeks

**Laboratory procedure to be used**
Laboratory evaluation

Samples must be collected BEFORE HU therapy is initiated and at least 12 weeks after the last RBC transfusion.

1. Complete (Full) Blood Count CBC), platelet count, UNCORRECTED reticulocyte count, and WBC differential. (Calculate absolute reticulocyte count [ARC] and absolute neutrophil count [ANC])
2. Comprehensive metabolic profile, including renal and liver function tests: at least, Cr, BUN, ALT, total and fractionated bilirubin, alkaline phosphatase.

Results of these two sets of tests must be reviewed before the child is started on HU therapy.

1. Hb F studies: Hb F%, Hb F-cell % (if available)
2. Urinalysis
3. Serum pregnancy test for females who have achieved menarche but not menopausal

**Project Management**
**Principal Investigators / Co-Investigators and Responsibilities:**

Prof. Gbenga Ogedegbe, MD, MPH, MS (PI NYUSOM): Dr. Ogedegbe will work with Dr. Peprah to provide oversight of all scientific, programmatic, financial, and administrative matters for this proposed contract. He will oversee development and execution of annual Project Management plans and pipeline budgets, effectively coordinate implementation of project activities, track project milestones and project deliverables in close collaboration with the Principal Investigators. He will support documentation and dissemination of project performance to the Funder and other stakeholders.

Dr. Emmanuel Peprah: As PI, NYUGPH, Dr. Peprah will lead / oversee the development and execution of the implementation strategy including 1.) semi-structured interviews informed by an EPIS interview guide among clinical sites supervisors and directors, physicians, nurses, and patients) at participating facilities and 2.) Focus group sessions with an equal mix of provider representation from the various clinical sites. These two activities will systematically codify enablers and barriers to successful HU adoption that have been observed in Nigeria among providers to date, including lessons learned from clinical staff at clinics and hospitals. The qualitative data collection will enable development of the TASSH-TCP intervention. He will monitor implementation of the intervention at sites in Nigeria and assess "real-time" as HU for sickle cell disease (SCD) begins to be administered at small scale in Nigeria and evaluate the clinical outcomes and implementation outcomes among patients and providers respectively.

Prof. Obiageli Nnodu, MD: As contact-PI in-country, Prof. Nnodu will lead engagements with key stakeholders

including the Ministry of Health (at National/ Provincial/ District levels) and at SPARC-NEt clinical sites, University of Abuja Teaching Hospital, and other Health Care Providers. She will oversee and conduct patient recruitment in line with implementation strategy, systematically codify enablers and barriers to successful implementation of HU guidelines that have been observed in Nigeria to date, including baseline data collection lessons learned from clinical staff at implementing sites. She will assess and monitor "real-time" as HU for sickle cell disease (SCD) begins to be administered at small scale in Nigeria and integrate those lessons into the CMA; also explore how the digital Mobile HU-Treatment Management application that is in development can best be applied to improve implementation and monitoring of the national scale-up activities. She will devise a draft evidence-based roadmap for successful HU implementation in district hospitals/implementing sites and clinics in Nigeria. She will assist in the development and buy-in of the SCD patient and provider application for SCD management in Nigeria. She will assist with the implementation, monitoring, iterative refinement, and impact evaluation of the roadmap, including when larger scale use of HU begins in Nigeria.

Dr. Hyungrok Do, PhD (Co-Investigator, Biostatistician): Dr. Do will oversee randomization, REDCap (Research Electronic Data Capture) database development, quality control of data collection and entry, and analysis. He will also advise on data analysis and reporting.

Dr. Joyce Gyamfi, Ed.D, MS (Co- Investigator): Dr. Gyamfi will lead the development of survey instruments for data collection, analysis, monitoring and reporting of outcome-level project deliverables. She will oversee the data collection team and work with the Nigeria-based investigators to tailor the quantitative and qualitative data collection instruments needed for the study, provide strategies for stakeholder engagement, guide written reports and manuscripts obtained from research activities. She will assist Drs. Peprah and Ogedegbe with the execution of the overall implementation strategy, monitoring and reporting of project deliverables to funder, documenting lessons learnt and troubleshooting when needed.

Other Study Personnel
<u>University of Abuja</u>
Professor Maxwell Nwegbu (Co-Investigator), Chemical Pathologist, former Dean of the Faculty of Basic Clinical Sciences University of Abuja, Chair of Skill Development, SPARC-NEt, Laboratory Lead CONSA Newborn Screening Laboratory and Laboratory Lead of clinical trials at CESRTA. He will be responsible for the skills training for nurses, clinicians and residents in hematology and pediatric health care workers on hydroxyurea in the secondary and tertiary health care facilities. He will also coordinate the clinical chemistry tests required for patient monitoring.

Dr. Hezekiah Isa (Co-Investigator), is a hematologist at University of Abuja Teaching Hospital (UATH) and the SPARC-NEt Nigeria Site Coordinator with experience in multisite projects. He will ensure the proper daily administration of the ACCELERATE study including patient recruitment, Practice Outreach Facilitators (POF) training, and adherence to study timelines and monitoring adverse outcomes by coordinating laboratory measures (e.g. hemoglobin level) and health outcomes (e.g., hospitalizations, pain crisis, acute chest syndrome, etc.), including physician and nurse completed questionnaires and the retention of patients for follow-up.

Dr. Dike Ojji (Co-Investigator), is Chair of Internal Medicine and an Associate Professor of Preventive Cardiology at College of Health Sciences, University of Abuja, and the Lead Investigator, Cardiovascular Research Unit, University of Abuja. He was also the Deputy Directors, Centre for Undergraduate Research and the Institute for Advanced Medical Research and Training both of University of Abuja. Dike also holds an Honorary Visiting Professorship position at Cape Heart Institute, Department of Medicine, University of Cape Town, Cape Town, South Africa. His work spans defining the spectrum of hypertensive heart disease and hypertension pharmacotherapy in the black population, to establishing a system for hypertension and cardiovascular care in the primary care level in low- and middle- income countries to informing national dietary sodium policy. He has been involved in several multi-centre studies and led the CREOLE trial in 10 sites in 6 sub—Saharan African countries, the result of which was presented at the 2019 American College of Cardiology Congress Late Breaking Clinical Session and published simultaneously in the New England Journal of Medicine. He is currently the multiple principal investigator for 5 NIH research and training grants, some in collaboration with New York University. He has published >125 manuscripts in high-impact journals including *New England Journal of Medicine, Nature Medicine, JAMA*, and the *Lancet*. As a global cardiovascular health epidemiologist at UATH with expertise in clinical trials, will ensure that all sites reach study milestones or resolve issues via corrective action plans.

*NYU*

**Project Manager (TBD)**

Research Coordinator of the ACCELERATE study will be based at the Centre of Excellence for Sickle Cell Disease Research and Training, University of Abuja. Research Coordinator will work closely with the Co-PI to ensure the proper day-to-day administration of the study and grant management and prepare drafts of reports for the project.

John Patena, DrPH student
Tania Hameed, MPH, Graduate student

### 7.1.5 Reaction Management

All clinical care and management of patients will be conducted by their treating physician as part of standard of care, not as part of this study.

## 7.2 Assessments

### 7.2.1 Efficacy

**Survey Data Collection**

The Nigerian-based research team will administer patient-specific surveys (see Table 2): Participants who are SCD patients or guardians/caretakers to SCD patients and providers will complete surveys to assess patients' quality of life and self-efficacy in managing SCD. Majority of the information needed for the patient survey can be obtained from the patient medical records once authorization is provided by the patient. Patient experience surveys (Forms 5, 6A-6B) will take no more than 45 minutes in total to complete. Data from the surveys will inform the study aims of assessing HU adoptions and or mediators of HU adoption.

Survey data will be collected in-person, de-identified and recorded electronically into REDCap (Research Electronic Data Capture), which is a web-based application. The surveys created on REDCap can also be administered offline when internet connectivity is limited or unavailable. The data will be stored on the device (laptop or tablet) and will upload automatically into the cloud storage of REDCap once the device is connected to the internet. REDCap is secure and designed to support data capture for research studies. It provides a stream-lined process for rapidly building a database; an intuitive interface for collecting data, with data validation; automated export procedures for seamless data downloads to common statistical packages like SPSS, SAS, Stata, and R); advanced features, such as branching logic, file uploading, and calculated fields and audit trails for tracking data manipulation and export procedures. When electronic collection of data is not feasible, paper form will be used, and later transferred into the electronic database.

### 7.2.2 Safety

**Assessment of Safety Based on Risks**

Hydroxyurea (HU) therapy stands as a beacon of hope in Nigeria's standard care for sickle cell disease (SCD) patients, yet its full potential remains untapped due to gaps in provider training. Despite its remarkable tolerability and minimal short-term risks, such as rare occurrences of carcinogenesis, reversible oligospermia, and reactivation of latent tuberculosis, alongside commendable patient compliance, some individuals experience side effects like skin rashes, darkened patches, vomiting, and dizziness. To safeguard participant privacy, rigorous measures, including obtaining written consent and ensuring anonymity in transcripts, will be upheld throughout surveys and interviews.

Guided by the FDA and American Society of Hematology (ASH), our approach to HU administration adheres closely to established protocols, bolstered by ASH pocket guides tailored for SCD patients. Safety evaluation will be twofold: firstly, vigilant monitoring of short-term toxicity based on documented side effects, as endorsed by the ASH guidelines outlined in the pocket guides; second, addressing non-compliance through comprehensive provider training. Recognizing the critical role of healthcare providers, we will offer repeated training sessions, ensuring all necessary educational components are acquired for effective intervention delivery.

However, in instances where providers fail to complete the requisite educational training for the task-sharing intervention, sites will be omitted from the study to maintain fidelity to established protocols. This stringent approach guarantees adherence to the highest standards of study integrity, reinforcing the commitment to realizing the full potential of HU therapy in Nigeria.

### 7.3 Study Procedures
### 7.3.1 Study Schedule
**Provider Training**

The TASSH TCP (experimental group) will receive training at baseline with booster training every 3 months for 1 year (12 months) on HU prescription and maintenance in SCD patients. The educational information only on TASSH TCP (control group) will receive information only on task sharing but not TASSH TCP training/facilitation. To eliminate cost, as a potential confounder to HU access, all patients will be referred to a SPARC-NEt provider for HU medication and receive regular laboratory monitoring at no cost. Pharmacies at each clinical site will be provided with monthly questionnaires to assess HU utilization among patients and physician prescriptions during the study.

**Patient study visits.** After patient eligibility is confirmed by the ACCELERATE study team (study coordinator and nurse) all patient visits will be scheduled to align with existing clinical visit protocols. Study investigators (Drs. Nnodu, Agumadu) will review qualitative data collection. Baseline visit: All SCD patients have clinical records in the SPARC-NEt clinical records database; patients informed consent will be reviewed; once eligibility is confirmed, the site nurse will conduct baseline assessments of SCD, comorbidity, and qualitative data collection. Patients will then be given the study visit schedule and instructions. Primary outcome data will be collected from all participants. SCD patient measures. The patient level data are necessary to measure the impact of the SIM adoption and healthcare quality. Study teams will collect measures every 6 months from patients and caregivers (i.e., baseline, 6, 12, 18, and 24 months). The Pediatric Quality of Life Survey assesses pediatric patients' quality of life, specifically using the PedsQL$^{TM}$ SCD module scales to measure children's physical, emotional, and social functioning. The Adult Quality of Life Survey uses the Adult Sickle Cell Quality of Life Measurement Information System (ASCQ-Me) to assess physical, mental, and social well-being of adults with SCD. A Brief Pain Inventory uses a numerical rating scale to assess adult pain intensity.

### 7.3.2 Informed Consent
After patient eligibility is confirmed by the ACCELERATE study team (study coordinator and nurse),
all patients will be consented / assented prior to any study activities. Potential pediatric participants who may be able to take part in this study are unable to give consent because they are under 18 years of age (a minor). Instead, we will ask their parent(s) or legal guardian to give consent. We will also ask the minor to agree (give their assent) to take part in the study. They will be given an Assent Form to sign. Patient visits will be scheduled to align with existing clinical visit protocols. Study investigators (Drs. Nnodu, Peprah, Ogedegbe) will review procedures for all data collection.

Eligible participants will be asked to sign the consent forms prior to participation in the study. Voluntary participation will be further emphasized at this stage of the study. All participants will be reminded that their responses to study questionnaires are confidential and that they may refuse to participate in the project or withdraw at any time without explanation, and further, that such an action will in no way affect their treatment with their physician at NYULH. Also, if a participant is uncomfortable during a research encounter, they may stop at any time. Consent will be conducted in English as English is the official language in Nigeria. Study nurse coordinators will conduct the inform consents. However, professional translation will be provided in a Nigerian local dialect to accommodate patients who are not proficient in English. Subjects who turn 18 while in the study will be re-consented as adults during their next clinic appointment.

All key personnel in the study are trained and certified in ethical aspects of medical research involving human subjects and good clinical practices, with certification meeting standards of the NIH.

### 7.3.3 Screening
Participants will be recruited at their regularly scheduled clinical appointment. Once the participant agrees to participate in the study, a trained study nurse will schedule a time with eligible interested participants to go over the written consent in-person. Study staff will provide in person or email a copy of the consent to participants to go over in preparation for the consent. After going over the written consent, the Nurse will answer any questions the participant may have about the study. They will also remind participants that they can halt the consent process at any time, and return later, if they need time to think about their participation.

### 7.3.4 Recruitment, Enrollment and Retention

Participants will be recruited from all 20 SPARC-NEt sites. Participants will be recruited at their regularly scheduled clinical appointment by the Study Nurse. The Nigerian healthcare system is a three-tiered pyramidal health structure in which the primary-level healthcare centers/facilities occupy the bottom tier, the district hospitals at the secondary-level or middle tier with teaching hospitals at the tertiary-level or top tier. We have targeted the district level hospitals, because it is where most SCD patients in Nigeria seek primary care. The sites that form the SPARC-NEt Nigeria network consisting of medical centers, teaching hospitals, general hospitals/facilities will participate in this study.

The clinical sites infrastructure are indicated in Table1 and have at least 2 Nurses employed and are able to participate in the SIM intervention because each facility has >300 patients pre-consented in the SPARC-NEt database and have a demonstrated capacity to recruit >100 patients .

Each clinical site receiving the SIM+TASSH TCP intervention (n=10), a taskforce will be formed to support program implementation and it will include 1) leadership support (e.g., executive/clinical director) 2) performance feedback (provide feedback to nurses and physicians on using SIM, and 3) TCP strategy to support nurses and physicians in the delivery of care for SCD patients. All Study sites are part of the SPARC-NEt which is led by Dr. Nnodu, a hematologist and renowned SCD specialist. There is an average of 5 consultants, 10 resident doctors, 7 nurses and 6 laboratory Scientists in each of the facilities (See Table 1). Medical specialists such as cardiologists, nephrologists, orthopedic surgeons and ophthalmologists are available in most of the centers forming part of the multidisciplinary team and we will also leverage the expertise of specialists within SPARC-NEt in the management of SCD especially when end-organ complications occurs. Other health workers such as pharmacists, medical records officers, counsellors, social workers, physiotherapists, nutritionists, data entry staff, laboratory scientist are also involved in the management of the SCD and members of SPARC-NEt. SPARC-NEt has developed common protocols to leverage the expertise of various specialists within the consortium. We will utilize this existing clinical infrastructure for our ACCELERATE study. Moreover, our Nigerian collaborators have significant experience working with the Nigerian healthcare system and are supportive of this proposal (see biosketchs and letters of support). The selected participating facilities have similar facility and patient characteristics based on analysis of the SPARC-NEt database.

**Retention Plan:** We will use several strategies to retain participants while they are enrolled in the study based on best practices from the literature. These include: (1) Signed memorandum of understanding (MOU) from each SPARC-NEt site to support the ACCELERATE study: We have found that this formal agreement ensures that the clinical sites understand the purpose of the study and their roles and responsibilities for participation. The MOU also highlights the benefits of participating in the study. All participating clinical sites will be asked to sign this agreement as part of the enrollment process. (2) Identify a champion or key contact to act as a liaison: This is also crucially important to ensure retention of participants in the study. (3) Offer monetary incentives for participation: Participants will receive a stipend for their time in the study, in line with applicable regulations of Nigeria. (4) Maintain communication: Because this is a prospective study, ongoing communication with study participants will be needed throughout year 1 (via phone calls and emails) to collect qualitative data; after year 1, we will also use medical records for follow-up of clinical outcomes for patients.

### 7.3.5 Study Visits: Monitoring of HU Therapy as a component of usual care

Baseline visit:

- All SCD patients have clinical records in the SPARC-NEt clinical records database;
- Patients SPARC-NEt informed consent will be reviewed;

Once eligibility is confirmed:

- The site nurse will conduct baseline assessments of SCD, comorbidity, and qualitative data collection.
- Patients will then be given the study visit schedule and instructions.

Patients will be monitored through two types of outpatient visits, Interim Visits and Blood Count Visits.

1. *Interim Visits*: Interim Visits will occur at 12-week intervals and will coincide with routine visits to the patient's regular SCD doctor, or nurse. During this visit, the Interim Visit Form will be completed.
2. *Blood Count Visits*: These will occur every four weeks after initiation of HU therapy to ensure there are no

specific side effects. Thereafter, these visits will occur every 12 weeks in conjunction with the Interim Visits until a change in schedule is necessitated by toxicity or other indications. A sample collection of blood about 10mL (adults) or 3mL (children) will be collected during each visit. Blood samples will be collected via venipuncture, utilizing either the antecubital or dorsal vein, aligning with the standard protocol recommended for patients as young as 12 months in Nigeria. While venipuncture is the preferred method, acknowledging that for certain children, especially those for whom a finger-prick might be more suitable, this method could be considered optimal. Therefore, we will defer to the discretion of the physician or nurse to determine the most appropriate method, considering caregiver/guardian preferences or cultural considerations, while prioritizing sensitivity to the child's comfort is paramount throughout the process. Adherence to the World Health Organization Guidelines on Drawing Blood: Best Practices in Phlebotomy ensures standardize procedures for blood draws in pediatric patients, with consideration given to factors such as age, weight, and potential hindrances like calluses on the feet for finger-prick selection. The process of performing venipuncture on children aged 12 months and older demands not only technical proficiency but also a compassionate, patient-centered approach. Following the guidelines endorsed by the Paediatric Association of Nigeria (PAN), local protocols will be followed with blood draws done by trained physicians or nurses. The volume of blood collected will be minimized to reduce the risk of adverse effects, typically ranging from 0.5 milliliters (mL) to 3 mL for routine laboratory tests like complete blood count (CBC), based on individual clinical assessment and test requirements. Thorough preparation, including clear communication and positioning, will help with vein selection, while the use of antiseptic swabs and optional topical anesthesia reduces discomfort. Skillful needle insertion, coupled with strict monitoring of blood flow and gentle pressure, stabilizes the vein during the procedure. Post-procedure care, as indicated by PAN guidelines, emphasizes measures to promote hemostasis, proper disposal of sharps, and comprehensive instructions for follow-up care, with rigorous documentation ensuring accuracy and accountability throughout.

### 7.3.6 End of Study and Follow Up

As our study nears its conclusion, meticulous planning for the End of Study and Follow-Up phase becomes paramount to ensuring comprehensive data collection and participant welfare. For end of study procedures will include several critical components, including final data collection sessions, participant debriefing, and any necessary post-intervention assessments. We will ensure clear communication with participants regarding the study's conclusion and any ongoing support or resources available to them essential for maintaining ethical standards and participant trust (e.g., national health insurance can be provided from two years after study completion). Moreover, the follow-up period offers an invaluable opportunity to assess the longer-term impacts of the intervention, providing insights into its sustainability and enduring effects. We will ensure robust data management practices during this phase, including data cleaning and analysis, which is essential for deriving meaningful conclusions from the study outcomes. Additionally, documenting any challenges encountered during the follow-up period can inform future research endeavors and enhance the overall quality of scientific inquiry. Through diligent planning and execution of the End of Study and Follow-Up phase, we will maximize the study's impact and contribute valuable insights to the field.

### 7.3.7 Removal of Subjects

As part of the process involved in obtaining written informed consent, all participants will be reminded that their responses are confidential and that they may refuse to participate in the project or withdraw at any time without explanation, and further, that such an action will in no way affect their treatment with their physician or employment at the participating practices. If a participant is uncomfortable during a research encounter, they may stop at any time.

In case of pregnancy, HU therapy should be discontinued. Post-pubertal males on HU should be advised against making a female partner pregnant.

## 7.4 Statistical Method

### 7.4.1 Statistical Design

**Quantitative Data**: All data will be summarized and presented in tabular and graphical format, using means, standard deviations, medians, and ranges for continuous variables and proportions for categorical variables. We will assess balance with respect to baseline characteristics according to randomized treatment assignment. In the outcome analysis, we will adjust for any baseline characteristics for which balance was not achieved. Analyses

will be conducted according to the principle of intention-to-treat, in which every clinic is analyzed according to the assigned intervention.

Data for these analyses will be the entire sample of 20 clinical sites with SIM+ TASSH TCP and SIM only. Specifically, the 24-month data from the SIM only dataset will be combined with the baseline data from the SIM+TASSH TCP dataset and the 12- month data from the SIM+TCP group will be combined with the 24-month data from the SIM only group, creating a data set where change on the Adoption Ratings from Baseline to 12 months will be assessed in the entire sample. We anticipate that this larger dataset of the two groups will allow us to measure change in Adoption (the dependent variable) and calculate the difference (change) from baseline to 12 months for each clinical site. We have previously conducted this analysis on our TASSH hypertension study in Ghana and found it informative by regressing the change score on to the proposed mechanisms (i.e., leadership engagement, implementation climate, and organizational capacity). We found that a significant positive relation between the mechanisms and the change score indicated that change was associated with greater leadership engagement, implementation climate, and organizational capacity.

The primary outcome measure will be the composite rating of the three components of SIM (Screen, Initiate, and Maintain). Multilevel modeling software (SAS, Version 9, PROC MIXED) will be used to compute full information maximum likelihood (FIML) estimates of the model parameters.

The PROC MIXED procedure will use an error structure that allows for the possibility of group differences in (a) the error variances at 12 months? and (b) the serial correlations of the baseline with the 12-month outcomes. The primary test is the Group X Time interaction, and the resulting F-test will provide the primary "intent to treat" test of the hypothesis. If this is statistically significant at the two tailed $\alpha=.05$ level, for ease of interpretation, we will estimate and report the magnitude of the treatment effect, with 95% CI for Adoption.

Ideally, the randomization of participants to treatment groups will obviate the need for any covariates in the analysis. However, in the event that baseline differences between the patients in each group on the outcomes, demographic, or secondary measures are found, those variables will be included as covariates in the MANOVA (including their interactions with time).

**7.4.2 Sample Size Considerations**

The intervention will be delivered through group sessions at clinical sites where the unit of randomization is the hospital or clinic; ten in the intervention arm and ten in the control arm (n=20). Based on previous literature, implementing SIM to address SCD could have approximately 19% difference in the rates of composite measure of adoption between the treatment arms. Thus, we estimate sample sizes for a range of small differences (minimum detectable difference (MDD) between 7.5% to 10.5%, assuming standard deviation of 30% or Cohen's d between 0.25 and 0.35) for this intervention, assuming an average class size ($m$) of about 45 participants/clinic, and intra-class coefficients (ICC)s between .01 and .06. This yields a total sample size of 20 x 45 = 900 participants, providing more than 80% power to detect a small difference of 9% with a conservative assumption of ICC = 0.02. Note that this includes adjustment for up to 30% attrition of the study sample; this estimate is conservative.

40 providers from the 20 sites (2 per site) will be included in the study. Having two providers from each study site will ensure continuum of care when there is staff tun-over.

**7.4.3 Planned Analyses**

**7.4.3.1 Primary Analyses**

**Primary Outcome Analysis**

Primary outcome measure is the rate of adoption of SIM at SPARC-NEt clinical sites at 12 months. This is a composite measure of adoption ratings to assess the degree to which the three essential elements of the SIM protocol (Screen, Initiate, and Maintain) are implemented at SPARC-NEt clinical sites. Thus, the primary outcome will be assessed at 12 months by the following measures:

- the number of SCD patients taking HU identified through screening,
- proportion of patients that on HU based on the REACH Clinical Trial algorithm tailored for aged 9 months through adulthood using SPARCO HU Guidelines, and
- proportion of patients who maintained dosage.

To assess these measures, we will use laboratory measures, physician, nurse, and pharmacist-completed questionnaires about newly diagnosed SCD patients, the retention of patients for follow-up at each study visits, and medication history. This analysis will be accomplished with a multilevel MANOVA (unstructured covariance matrix across two-time points baseline and 12 months). The analysis will have one within person factor — Time (baseline and 12-month coded naturally as months (0 and 12)) and one primary between-patient factor (Randomization Group dummy coded as 0 = Control and 1 = TASSH TCP).

**7.4.3.2 Secondary Objectives Analyses**
Secondary outcome measures are the mediators of SIM adoption, implementation fidelity, and sustainability across the clinical sites at 12 and 24 months. The following measures will be used to assess the mediators of SIM via self-report. The mediators are based on the constructs of the EPIS including inner context characteristics of the clinics, intervention characteristics, and implementation process measures. In brief, our hypothesis is that the sustainability and implementation fidelity of SIM will be higher in the sites randomized to TASSH TCP than those in control condition (no TASSH TCP). Sustainability is another key implementation outcome and a priority topic in implementation science. Our approach is to use the EPIS framework to evaluate the maintenance of SIM at participating health facilities over time. The outcomes for this aim will be the same as the measures used for the adoption outcomes in the Implementation Phase.

We will evaluate the factors that mediate the effect of TASSH TCP implementation strategy on adoption, implementation fidelity and sustainability of the SIM intervention at 12 and 24 months. In particular, we will assess the extent to which inner setting variables (e.g., implementation leadership, implementation climate, and organizational culture) affect the degree of adoption of SIM and its sustainability at 24 months. We will pay particular attention to the pathways via which these variables influence the association of intervention and adoption implementation fidelity and sustainability outcomes. We will estimate a just-identified path model using the robust weighted least squares estimator to investigate relationships among the theoretical mediators of implementation climate, implementation leadership, organizational culture, organizational readiness to change, and external change agent support. Based on our conceptual model, we will test the direct effects from the theoretical constructs to the adoption components (individually). In addition to the direct effects, the indirect effects from each variable to adoption via inner setting variables will be estimated as the product of component direct effects and tested using bootstrapped 95% confidence intervals. Finally, we will estimate the direct effects of the predicted model of adoption on SCD outcomes. Predicted probabilities of the adoption and sustainability outcomes will be calculated from path model coefficients to elucidate the magnitudes of direct and indirect effects.

**7.4.3.3 Analysis of Subject Characteristics**

**Analysis of Quantitative (Clinical) Data**
The following indicators will be retrieved from SCD patients' medical records including sickle cell type, laboratory results, comorbidities, pain crisis, hospitalization, medication history and other related SCD management indicators. Data will be de-identified and entered into REDCap database. Data analysis will be conducted using SPSS statistical software. Descriptive statistics including frequencies and means will be generated and reported in a tabular format for aggregate data. Any associations will be explored using the chi-square or Fisher exact test (p values at 0.05) where applicable.

Data analysis will be conducted using SPSS statistical software. Descriptive statistics including frequencies and means will be generated and reported in a tabular format for aggregate data. Any associations will be explored using the chi-square or Fisher exact test (p values at 0.05) where applicable.

We will also assess the following systems-level measures to gain insight into the implementation setting / context (health facilitates) and the barriers and facilitators to implementation. These scales will be administered to clinical site providers (Forms 8A-8H, 9-12).

Systems-level (practice capacity) and organizational-level measures

Data collection for study outcomes will occur every 6 months (i.e., baseline, 6, 12, 18 and 24 months) using instruments described below.

1. Organizational Capacity for Change is a multidimensional scale that evaluates an organization's capacity

to upgrade or revise existing organizational competencies (α=0.87),[58] with a five-point Likert scale. We will adapt the 27-item multi-dimensional measures to assess capacity for organizational change.

2. The Implementation Leadership Scale has excellent reliability and convergent/discriminant validity. It is a brief 12-item measure with four subscales: Proactive Leadership (α=0.95), Knowledgeable Leadership (α=0.96), Supportive Leadership (α=0.95), and Perseverant Leadership (α=0.96) and a total score (α=0.98).[59] Both leadership and staff version of the scale will be used.
3. The Implementation Climate Scale measures shared perceptions of the policies, practices, procedures, and behaviors that are expected, supported, and rewarded to facilitate effective EBP implementation (overall α=0.91). It has six subscales (2-3 items each): Focus on EBP (α=0.91), Educational Support for EBP (α=0.84), Recognition for EBP (α=0.88), Rewards for EBP (α=0.81), Selection for EBP (α=0.89), and Selection for Openness (α=0.91).[60]
4. The Organizational Culture domain (i.e., proficient culture) of the Organizational Social Context Scale is a 15-item proficiency subscale to evaluate practice capacity proficiency (α =0.89).[61] Proficient Organizational Cultures,[62] is characterized by shared norms and expectations that healthcare providers prioritize each patient's well-being, are skilled service providers, and have current SIM knowledge.
5. The Evidence-based practice attitude scale is a 15-item measure which assesses the attitudes towards the adoption of evidence-based practices.[63,64]

**Analysis of Qualitative Data**

(1) In the Exploration Phase, we will use the data collected from the focus group and semi structured interviews to determine the inner organizational context, outer context, and implementation processes likely to influence SIM implementation within clinical sites, assessment of facilitators, and barriers to implementation, and develop the multimodal evidence-based implementation strategy using in-depth interviews and focus group discussions with key stakeholders to assess the practice capacity for adoption of SIM and assess the feasibility of the intervention;

(2) In the Preparation Phase, we will develop implementation strategy based on information from Exploration Phase and modify the SIM protocol based on expert panel input (e.g., nurse, physicians, patients, stakeholders) and user testing of the developed TASSH TCP implementation strategy.

The semi-structured interviews and user-testing interviews will be transcribed and entered into NVivo, version 14.0, for data analysis. We will use the framework approach to qualitative data analysis a five-step process that involves;

- familiarization (a process during which the researcher becomes immersed in the details of multiple sources of data to gain a general understanding of the content and document initial impressions);
- developing a theoretical framework (a process by which the researcher identifies emergent themes in the multiple sources of data using existing theories as a guide). These themes will be continually refined and compared to each other;
- indexing (during which the researcher becomes further immersed in the data in order to refine identified themes and sub-themes);
- summarizing data in an analytical framework (during which the researcher reduces materials into understandable, but brief summaries of what was said by stakeholders); and
- data synthesis and interpretation (which allows for comparison of themes and sub-themes against original transcripts, field notes, and audio recordings to ensure appropriate context.

Following the framework approach, the data will be independently coded by the two to three research members to reduce the potential for bias. Inter-rater reliability will be determined based on a sub-set of the data (e.g., the interviews) and will be repeated until satisfactory agreement among raters is achieved (i.e., 80% of coded data). Discrepancies in coded data will be resolved by consensus. After systematically reading all transcripts, data will be coded into concepts reflecting the aim of this phase. For example, responses will be coded according to provider-level factors (e.g., knowledge of SIM toolkit) and issues related to practice capacity with using the SIM toolkit in clinical sites (e.g., logistics/resources involved). Established procedures to enhance the trustworthiness of our analysis will be used, including analyses of codes that do not fit our coding scheme, development of an audit trail documenting analytical decisions, and member-checking presentations to the Advisory Board. The identified concepts will then be grouped into categories, and themes uniting the categories will be determined. A detailed analysis of the interviews should generate a conceptual model that elucidates barriers to the uptake of the SIM toolkit within clinical sites and modified context tailored TASSH TCP implementation strategy to overcome

these challenges.

#### 7.4.3.6 Other
### 7.4.4 Subsets and Covariates
### 7.4.5 Handling of Missing Data
Longitudinal designs tend to have missing data due to attrition; we anticipate minimal missing data for the adoption rating as the data will be obtained from staff at clinical sites. Using the maximum likelihood multilevel modeling approach to estimate intervention effects, we will include data from all staff and SCD patients enrolled in the trial (intent-to-treat), even if some of the patients' data are missing.

# 8. Trial Administration
## 8.1 Ethical Considerations: Informed Consent/Assent and HIPAA Authorization
A risk of the study is the potential inappropriate dissemination of personal contact information of participants. However, NYU and the Abuja team members have received the NIH training on maintaining security precautions to prevent the release of this information. This project has obtained ethical approval from the University of Abuja and the Nigerian Health Research Ethics Committees. All patients will be consented / assented prior to any study activities.

All key personnel in the study are trained and certified in ethical aspects of medical research involving human subjects and good clinical practices, with certification meeting standards of the NIH. The study will be conducted in compliance with the protocol and the following recommendations and guidelines: The Declaration of Helsinki (Scotland Revision, 2000) International Conference on Harmonization Guidelines as outlined in the Belmont Report would be followed. Identifying information will be collected from participants for the sole purpose of being able to contact participants to schedule study sessions. Identifying information would include names, phone numbers, age, and profession.

## 8.2 Institutional Review Board (IRB) Review
IRB approval will be sought prior to any data collection involving human subjects for this study.

**Unanticipated Problems and Protocol Deviations**

For unanticipated problems and protocol deviations the Program Manager will contact the Principal investigator and will follow-up with the participant's primary care provider to ensure proper medical follow-up. It will be documented in an adverse event log. IRB will be notified if necessary. These data will be reviewed periodically by the OSMB and will be reported to the IRB.

**Study Modification and Discontinuation**

Although all of the decisions on scientific direction will be made jointly by Drs. Ogedegbe, Peprah, and Nnodu, the lead role in the decision-making process will be reflective of the major responsibilities for each PI. The PIs have forged an optimal working relationship in which their interaction resulted in substantial synergism. Significant decisions related to research prioritization, substantial reallocation of funding (greater than 25% of direct costs), termination of underperforming research project, and intellectual property will be informed by extensive input from NIH program officials, as appropriate. Drs. Ogedegbe, Peprah, and Nnodu will work together to achieve consensus on final decisions related to implementation and scientific direction, hiring/termination of non-key personnel, and budget reallocations.

IRB will be notified and must approve all modifications to the study. IRB and Sponsor will be notified and must approve any discontinuation to the study.

## 8.3 Subject Privacy, Confidentiality & Data Management
Private identifiable information from participants such as their names, email addresses and telephone numbers will be kept in a separate document that will be password protected and stored in an encrypted computer file, on a password protected laptop, with anti-virus and anti-spyware protection. Participants' identifiers will also be de-identified by assigning ID codes to participants, to protect participants' responses from being traced back to any particular participant.

Data will be collected in-person and recorded electronically by research staff on to REDCap at CESRTA and the

University of Abuja, a web-based application for data capture and management. All study activities will be conducted in a private space, which does not pose a risk or discomfort to study participants. Conducive space will be identified by the Nigeria-based collaborators.

Identifiable and de-identified survey responses from this study will be deleted from all folders and devices after three years, post study completion; hard copies of consent forms and survey forms will also be shredded three years after study completion.

ID Assignment: Data collection forms will be identified only with IDs; relating of ID code to names will require information kept under lock and key and supervised by a designated high-level staff member. Additionally, none of the analyses will permit identification of any individual.

Data Storage and Security: This study will be performed in strict accordance with the standards for protection of privacy of identifiable health information. The principal investigator will ensure security and confidentiality of all study records. Hard copies of study participants data will be kept in Nigeria and maintained in locked cabinets, and access to the records will be restricted to specific study team members only. Electronic study data stored in Nigeria will be protected via password access only to study investigators and staff and protected via a secure cloud-based storage system with end-to-end encryption. All study data transferred to NYU will be password protected and encrypted. All computers (both in the US and Nigeria) involved in the collection and saving of participants' information will be password protected and will be on a private LAN network. The computers' LAN network will have a hardware-based firewall separation that protects against hackers and unauthorized access to all electronic data not maintained on the server. This will provide protection against viruses, worms and Trojan horses transmitted over the Internet. Spam and email filtering will also be built-in within the firewall device. The firewall will contain anti-virus software (McAfee Antivirus) to protect the network from threats of viruses contained in email attachments. Through "push-technology" this anti-virus software will automatically be updated for all virus definitions and other updates. We will maintain all study records for the maximum period as required by international (such as the NIH of USA) and local regulatory guidelines.

Data will be de-identified and will be entered into REDCap Consortium, which currently comprises 352 active institutional partners from CTSA, GCRC, and other institutions from around the world. REDCap is a secure, web-based application designed to support data capture for research studies. It provides a stream-lined process for rapidly building a database; an intuitive interface for collecting data, with data validation; automated export procedures for seamless data downloads to common statistical packages like SPSS, SAS, Stata, and R; advanced features, such as branching logic, file uploading, and calculated fields and audit trails for tracking data manipulation and export procedures.

**8.4 Deviations/Unanticipated Problems**

For unanticipated problems and protocol deviations the Program Manager will contact the Principal investigator and will follow-up with the participant's primary care provider to ensure proper medical follow-up. It will be documented in an adverse event log. IRB will be notified if necessary. The data will be reviewed periodically by the OSMB and will be reported to the IRB.

**8.5 Data Collection**

The Nigerian-based research team will administer patient-specific surveys (see Table 2): Participants who are SCD patients or guardians/caretakers to SCD patients and providers will complete surveys to assess patients' quality of life and self-efficacy in managing SCD. Majority of the information needed for the patient survey can be obtained from the patient medical records once authorization is provided by the patient. Patient experience surveys (Forms 5, 6A-6B) will take no more than 45 minutes in total to complete.

Survey data will be collected in-person every 6 months during the study, de-identified and recorded electronically into REDCap (Research Electronic Data Capture), which is a web-based application. The surveys created on REDCap can also be administered offline when internet connectivity is limited or unavailable. The data will be stored on the device (laptop or tablet) and will upload automatically into the cloud storage of REDCap once the device is connected to the internet. REDCap is secure and designed to support data capture for research studies. It provides a stream-lined process for rapidly building a database; an intuitive interface for collecting data, with data validation; automated export procedures for seamless data downloads to common statistical packages like

SPSS, SAS, Stata, and R); advanced features, such as branching logic, file uploading, and calculated fields and audit trails for tracking data manipulation and export procedures. When electronic collection of data is not feasible, paper form will be used, and later transferred into the electronic database.

**8.6 Data Quality Assurance**

Data quality is ensured through data validation at several stages, including initial training on the REDCap data forms, and sample data checks for missing and or incomplete data on an ongoing basis. We will review the data to identify potential discrepancies and problems with data quality issues in a timely manner. Data quality for patient- and staff-reported data will be ensured through rigorous data collection training procedures overseen by Drs. Ogedegbe, Peprah, Nnodu, and Gyamfi based on their previous studies; and ongoing data collection checks and reviews conducted by the study coordinators, with refresher trainings as necessary.

**8.7 Study Records**

We will also obtain patient level data from electronic health records (EHR) for patients who meet eligibility criteria and receive care at the participating clinical sites. We will follow recommendations made for the responsible conduct and protection of human subjects in qualitative or non-randomized research and cluster randomized trials. We plan to further coordinate and share best practices, research services, and resources with investigators and staff.

**8.8 Access to Source**

**8.9 Data Storage/Security**

**8.10 Retention of Records**

The study results will be kept in our research record for at least six years or until after the study is completed, whichever is longer. At that time either the research information not already in participants medical record will be destroyed or information identifying the participant will be removed from such study results at NYU. Any research information in participants medical record will be kept indefinitely.

**8.11 Study Monitoring**

Observational Safety and Monitoring Board (OSMB) The principal investigators will establish an Observational Safety and Monitoring Board (OSMB) as the principal monitor to ensure that the study is conducted with highest standards and ethically sound.

**8.12 Data Safety Monitoring Plan**

In compliance with NIH requirements, an independent Observational Safety and Monitoring Board (OSMB) will be established for this study in quarter 2 of year 2 This OSMB will be composed of independent faculty members from the ACCELERATE project, a collaborative endeavor among multiple partners including University of Abuja (UA), New York University School of Global Public Health (NYUGPH), and New York University School of Medicine who are not otherwise involved, are willing to participate, and who have no conflict with serving on such a board. Membership of the OSMB will include, but not be limited to, the following expertise: An expert in the conduct of clinical trials; three professionals with substantive expertise in the area of SCD (one physician, one hematologist, one Ph.D.), a biostatistician with expertise in clinical trials, and an Implementation Scientist.

The OSMB will convene prior to initiation of the study, at the time that the interim data analysis is available, and when the final data analysis for this study is available. They will meet at least once a year. Scheduled interim analyses will be performed by the study biostatisticians and supplied to the OSMB for review. If the difference in outcomes (i.e., a composite measure of SIM intervention [providers are not screening patients for SCD, or initiating HU treatment for eligible SCD patients] at baseline to 12 months) between intervention and control arms across the various sites exceeds the a priori limits defined by the study biostatisticians and the OSMB, the project steering committee has the authority and obligation to terminate the study.

Should the contrast in outcomes, encompassing a composite measure of one or more sickle cell-related adverse effects (such as increased pain crises, occurrences of acute chest syndrome, hospital admissions, and instances of blood transfusions) among patients on hydroxyurea (HU) from baseline to the 12-month, vary significantly between the intervention and control groups across diverse study sites, surpassing predetermined thresholds established for each patient based on their medical history as assessed by the study's biostatisticians, a decision regarding the continuation of the study will be made. These predetermined thresholds will be set in accordance

with the average incidence of adverse events (pain crises, acute chest syndrome, hospital admissions, blood transfusions) observed in sickle cell disease (SCD) patients not receiving HU, as deduced from existing medical records. For instance, the average occurrence of blood transfusions ranges from 1 to 2, as documented in previous research,[65] thereby informing the computation of an average blood transfusion rate for each patient not on HU based on their electronic medical records. Should patients receiving HU surpass the predefined thresholds for adverse events (e.g., pain crises, acute chest syndrome, hospital admissions, blood transfusions), the project steering committee holds the authority and responsibility to terminate the study.

The OSMB will also receive all Serious Adverse Event (SAE) Reports and may request additional information, as needed. If the OSMB deems that an unexpected number of SAEs have been reported, they may choose to meet off-cycle to assess the events and determine what action should be taken. In addition, on-site monitoring visits from a qualified research monitor will be scheduled quarterly until data quality is deemed acceptable and then will be scheduled every six months for the remainder of the study. The Research Coordinator at UA will work with the Research Manager and Data Manager to complete the following tasks:

- Preparation of all reports
- Distribution and presentation of reports to the Principal Investigators and the OSMB
- Evaluation and recommendation of modifications to recruitment strategies to assure consistency of procedures
- Evaluation of recruitment status, considering balance of gender, and age subgroups
- Design and maintenance of the study's administrative internal computer server (dataset, reports, minutes, and documentation access)
- Development of a standard description of recruitment procedures for use in study manuscripts
- Coordination of training of site staff and investigators in data acquisition and human subjects protection
- Support of Principal Investigators with required analyses and reports
- Collaboration on publications and presentations with statistical assistance
- Participation in committees as required to facilitate study coordination

**8.13 Study Modification**

In the event that modifications to the study become necessary, our commitment to ethical research practice dictates prompt action. Any proposed modifications will undergo thorough evaluation and documentation before being promptly resubmitted to the NYU SOM Institutional Review Board (IRB) for approval in additional to the National Health Research Ethics Committee and the University of Abuja Teaching Hospital Health Research Ethics Committee in Nigeria for review and approval. Our team recognizes the paramount importance of maintaining the integrity of the study while ensuring the safety and well-being of all participants involved. By adhering to rigorous procedural standards and promptly addressing any required adjustments, we uphold the highest ethical standards and demonstrate our unwavering dedication to the principles of responsible research conduct.

**8.14 Study Discontinuation**

**8.15 Study Completion**

The expected completion date of the study is 08/2028 IRB and Sponsor will be notified of completion of the study through final progress reports.

**8.16 Conflict of Interest Management Plan**

A robust Conflict of Interest Management Plan is indispensable for upholding the ethical integrity of research endeavors, ensuring that potential conflicts are identified and addressed effectively to safeguard the objectivity of the study. Our meticulously crafted multi-PI conflict resolution and conflict of interest plan sets stringent standards for all Principal Investigators (PIs) and co-investigators, mandating the disclosure of any conflicts that may arise during the study's duration. This comprehensive plan encompasses regular disclosure requirements, intricate mechanisms for reviewing and resolving conflicts, as well as clear protocols for recusal or mitigation measures should conflicts arise among study investigators. Transparency is paramount within our study framework, with an unwavering commitment to communicating potential conflicts openly and transparently to all relevant stakeholders, including esteemed funders such as the National Institutes of Health (NIH), participants, and oversight bodies such as Institutional Review Boards (IRBs). Through the rigorous implementation of our Conflict of Interest Management Plan, we uphold the highest standards of integrity, ensuring the credibility and reliability of our research outcomes.

### 8.17 Funding Source

National Institutes of Health (NIH) / NHLBI.

### 8.18 Publication Plan

Peer-reviewed publications will be submitted to relevant journals. We plan to publish the study protocol, baseline findings, and outcome results.

## REFERENCES


1. Adewoyin AS. Management of sickle cell disease: a review for physician education in Nigeria (sub-saharan Africa). Anemia. 2015;2015:791498.
2. Chakravorty S, Williams TN. Sickle cell disease: a neglected chronic disease of increasing global health importance. Arch Dis Child. 2015;100(1):48-53.
3. Piel FB, Hay SI, Gupta S, Weatherall DJ, Williams TN. Global burden of sickle cell anaemia in children under five, 2010–2050: modelling based on demographics, excess mortality, and interventions. PLoS medicine. 2013;10(7):e1001484.
4. Ohene-Frempong K, Oduro J, Tetteh H, Nkrumah F. Screening newborns for sickle cell disease in Ghana. Pediatrics. 2008;121(2):S120.
5. Brown BJ, Okereke JO, Lagunju IA, Orimadegun AE, Ohaeri JU, Akinyinka OO. Burden of health-care of carers of children with sickle cell disease in Nigeria. Health Soc Care Community. 2010;18(3):289-295.
6. Corbacioglu S. Sickle cell disease. Paper presented at: ONCOLOGY RESEARCH AND TREATMENT2016.
7. Chaturvedi S, DeBaun MR. Evolution of sickle cell disease from a life-threatening disease of children to a chronic disease of adults: The last 40 years. Am J Hematol. 2016;91(1):5-14.
8. Grosse SD, Odame I, Atrash HK, Amendah DD, Piel FB, Williams TN. Sickle cell disease in Africa: a neglected cause of early childhood mortality. American journal of preventive medicine. 2011;41(6):S398-S405.
9. Isa H, Adegoke S, Madu A, et al. Sickle cell disease clinical phenotypes in Nigeria: A preliminary analysis of the Sickle Pan Africa Research Consortium Nigeria database. Blood Cells Mol Dis. 2020;84:102438.
10. Oluwole EO, Adeyemo TA, Osanyin GE, Odukoya OO, Kanki PJ, Afolabi BB. Feasibility and acceptability of early infant screening for sickle cell disease in Lagos, Nigeria-A pilot study. PLoS One. 2020;15(12):e0242861.
11. Nnodu OE, Adegoke SA, Ezenwosu OU, et al. A Multi-centre Survey of Acceptability of Newborn Screening for Sickle Cell Disease in Nigeria. Cureus. 2018;10(3):e2354.
12. Nnodu O, Isa H, Nwegbu M, et al. HemoTypeSC, a low-cost point-of-care testing device for sickle cell disease: Promises and challenges. Blood Cells Mol Dis. 2019;78:22-28.
13. Oron AP, Chao DL, Ezeanolue EE, et al. Caring for Africa's sickle cell children: will we rise to the challenge? BMC Med. 2020;18(1):92.
14. Ware RE. Hydroxycarbamide: clinical aspects. C R Biol. 2013;336(3):177-182.
15. Cunningham-Myrie C, Abdulkadri A, Waugh A, et al. Hydroxyurea use in prevention of stroke recurrence in children with sickle cell disease in a developing country: A cost effectiveness analysis. Pediatr Blood Cancer. 2015;62(10):1862-1864.
16. Dehury S, Purohit P, Patel S, et al. Low and fixed dose of hydroxyurea is effective and safe in patients with HbSbeta(+) thalassemia with IVS1-5(G-->C) mutation. Pediatr Blood Cancer. 2015;62(6):1017-1023.
17 Galadanci N, Wudil BJ, Balogun TM, et al. Current sickle cell disease management practices in Nigeria. International health. 2014;6(1):23-28.
18 Esezobor CI, Akintan P, Akinsulie A, Temiye E, Adeyemo T. Wasting and stunting are still prevalent in children with sickle cell anaemia in Lagos, Nigeria. Italian Journal of Pediatrics. 2016;42(1):45.
19. Adegoke S, Adeodu O, Adekile A. Sickle cell disease clinical phenotypes in children from South-Western, Nigeria. Nigerian Journal of Clinical Practice. 2015;18(1):95-101.
20. Adewoyin AS, Oghuvwu OS, Awodu OA. Hydroxyurea therapy in adult Nigerian sickle cell disease: a monocentric survey on pattern of use, clinical effects and patient's compliance. Afr Health Sci. 2017;17(1):255-261.
21. Adeyemo TA, Diaku-Akinwunmi IN, Ojewunmi OO, Bolarinwa AB, Adekile AD. Barriers to the use of hydroxyurea in the management of sickle cell disease in Nigeria. Hemoglobin. 2019;43(3):188-192.
22. Carey PJ. Addressing the global health burden of sickle cell disease. Int Health. 2014;6(4):269-270.

23. Ambrose EE, Makani J, Chami N, et al. High birth prevalence of sickle cell disease in Northwestern Tanzania. Pediatr Blood Cancer. 2018;65(1).
24. Ebomoyi EWJIJoME, Informatics. Ethical, legal, social, and financial implications of neonatal screening for sickle cell anaemia in Sub–Sahara Africa in the age of genomic science. 2015;7(1):46-56.
25 Adegoke SA, Macedo-Campos RS, Braga JAP, Figueiredo MS, Silva GS. Changes in Transcranial Doppler Flow Velocities in Children with Sickle Cell Disease: The Impact of Hydroxyurea Therapy. Journal of Stroke and Cerebrovascular Diseases. 2018;27(2):425-431.
26. Tshilolo L, Tomlinson G, Williams TN, et al. Hydroxyurea for Children with Sickle Cell Anemia in Sub-Saharan Africa. New England Journal of Medicine. 2019;380(2):121-131.
27. Nnodu OE. Interventions for the prevention and control of sickle cell disease at primary health care centres in Gwagwalada Area Council of the Federal Capital Territory, Nigeria. . Cureus 2014;6(8).
28. Amendah DD, Mukamah G, Komba A, Ndila C, Williams TN. Routine paediatric sickle cell disease (SCD) outpatient care in a rural Kenyan hospital: utilization and costs. PLoS One. 2013;8(4):e61130.
29. Aliyu ZY, Babadoko A, Mamman A. Hydroxyurea Utilization in Nigeria, a Lesson in Public Health. Blood. 2007;110(11):80.
30. Charache S, Terrin ML, Moore RD, et al. Effect of Hydroxyurea on the Frequency of Painful Crises in Sickle Cell Anemia. New England Journal of Medicine. 1995;332(20):1317-1322.
31. Okocha EC, Gyamfi J, Ryan N, et al. Barriers to Therapeutic Use of Hydroxyurea for Sickle Cell Disease in Nigeria: A Cross-Sectional Survey. Front Genet. 2021;12:765958.
32. WHO. World Health Statistics 2015. http://www.who.int/gho/publications/world_health_statistics/2015/en/. Accessed September 12, 2016.
33. Kalipeni E, Semu LL, Mbilizi MA. The brain drain of health care professionals from sub-Saharan Africa: A geographic perspective. Progress in Development Studies. 2012;12(2-3):153-171.
34. Ogedegbe G, Plange-Rhule J, Gyamfi J, et al. A cluster-randomized trial of task shifting and blood pressure control in Ghana: study protocol. Implement Sci. 2014;9:73.
35. WHO. Task shifting to tackle health worker shortages. World Health Organization. http://www.who.int/healthsystems/task_shifting_booklet.pdf. Accessed October 20, 2016.
36. Gyamfi J, Allegrante JP, Iwelunmor J, et al. Application of the Consolidated Framework for Implementation Research to examine nurses' perception of the task shifting strategy for hypertension control trial in Ghana. BMC Health Serv Res. 2020;20(1):65.
37. Blackstone S, Iwelunmor J, Plange-Rhule J, et al. Sustaining Nurse-Led Task-Shifting Strategies for Hypertension Control: A Concept Mapping Study to Inform Evidence-Based Practice. Worldviews Evid Based Nurs. 2017;14(5):350-357.
38. Gyamfi J, Plange-Rhule J, Iwelunmor J, et al. Training nurses in task-shifting strategies for the management and control of hypertension in Ghana: a mixed-methods study. BMC Health Serv Res. 2017;17(1):104.
39. Gyamfi J, Plange-Rhule J, Iwelunmor J, et al. Erratum to: Training nurses in task-shifting strategies for the management and control of hypertension in Ghana: a mixed-methods study. BMC Health Serv Res. 2017;17(1):216.
40. Iwelunmor J, Gyamfi J, Plange-Rhule J, et al. Exploring stakeholders' perceptions of a task-shifting strategy for hypertension control in Ghana: a qualitative study. BMC Public Health. 2017;17(1):216.
41. Ogedegbe G, Gyamfi J, Plange-Rhule J, et al. Task shifting interventions for cardiovascular risk reduction in low-income and middle-income countries: a systematic review of randomised controlled trials. BMJ Open. 2014;4(10):e005983.
42. Ogedegbe G, Plange-Rhule J, Gyamfi J, et al. Health insurance coverage with or without a nurse-led task shifting strategy for hypertension control: A pragmatic cluster randomized trial in Ghana. PLoS Med. 2018;15(5):e1002561.
43. Adewoyin AS, Alagbe AE, Adedokun BO, Idubor NT. Knowledge, Attitude and Control Practices of Sickle Cell Disease among Youth Corps Members in Benin City, Nigeria. Ann Ib Postgrad Med. 2015;13(2):100-107.
44. Adoukonou TA, Vallat JM, Joubert J, et al. [Management of stroke in sub-Saharan Africa: current issues]. Rev Neurol (Paris). 2010;166(11):882-893.
45. Adeodu OO, Alimi T, Adekile AD. A comparative study of perception of sickle cell anaemia by married Nigeria rural and urban women. West Afr J Med. 2000;19(1):1-5.
46. Gyamfi J, Ojo T, Iwelunmor J, et al. Implementation Science Research for the Scale-up of Evidence-Based Interventions for Sickle Cell Disease in Africa: A Commentary. Global Health,

Epidemiology and Genomics 2020.
47. Awofeso N. Improving health workforce recruitment and retention in rural and remote regions of Nigeria. Rural Remote Health. 2010;10(1):1319.
48. News B. Does Nigeria have too many doctors to worry about a 'brain drain'? https://www.bbc.com/news/world-africa-45473036. Accessed.
49. Creswell JW, Zhang W. The application of mixed methods designs to trauma research. J Trauma Stress. 2009;22(6):612-621.
50. Amico KR, Harman JJ, Johnson BT. Efficacy of antiretroviral therapy adherence interventions: a research synthesis of trials, 1996 to 2004. Journal of Acquired Immune Deficiency Syndromes. 2006;41(3):285-297.
51. American Diabetes Association; the National Heart L, and Blood Institute; the Juvenile Diabetes Foundation International; the National Institute of Diabetes and Digestive and Kidney Diseases; and the American Heart Association. Diabetes mellitus: a major risk factor for cardiovascular disease. Circulation. 1999;100(10):1132-1133.
52. Shelley DR, Ogedegbe G, Anane S, et al. Testing the use of practice facilitation in a cluster randomized stepped-wedge design trial to improve adherence to cardiovascular disease prevention guidelines: HealthyHearts NYC. Implementation Science. 2016;11(1):88.
53. Baskerville NB, Liddy C, Hogg W. Systematic review and meta-analysis of practice facilitation within primary care settings. *The Annals of Family Medicine.* 2012;10(1):63-74.
54. Pantoja T, Opiyo N, Lewin S, et al. Implementation strategies for health systems in low-income countries: an overview of systematic reviews. *Cochrane Database of Systematic Reviews.* 2017(9).
55. Lang ES, Wyer PC, Haynes RB. Knowledge translation: closing the evidence-to-practice gap. *Ann Emerg Med.* 2007;49(3):355-363.

56. Berta W, Cranley L, Dearing JW, Dogherty EJ, Squires JE, Estabrooks CA. Why (we think) facilitation works: insights from organizational learning theory. *Implementation Science.* 2015;10(1):1.
57. Lessard S, Bareil C, Lalonde L, et al. External facilitators and interprofessional facilitation teams: a qualitative study of their roles in supporting practice change. *Implementation Science.* 2016;11(1):97.
58. Judge W. Organizational change capacity: the systematic development of a scale. *Journal of Organizational Change Management.* 2009;22(6):635-649.
59. Aarons GA, Ehrhart MG, Farahnak LR. The Implementation Leadership Scale (ILS): development of a brief measure of unit level implementation leadership. *Implement Sci.* 2014;9(1):45.
60. Ehrhart MG, Aarons GA, Farahnak LR. Assessing the organizational context for EBP implementation: the development and validity testing of the Implementation Climate Scale (ICS). *Implement Sci.* 2014;9:157.
61. Glisson C, Landsverk J, Schoenwald S, et al. Assessing the organizational social context (OSC) of mental health services: implications for research and practice. *Adm Policy Ment Health.* 2008;35(1-2):98-113.
62. Glisson C, Williams NJ, Green P, Hemmelgarn A, Hoagwood K. The organizational social context of mental health medicaid waiver programs with family support services: implications for research and practice. *Adm Policy Ment Health.* 2014;41(1):32-42.
63. Aarons GA. Mental health provider attitudes toward adoption of evidence-based practice: the Evidence-Based Practice Attitude Scale (EBPAS). *Ment Health Serv Res.* 2004;6(2):61-74.
64. Armenakis AA, Bernerth JB, Pitts JP, Walker HJ. Organizational Change Recipients' Beliefs Scale: Development of an Assessment Instrument. *The Journal of Applied Behavioral Science.* 2007;43(4):481-505.
65. Diaku-Akinwumi IN, Abubakar SB, Adegoke SA, Adeleke S, Adewoye O, Adeyemo T, Akinbami A, Akinola NO, Akinsulie A, Akinyoola A, Aneke J. Blood transfusion services for patients with sickle cell disease in Nigeria. International Health. 2017 Sep 1;8(5):330-5.
